# Cantharidin-Based Verbenone Derivatives as a Novel Insecticide against *Plutella xylostella*: Design, Synthesis, Insecticidal Activity Evaluation, and 3D QSAR Study

**DOI:** 10.3390/biom13081272

**Published:** 2023-08-21

**Authors:** Kwanshik Lee, Hossam Nada, Minkyoung Kim, Hyejun Park, Kiho Lee, Dongho Seo, Kyeong Lee, Yongseok Choi

**Affiliations:** 1 College of Life Sciences and Biotechnology, Korea University, 145 Anamro, Seungbukgu, Seoul 02841, Republic of Korea; light@korea.ac.kr (K.L.); baddd1@korea.ac.kr (H.P.); 2 College of Pharmacy, Dongguk University, 32 Donggukro, Ilsandonggu, Goyangsi 10326, Republic of Korea; hossam_hammouda@dgu.ac.kr (H.N.); kyoung2k@naver.com (M.K.); 3 College of Pharmacy, Korea University, 2511, Sejongro, Sejong 30019, Republic of Korea; kiholee@korea.ac.kr (K.L.); hojoungg@korea.ac.kr (D.S.)

**Keywords:** verbenone, cantharidin, insecticide, diamondback moth, synthesis, 3D QSAR

## Abstract

The diamondback moth is a detrimental insect pest of brassicaceous crops which was among the first crop insects to be reported as DDT resistant. It has since proven to be significantly resistant to nearly every synthetic insecticide used in the field in many crucifer-producing regions. Due to insecticide control failures in some parts of the world, economically viable crucifer production is now all but impossible. As a result, there has been an increasing effort to identify new compounds with strong pesticidal activity. Cantharidin is one such compound that has been shown to be highly effective against a variety of insect pests. However, its chemical synthesis and potential toxicity to non-target organisms have been a major source of concern. Herein, using rational design approaches, a new series of cantharidin-based verbenone derivatives were synthesized and evaluated for their insecticidal activities against the diamondback moth. Among different compounds screened, compounds **6a**, **6h**, **6i**, and **6q** emerged as the most potent compounds exhibiting 100% mortality at a concentration of 100 mg/L after four days. These compounds demonstrated a good anti-feeding effect against the diamondback moth on cabbage leaves. Subsequently, a 3D QSAR study was carried out to identify the key structural features of the synthesized compounds and their correlation with insecticidal activity.

## 1. Introduction

*Plutella xylostella* (L.), also known as the diamondback moth (DBM), is one of the most catastrophic insect pests in the world. It was the first crop insect to be reported to be resistant to DDT [1]. DBM has since demonstrated significant resistance to nearly every insecticide used in field applications, including biopesticides like crystal toxins from *Bacillus thuringiensis* and spinosyns from *Saccharopolyspora spinosa* [2]. Due to its insecticide resistance and subsequent control failure, the economically viable production of crucifers has become nearly impossible in some regions of the world, necessitating the development of new insecticides capable of managing the DBM [3].

Bioinsecticides based on natural toxins are an alternative method of pest control that has been reported to possess promising potential to combat insecticide resistance and reduce our reliance on conventional insecticides [4]. Cantharidin is one such bioinsecticide and is a naturally occurring toxin produced by beetles of the Meloidae family [5]. It has long been used in folk and traditional medicine, particularly for the topical treatment of viral skin infections like warts and molluscum. Cantharidin has been shown to induce apoptosis in numerous cancer cell lines, showing good anticancer activity. However, its use in Western medicine has been constrained by serious side effects like renal toxicity [6,7].

Mammalian and plant protein phosphatase 1 (PP1) and protein phosphatase 2A (PP2A) activity has been shown to be inhibited in vivo by cantharidin and norcantharidin [8]. The inhibition of serine/threonine protein phosphatases appears to have played a key role in the herbicide mode of action of endothall, a cantharidin analog, in recent years [9]. This relationship was further confirmed by a study showing that cantharidin inhibitors lead to the decrease of protein serine/threonine phosphatase production in *P. xylostella*. Furthermore, it was found that the activities of rPxPP5 (a recombinant protein produced artificially from the five phosphatase enzymes (PP1, PP2A, PP4, PP5, and PP6) cloned from *P. xylostella*) were competitively inhibited by cantharidin and norcantharidin. This led to the investigators concluding that the insecticidal activity of cantharidin and its analogs is mainly due to their inhibitory action on Protein Ser/Thr Phosphatases (PSPs). PSPs are a group of enzymes that play a crucial role in regulating various cellular processes by dephosphorylating (removing phosphate groups from) specific serine and threonine residues on proteins. These enzymes are involved in controlling signal transduction pathways, cell cycle progression, and other essential cellular functions [10]. By inhibiting PSPs, cantharidin and its analogs interfere with the normal functioning of these enzymes, leading to disruption of cellular processes and eventually causing larvicidal (insect-killing) effects on the targeted insects, such as *P. xylostella* (diamondback moth) in this context [5]. This conclusion was further confirmed by another investigation which demonstrated that cantharidin inhibited the catalytic activity of Alkaline Phosphatases (ALPs) both in vivo and in vitro, highlighting its involvement in the phosphorylation process of proteins [11]. Accordingly, cantharidin exhibits substantial promise as a compelling candidate for further scientific development in the field of insecticides.

**Figure 1:**
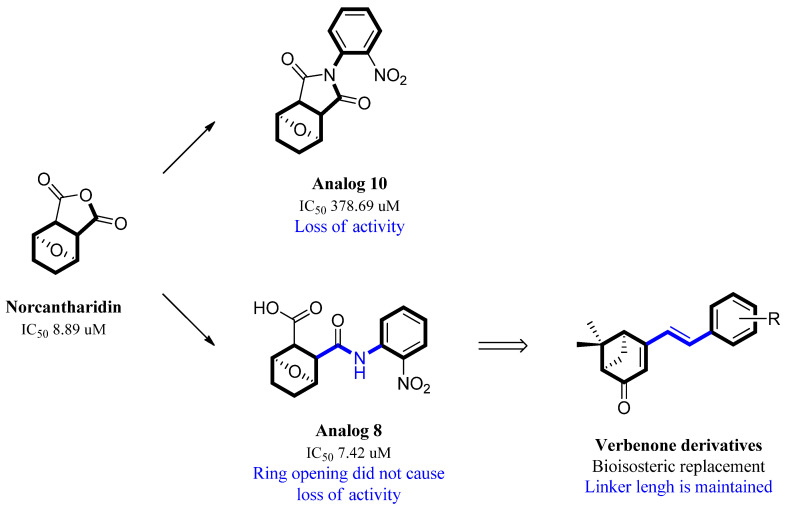
Design of the target cantharidin-based verbenone derivatives.

Previous research conducted on cantharidin has yielded valuable insights into its role as an insecticidal agent and its structure–activity relationship. Notably, these studies have demonstrated that replacing the oxygen moiety in the dihydrofuran ring with a nitrogen moiety resulted in the loss of the compound’s insecticidal activity (Figure 1) [5,7]. This emphasizes the critical importance of the oxygen atom for cantharidin’s biological activity. Interestingly, it has been observed that opening the dihydrofuran ring does not lead to a similarly significant loss of insecticidal activity. This suggests that modifications to the dihydrofuran ring can be explored without fully compromising the compound’s insecticidal properties. 

Building upon this knowledge, we aimed to develop new compounds capable of combating prevalent insecticidal resistance. This process was initiated by investigating the effect of different absolute configurations of sample cantharidin-based verbenone derivatives on the percentage of mortality observed in targeted insects. Based on these assessments, the R configuration was identified as the active configuration and was selected for further derivatization. These cantharidin-based verbenone derivatives were then synthesized and evaluated for their insecticidal effect against the diamondback moth, with the primary objective being to identify promising compounds that can effectively address insecticidal resistance. The percentage of mortality in the targeted insects served as a measure of each compound’s efficacy, enabling the identification of the most promising candidates for further investigation. Additionally, a 3D Quantitative Structure–Activity Relationship (3D QSAR) investigation was carried out to elucidate the relationship between the compounds’ activity and key structural features. Through this integrated approach, we gained valuable insights into the potential mechanisms of action of the synthesized compounds, providing a path for future development.

## 2. Results and Discussion

### 2.1. Chemistry

**Scheme 1:**
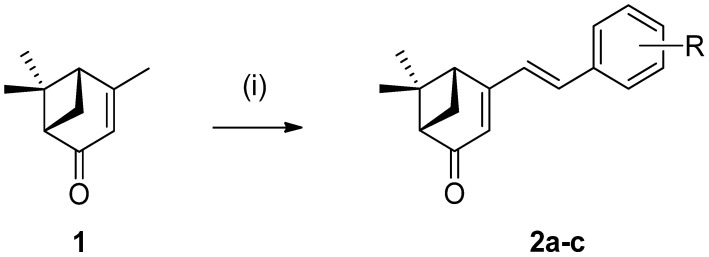
Reagents and conditions: (i) pyridinecarboxaldehydes (1.2 eq.), NaOMe (1.5 eq.), MeOH, 60 °C, 6 h.

The (*S*)-verbenone derivatives **2a–c** were readily synthesized by aldol reaction of (1*S*)-(-)-verbenone (**1**) and pyridinecarboxaldehydes using NaOMe as a base in MeOH with high yields, as shown in Scheme 1.

**Scheme 2:**
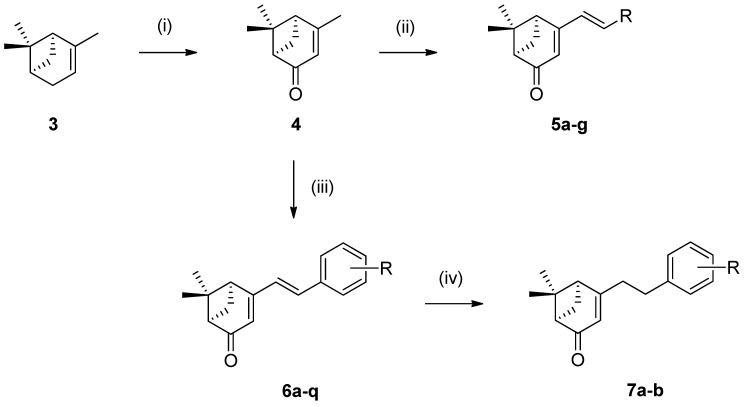
Reagents and conditions: (**i**) *N*-hydroxyphthalimide (1.1 eq.), CrO_3_ (1.0 eq.), acetone, water, rt, 20 h; (**ii**) heterocyclic aldehydes (1.5 eq.), KOH (2.0 eq.), MeOH, 60 °C, 6 h; (**iii**) substituted benzaldehydes (1.5 eq.), KOH (2.0 eq.), MeOH, 60 °C, 6 h; (**iv**) Pd on carbon (10% by wt), H_2_, ethyl acetate, rt.

All (*R*)-verbenone derivatives were also synthesized utilizing the synthetic strategy demonstrated in Scheme 2. This strategy included oxidation of (+)-α-pinene (**3**) into (*R*)-(+)-verbenone (**4**) using *N*-hydroxyphthalimide and chromium trioxide, followed by aldol condensation with various heterocyclic and aromatic aldehydes using KOH as a base in MeOH to afford products **5a**–**g** and **6a**–**q** in moderate to high yields. Compounds **7a** and **7b** were synthesized by hydrogenation of parent compounds **6a** and **6q** using Pd/C in ethyl acetate at room temperature.

### 2.2. Biological Activity

Initially, a comparative screening on the effect of absolute configuration on the larvicidal activity of the synthesized verbenone derivatives was carried out using a selection of scaffolds (Table 1). This selection of scaffolds included (*R*)-verbenone scaffolds and their corresponding (*S*)-counterparts. The larvicidal effects of verbenone derivatives on *Plutella xylostella* (L.) were measured in the 3rd instar at a concentration of 100 mg/L. The comparative study showed that (1*S*)-(-)-verbenone derivatives **2a** and **2b** containing the *ortho*- and *meta*-pyridine moieties, respectively, displayed no larvicidal activity, while (1*R*)-(+)-verbenone derivatives **5a** and **5b** of the same structure without the (*R*)- and (*S*)-verbenone configuration showed larvicidal activities of 13.3% and 46.7%, respectively, at a concentration of 100 mg/L after 4 days. The (*R*)-verbenone analogue **5c** containing the *para*-pyridine moiety had 20% higher mortality than its counterpart, **2c**. (Entries 1–6, Table 1). Subsequently, the larvicidal effects of (1*R*)-(+)-verbenone derivatives containing heterocyclic rings were investigated. They displayed poor to moderate larvicidal activity. The highest % mortality was observed with compound **5c** containing 4-pyridine ring (Entry 6, Table 1). Compounds **5b**, **5e**, and **5g** also displayed moderate larvicidal activities (Entries 5, 8, 10, Table 1). This result indicated that the (1*R*)-(+)-verbenone derivatives were clearly more pesticidal than (1*S*)-(-)-verbenone derivatives. Accordingly, only the (1*R*)-(+)-verbenone derivatives were chosen for further optimization.

Next, the replacement of the heterocyclic rings with aromatic ones was carried out to investigate their effect on larvicidal activity. Among the synthesized compounds, compounds **6a**, **6h**, **6i**, and **6q** containing phenyl ring, 2,4- and 3,4-dichlorophenyl rings and 4-ethyl-phenyl ring, respectively, displayed excellent larvicidal activity of 100% at a concentration of 100 mg/L (Entries 1, 8, 9, and 17, Table 2). Compound **6k** containing *ortho*-OCF_3_ substituted phenyl ring also showed a similarly potent mortality of 96.7% (Entry 11, Table 2). Derivatives with fluoro- or chloro-substituted phenyl rings displayed poor to excellent insecticidal activities depending upon the size and position of the halogens (Entries 2–4, 5–7, and 8–10, Table 2).

Saturation of the linker between the verbenone and phenyl ring moieties was accomplished to investigate the effect of the double bond on the activity. The saturated compounds **7a** and **7b** were synthesized, and their larvicidal effects on *P. xylostella* (L.) were compared with their unsaturated counterparts, compounds **6a** and **6q**. Saturation of the double bond with a single bond in the linker resulted in total loss of the insecticidal activity, indicating the necessity of the double bond, as shown in Table 3.

**Figure 2:**
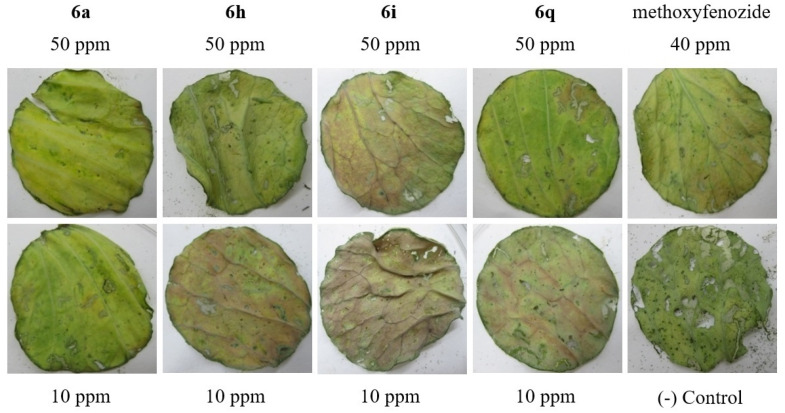
Cabbage leaves treated with compounds **6a**, **6h**, **6i**, and **6q** incubating *P. xylostella* after 4 days.

Based on the results of the structure–activity relationship study, compounds **6a**, **6h**, **6i**, and **6q** emerged as the lead compounds with promising insecticidal activity. An additional dose-dependent assay was performed by examining the effect of dose-loading the lead compounds at different intervals of time on *P. xylostella* (L.). Among the investigated compounds, **6a** showed the most promising result, with 100% mortality at a concentration of 50 mg/L (Table 4). This result was promising when compared to the commercial pesticide, methoxyfenozide, which possessed 96.7% inhibition at a concentration of 40 mg/L. It was used as the positive control. Meanwhile, compounds **6h**, **6i**, and **6q** exhibited 100% mortality at a concentration of 100 mg/L, indicating a slightly weakened activity when compared to compound 6a. All four compounds exhibited promising larvicidal activity at concentrations of 50 and 10 mg/L after 96 h. The cabbage leaves in which the 3rd instar larvae of *P. xylostella* were cultured for 4 days showed a significant difference in condition between those treated with the compounds **6a**, **6h**, **6i**, and **6q** and the negative control, as is evident from Figure 2.

Collectively, the above results can be used to identify certain patterns and insights. Particularly noteworthy is the substantial impact of the absolute configuration of the verbenone moiety on the larvicidal activity of the derivatives. The replacement of the heterocyclic rings with aromatic ones results in a significant increase in larvicidal activity. This is demonstrated by compounds **6a**, **6h**, **6i**, and **6q**, which displayed excellent larvicidal activity of 100% at a concentration of 100 mg/L. The presence of a double bond between the verbenone and phenyl ring moieties is necessary for larvicidal activity. Saturation of this double bond with a single bond in the linker moiety resulted in total loss of the insecticidal activity.

### 2.3. 3D QSAR

The accuracy and predictive capability of the 3D QSAR model heavily rely on the proper alignment of the compounds in the three-dimensional space [12]. To create the QSAR model, compounds **2a–6q** were aligned using Maestro software (ligand alignment module at default settings), with structure **6i** (exhibiting 100% mortality) chosen as the reference for displaying the QSAR models. The QSAR modeling process involves a field-based approach, which aims to establish a correlation between the ligand’s electrostatic, hydrophobic, steric fields, hydrogen bond donors, and hydrogen bond acceptors and their resulting biological activity or inactivity [13]. The interaction energy calculations for the 3D QSAR model were performed using Gaussian field analysis. To create the training and test sets, a random assignment of a 70:30% ratio was made, respectively [14]. The Partial Least Squares (PLS) method was utilized with a maximum of four PLS factors to correlate the Gaussian field descriptors as independent variables and the pIC_50_ values as dependent variables. Four features were generated from the Gaussian field analysis, each representing different properties such as lipophilic, electronegative, electropositive, steric, and hydrogen bond acceptors. These features were visualized with different colors, indicating the substituent effects on the regions and whether they enhance or decrease the compound’s biological activity [15,16,17]. The results of the most statistically significant model (model 4) are summarized in Table 5.

**Figure 3:**
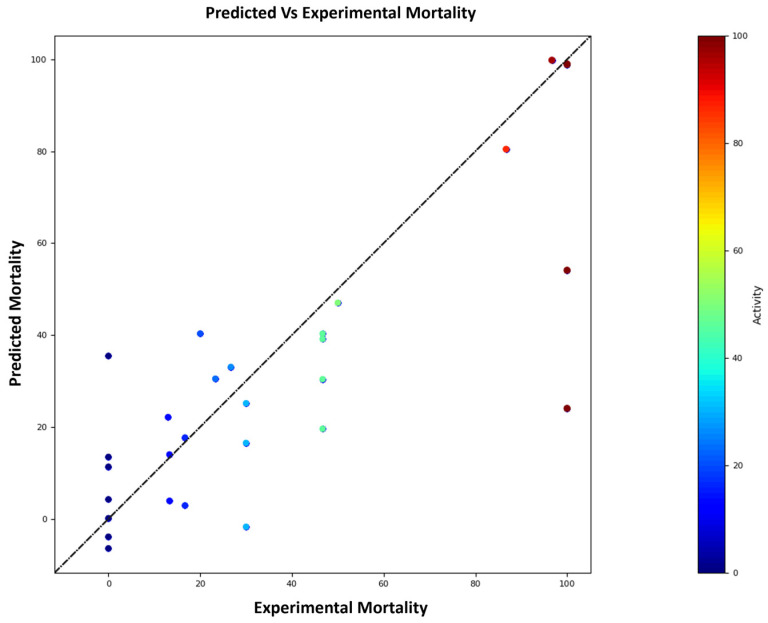
Relationship between experimental and predicted mortality (%); R^2^ = 0.94, *p* = 1.07 × 10^−9^, and MPE = −5.48%.

The model displayed an R^2^ of 0.94 (Figure 3), indicating a strong correlation between predicted and observed values. Additionally, the F-value of 65.2 and the low *p*-value (*p* = 1.07 × 10^−9^) signify the statistical significance of the model and instill a high degree of confidence in its prediction. The R^2^ scramble value of 0.78 further underscores the model’s robustness against stochastic perturbations. This value is derived through a process of randomizing actual data points and subsequently recalculating the R^2^ metric. A lower R^2^ scramble value would suggest a vulnerability to arbitrary fluctuations. However, the observed R2 scramble value of 0.78 considerably exceeds null, signifying the model’s resistance to arbitrary perturbations and implying the potential for replicable outcomes. Moreover, the QSAR model exhibited a mean prediction error (MPE) of −5.48%, indicating that the model’s predictions are very close to the actual values, with a negative sign indicating a slight underestimation. These results instill confidence in the model’s suitability for prediction and analysis of the synthesized scaffold.

**Figure 4:**
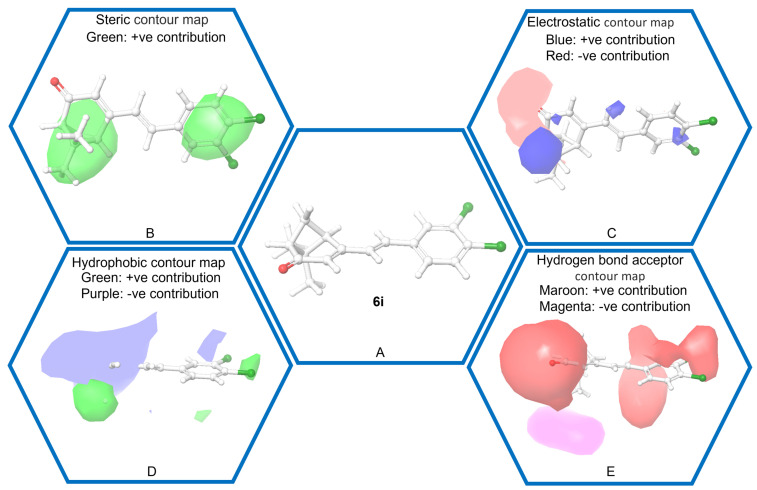
QSAR contour map. (**A**) 3D structure of the chosen representative compound (compound **6i)**, (**B**) Steric contour map (green is positive), (**C**) Electrostatic contour map (blue is positive and red is negative), (**D**) Hydrophobic contour map (green is positive and purple is negative), (**E**) H−bond acceptor map (maroon is positive and magenta is negative).

The 3D QSAR model contours, illustrated in Figure 4, provide valuable insights into the factors influencing the insecticidal activity of the studied molecules [18,19]. The contours reveal that bulky groups are well accommodated on both the verbenone and benzene rings at specific positions. Furthermore, the electrostatic contour highlights that electrostatic contributions at positions c11 of the verbenone ring and c4 of the benzene ring are associated with increased insecticidal activity. Similarly, hydrophobic substitutions at c16 and c17 of the verbenone ring, as well as c5 of the benzene ring, contribute to enhanced insecticidal activity. However, hydrophobic substitutions near the carbonyl moiety of the verbenone ring led to a reduction in insecticidal activity. Additionally, the overall structure exhibits favorable interactions with hydrogen bond acceptors at various positions, indicating its adaptability to these functional groups. These findings shed light on the specific molecular features that are critical for optimizing insecticidal potency and provide valuable guidance for designing new and effective insecticides.

## 3. Materials and Methods

### 3.1. Equipment and Materials

All the commercial chemicals were of reagent grade and used without further purification. Solvents were dried with standard procedures. The proton nuclear magnetic resonance (^1^H NMR) spectra were determined on a Varian (500 MHz) spectrometer. ^13^C NMR spectra were recorded on a Varian (126 MHz) spectrometer. Multiplicities of NMR signals were reported using different abbreviations like singlet (s), doublet (d), triplet (t), quartet (q), pentet (p), and multiplet (m). The chemical shifts were provided in parts per million (ppm) downfield with coupling constants in hertz (Hz). The spectral data for all synthesized compounds is available in the Supplementary File. The mass spectra were recorded on Jeol JMS-700 high-resolution mass spectrometer (Fast atom bombardment) (Tokyo, Japan) and Thermo Fisher Scientific LCQ FleetTM Ion Trap mass spectrometer (ESI) (Waltham, MA, USA). Melting points were measured on a Fisherbrand digital melting point apparatus. The products from all the reactions were purified by flash column chromatography using silica gel 60 (230–400 mesh Kieselgel 60). Additionally, thin-layer chromatography on 0.25 mm silica plates (E. Merck; silica gel 60 F254, Darmstadt, Germany) was used to monitor reactions. Final product purity was checked by high-pressure liquid chromatography (HPLC), performed on a Waters-2695 equipped with an ultraviolet (UV) detector set at 254 nm. The mobile phases used were (A) H_2_O containing 0.05% trifluoroacetic acid and (B) CH_3_CN. HPLC employed a YMC Hydrosphere C18 (HS-302) column (5 µm particle size, 12 nm pore size) that was 4.6 mm in diameter × 150 mm in size with a flow rate of 1.0 mL/min. Compound purity was assessed using the following method: CH_3_CN:0.05% TFA in H_2_O from 30:70 to 100:0 with a flow rate of 1.0 mL/min at λ_max_ of each compound. The purity of all evaluated compounds was >96%.

### 3.2. General Procedure for the Synthesis of Verbenone Derivatives

#### 3.2.1. (1*S*,5*R*)-6,6-dimethyl-4-((*E*)-2-(pyridin-2-yl)vinyl)bicyclo[3.1.1]hept-3-en-2-one (2a)

NaOCH_3_ (108.4 mg, 2.00 mmol) was added to a stirred solution of (1*S*)-(-)-verbenone (**1**) (200.0 mg, 1.33 mmol) and picolinaldehyde (171.6 mg, 1.60 mmol) in MeOH (7 mL). The reaction mixture was stirred at 60 °C for 6 h and then cooled to room temperature. A few drops of H_2_O were added, and it was allowed to stand at room temperature for 24 h. After removal of MeOH by evaporation in vacuo, the reaction mixture was diluted with H_2_O and extracted with ethyl acetate. The combined organic layers were washed with H_2_O and brine. It was dried over MgSO_4_ and evaporated under vacuum (55 °C) to give a crude product. Purification by flash column chromatography (silica gel, 5% ethyl acetate in hexane) afforded **2a** as a brown liquid in 77.4% yield (246.3 mg). ^1^H NMR (CDCl_3_, 500 MHz): δ 8.60 (d, *J* = 4.7 Hz, 1H), 7.67 (td, *J* = 7.7, 1.7 Hz, 1H), 7.45 (d, *J* = 15.9 Hz, 1H), 7.38 (d, *J* = 7.9 Hz, 1H), 7.18 (ddd, *J* = 7.5, 4.7, 0.9 Hz, 1H), 6.96 (d, *J* = 15.9 Hz, 1H), 6.02 (s, 1H), 3.11 (t, *J* = 5.6 Hz, 1H), 2.91 (dt, *J* = 9.6, 5.6 Hz, 1H), 2.73 (td, *J* = 5.7, 1.5 Hz, 1H), 2.11 (d, *J* = 9.5 Hz, 1H), 1.56 (s, 3H), 0.99 (s, 3H); ^13^C NMR (CDCl_3_, 126 MHz): δ 204.07, 163.65, 154.47, 150.10, 136.80, 134.04, 131.29, 124.43, 123.28, 58.42, 53.05, 44.00, 40.11, 26.86, 22.26; HRMS (FAB) [M + H]^+^ Calcd for C_16_H_18_NO: 240.1383, Found: 240.1396; HPLC purity: 98.4%, t_R_ = 4.9 min.

#### 3.2.2. (1*S*,5*R*)-6,6-dimethyl-4-((*E*)-2-(pyridin-3-yl)vinyl)bicyclo[3.1.1]hept-3-en-2-one (2b)

The procedure was similar to that of **2a** and afforded **2b** as a yellow solid in 73.6% yield. mp: 102–104 °C; ^1^H NMR (CDCl_3_, 500 MHz): δ 8.68 (d, *J* = 1.9 Hz, 1H), 8.51 (dd, *J* = 4.7, 1.3 Hz, 1H), 7.82 (dt, *J* = 8.0, 1.9 Hz, 1H), 7.28 (dd, *J* = 8.0, 4.8 Hz, 1H), 6.99 (d, *J* = 16.2 Hz, 1H), 6.88 (d, *J* = 16.2 Hz, 1H), 5.95 (s, 1H), 3.10 (td, *J* = 5.8, 1.2 Hz, 1H), 2.92 (dt, *J* = 9.4, 5.6 Hz, 1H), 2.73 (td, *J* = 5.7, 1.7 Hz, 1H), 2.10 (d, *J* = 9.5 Hz, 1H), 1.57 (s, 3H), 1.00 (s, 3H); ^13^C NMR (CDCl_3_, 126 MHz): δ 203.85, 163.41, 149.94, 149.47, 133.25, 131.85, 131.10, 129.47, 123.74, 58.35, 52.97, 43.77, 40.08, 26.85, 22.26; HRMS (FAB) [M + H]^+^ Calcd for C_16_H_18_NO: 240.1383, Found: 240.1393; HPLC purity: 98.5%, t_R_ = 3.9 min.

#### 3.2.3. (1*S*,5*R*)-6,6-dimethyl-4-((*E*)-2-(pyridin-4-yil)vinyl)bicyclo[3.1.1]hept-3-en-2-one (2c)

The procedure was similar to that of **2a** and afforded **2c** as a brown liquid in 76.8% yield. ^1^H NMR (CDCl_3_, 500 MHz): δ 8.59 (d, *J* = 5.9 Hz, 2H), 7.32 (d, *J* = 6.0 Hz, 2H), 7.10 (d, *J* = 16.2 Hz, 1H), 6.81 (d, *J* = 16.2 Hz, 1H), 5.99 (s, 1H), 3.07 (t, *J* = 5.8 Hz, 1H), 2.92 (dt, *J* = 9.5, 5.6 Hz, 1H), 2.74 (td, *J* = 5.7, 1.6 Hz, 1H), 2.10 (d, *J* = 9.5 Hz, 1H), 1.58 (s, 3H), 0.99 (s, 3H); ^13^C NMR (126 MHz, cdcl_3_) δ 203.70, 162.91, 150.55, 143.25, 132.02, 131.70, 124.75, 121.37, 58.37, 53.03, 43.82, 40.08, 26.84, 22.26; HRMS (FAB) [M + H]^+^ Calcd for C_16_H_18_NO: 240.1383, Found: 240.1390; HPLC purity: 98.1%, t_R_ = 4.3 min.

#### 3.2.4. (1*S*,5*R*)-4,6,6-trimethylbicyclo[3.1.1]hept-3-en-2-one (4) [20]

To a two-neck round-bottom flask with acetone (45 mL) and water (5 mL), (*R*)-(+)-α-pinene (**3**) (1.3 g, 0.01 mol) and *N*-hydroxyphthalimide (1.8 g, 0.01 mol) were added once. The mixture was stirred vigorously at room temperature, and then chrominum trioxide (1.0 g, 0.01 mol) was added to the mixture. After 3 h of stirring, chrominum trioxide (1.0 g, 0.01 mol) was added once again to the mixture. The reaction mixture was stirred continuously for 20 h and then worked up by evaporating the acetone in vacuo. The residue was diluted with dichloromethane and filtered. It was dried over MgSO_4_ and evaporated under vacuum (55 °C) to give a crude product. Purification by flash column chromatography (silica gel, 5% ethyl acetate in hexane) afforded **4** as a yellow liquid in 50.6% yield. (0.76 g); ^1^H NMR (CDCl_3_, 500 MHz): δ 5.71 (dd, *J* = 3.1, 1.5 Hz, 1H), 2.79 (dt, *J* = 9.2, 5.5 Hz, 1H), 2.63 (td, *J* = 6.0, 1.7 Hz, 1H), 2.44–2.37 (m, 1H), 2.07 (d, *J* = 9.2 Hz, 1H), 2.00 (d, *J* = 1.5 Hz, 3H), 1.48 (s, 3H), 1.00 (s, 3H); ^13^C NMR (CDCl_3_, 126 MHz): δ 203.7, 169.9, 121.1, 57.6, 53.9, 49.7, 40.7, 26.5, 23.4, 22.0; HRMS (FAB) [M + H]^+^ Calcd for C_10_H_15_O: 151.1118, Found: 151.1100.

#### 3.2.5. (1*S*,5*R*)-6,6-dimethyl-4-((*E*)-2-(pyridin-2-yl)vinyl)bicyclo[3.1.1]hept-3-en-2-one (5a)

To a stirred solution of (1*R*)-(+)-verbenone (**4**) (200.2 mg, 1.33 mmol) and picolinaldehyde (214.1 mg, 2.00 mmol) in MeOH (7 mL), KOH (149.6 mg, 2.66 mmol) was added. The reaction mixture was stirred at 60 °C for 6 h and then cooled to room temperature. A few drops of H_2_O were added, and it was allowed to stand at room temperature for 24 h. After removal of MeOH by evaporation in vacuo, the reaction mixture was diluted with H_2_O and extracted with ethyl acetate. The combined organic layers were washed with H_2_O and brine. It was dried over MgSO_4_ and evaporated under vacuum (55 °C) to give a crude product. Purification by flash column chromatography (silica gel, 5% ethyl acetate in hexane) afforded **5a** as yellow liquid in 47.1% yield (150.3 mg). ^1^H NMR (CDCl_3_, 500 MHz): δ 8.59 (ddd, *J* = 4.8, 1.8, 0.8 Hz, 1H), 7.66 (td, *J* = 7.7, 1.8 Hz, 1H), 7.44 (d, *J* = 15.8 Hz, 1H), 7.37 (dt, *J* = 7.9, 0.9 Hz, 1H), 7.17 (ddd, *J* = 7.6, 4.8, 1.1 Hz, 1H), 6.95 (d, *J* = 15.9 Hz, 1H), 6.00 (s, 1H), 3.10 (td, *J* = 5.9, 1.4 Hz, 1H), 2.90 (dt, *J* = 9.5, 5.6 Hz, 1H), 2.72 (td, *J* = 5.7, 1.8 Hz, 1H), 2.10 (d, *J* = 9.5 Hz, 1H), 1.55 (s, 3H), 0.98 (s, 3H); ^13^C NMR (CDCl_3_, 126 MHz): δ 204.00, 163.62, 154.41, 150.06, 136.76, 134.01, 131.22, 124.37, 123.24, 58.37, 52.99, 43.95, 40.06, 26.81, 22.22; HRMS (FAB) [M + H]^+^ Calcd for C_16_H_18_NO: 240.1383, Found: 240.1392; HPLC purity: 99.1%, t_R_ = 3.9 min.

#### 3.2.6. (1*S*,5*R*)-6,6-dimethyl-4-((*E*)-2-(pyridin-3-yl)vinyl)bicyclo[3.1.1]hept-3-en-2-one (5b)

The procedure was similar to that of **5a** and afforded **5b** as a yellow solid in 86.9% yield. mp: 86–89 °C; ^1^H NMR (CDCl_3_, 500 MHz): δ 8.69 (d, *J* = 1.9 Hz, 1H), 8.52 (dd, *J* = 4.7, 1.4 Hz, 1H), 7.84 (dt, *J* = 8.0, 1.9 Hz, 1H), 7.30 (dd, *J* = 8.0, 4.8 Hz, 1H), 6.95 (dd, *J* = 55.9, 16.2 Hz, 2H), 5.95 (s, 1H), 3.11 (td, *J* = 5.8, 1.3 Hz, 1H), 2.93 (dt, *J* = 9.5, 5.6 Hz, 1H), 2.73 (td, *J* = 5.7, 1.7 Hz, 1H), 2.11 (d, *J* = 9.5 Hz, 1H), 1.58 (s, 3H), 1.01 (s, 3H); ^13^C NMR (CDCl_3_, 126 MHz): δ 203.74, 163.36, 149.83, 149.36, 133.21, 131.78, 131.05, 129.37, 123.70, 123.60, 58.26, 52.87, 43.68, 40.00, 26.76, 22.17; HRMS (FAB) [M + H]^+^ Calcd for C_16_H_18_NO: 240.1383, Found: 240.1393; HPLC purity: 98.2%, t_R_ = 3.4 min.

#### 3.2.7. (1*S*,5*R*)-6,6-dimethyl-4-((*E*)-2-(pyridin-4-yl)vinyl)bicyclo[3.1.1]hept-3-en-2-one (5c)

The procedure was similar to that of **5a** and afforded **5c** as a brown liquid in 23.6% yield. ^1^H NMR (CDCl_3_, 500 MHz): δ 8.60 (d, *J* = 5.8 Hz, 2H), 7.33 (dd, *J* = 4.6, 1.6 Hz, 2H), 7.11 (d, *J* = 16.1 Hz, 1H), 6.82 (d, *J* = 16.2 Hz, 1H), 6.00 (s, 1H), 3.08 (td, *J* = 5.8, 1.4 Hz, 1H), 2.93 (dt, *J* = 9.5, 5.6 Hz, 1H), 2.75 (td, *J* = 5.7, 1.8 Hz, 1H), 2.11 (d, *J* = 9.5 Hz, 1H), 1.58 (s, 3H), 1.00 (s, 3H); ^13^C NMR (CDCl_3_, 126 MHz): δ 203.73, 162.93, 150.54, 143.31, 132.03, 131.74, 124.80, 121.38, 58.39, 53.06, 43.85, 40.11, 26.85, 22.27; HRMS (FAB) [M + H]^+^ Calcd for C_16_H_18_NO: 240.1383, Found: 240.1392; HPLC purity: 98.6%, t_R_ = 3.5 min.

#### 3.2.8. (1*S*,5*R*)-6,6-dimethyl-4-((*E*)-2-(thiophen-2-yl)vinyl)bicyclo[3.1.1]hept-3-en-2-one (5d)

The procedure was similar to that of **5a** and afforded **5d** as a brown liquid in 66.5% yield. ^1^H NMR (CDCl_3_, 500 MHz): δ 7.28 (d, *J* = 5.0 Hz, 1H), 7.13 (d, *J* = 3.6 Hz, 1H), 7.04 (d, *J* = 15.9 Hz, 1H), 7.00 (dd, *J* = 5.1, 3.6 Hz, 1H), 6.72 (d, *J* = 15.9 Hz, 1H), 5.86 (s, 1H), 3.02 (td, *J* = 5.9, 1.2 Hz, 1H), 2.87 (dt, *J* = 9.4, 5.6 Hz, 1H), 2.69 (td, *J* = 5.7, 1.7 Hz, 1H), 2.07 (d, *J* = 9.4 Hz, 1H), 1.54 (s, 3H), 0.98 (s, 3H); ^13^C NMR (CDCl_3_, 126 MHz): δ 203.86, 163.85, 141.76, 128.72, 128.06, 127.85, 127.04, 126.80, 122.18, 58.22, 52.74, 43.80, 39.95, 26.80, 22.20; HRMS (FAB) [M + H]^+^ Calcd for C_15_H_17_OS: 245.0995, Found: 245.1004; HPLC purity: 97.7%, t_R_ = 6.2 min.

#### 3.2.9. (1*S*,5*R*)-6,6-dimethyl-4-((*E*)-2-(3-methylthiophen-2-yl)vinyl)bicyclo[3.1.1]hept-3-en-2-one (5e)

The procedure was similar to that of **5a** and afforded **5e** as a brown liquid in 64.0% yield. ^1^H NMR (CDCl_3_, 500 MHz): δ 7.17 (d, *J* = 5.1 Hz, 1H), 7.05 (d, *J* = 15.8 Hz, 1H), 6.81 (dd, *J* = 5.1, 1.6 Hz, 1H), 6.65 (d, *J* = 15.8 Hz, 1H), 5.84 (s, 1H), 3.05 (t, *J* = 5.8 Hz, 1H), 2.91–2.85 (m, 1H), 2.68 (td, *J* = 5.7, 1.7 Hz, 1H), 2.29 (s, 3H), 2.08 (dd, *J* = 8.9, 2.1 Hz, 1H), 1.55 (s, 3H), 0.99 (s, 3H); ^13^C NMR (CDCl_3_, 126 MHz): δ 203.74, 164.08, 138.70, 135.63, 131.09, 126.15, 125.96, 125.61, 121.58, 58.11, 52.61, 43.81, 39.88, 26.75, 22.11, 14.11; HRMS (FAB) [M + H]^+^ Calcd for C_16_H_19_OS: 259.1151, Found: 259.1159; HPLC purity: 96.2%, t_R_ = 7.4 min.

#### 3.2.10. (1*S*,5*R*)-6,6-dimethyl-4-((*E*)-2-(4-methylthiophen-2-yl)vinyl)bicyclo[3.1.1]hept-3-en-2-one (5f)

The procedure was similar to that of **5a** and afforded **5f** as a brown solid in 70.6% yield. mp: 69–71 °C; ^1^H NMR (CDCl_3_, 500 MHz): δ 6.98 (d, *J* = 15.9 Hz, 1H), 6.96 (d, *J* = 0.7 Hz, 1H), 6.89 (s, 1H), 6.71 (d, *J* = 15.8 Hz, 1H), 5.87 (s, 1H), 3.02 (td, *J* = 5.9, 1.4 Hz, 1H), 2.89 (dt, *J* = 9.4, 5.6 Hz, 1H), 2.71 (td, *J* = 5.7, 1.8 Hz, 1H), 2.23 (s, 3H), 2.09 (d, *J* = 9.4 Hz, 1H), 1.56 (s, 3H), 1.00 (s, 3H); ^13^C NMR (CDCl_3_, 126 MHz): δ 204.00, 164.00, 141.54, 138.79, 130.92, 128.03, 126.53, 122.72, 122.12, 58.31, 52.83, 43.92, 40.03, 26.89, 22.28, 15.70; HRMS (FAB) [M + H]^+^ Calcd for C_16_H_19_OS: 259.1151, Found: 259.1160; HPLC purity: 97.8%, t_R_ = 8.1 min.

#### 3.2.11. (1*S*,5*R*)-6,6-dimethyl-4-((*E*)-2-(5-methylthiophen-2-yl)vinyl)bicyclo[3.1.1]hept-3-en-2-one (5g)

The procedure was similar to that of **5a** and afforded **5g** as a brown liquid in 53.0% yield. ^1^H NMR (CDCl_3_, 500 MHz): δ 7.16 (d, *J* = 5.1 Hz, 1H), 7.06 (d, *J* = 15.7 Hz, 1H), 6.81 (d, *J* = 5.1 Hz, 1H), 6.65 (d, *J* = 15.7 Hz, 1H), 5.83 (s, 1H), 3.05 (td, *J* = 5.9, 1.3 Hz, 1H), 2.88 (dt, *J* = 9.3, 5.6 Hz, 1H), 2.68 (td, *J* = 5.7, 1.8 Hz, 1H), 2.28 (s, 3H), 2.06 (d, *J* = 9.3 Hz, 1H), 1.55 (s, 3H), 0.99 (s, 3H); ^13^C NMR (CDCl_3_, 126 MHz): δ 203.47, 163.93, 138.59, 135.51, 130.98, 126.05, 125.81, 125.50, 121.44, 57.98, 52.43, 43.66, 39.74, 26.63, 22.00, 14.00; HRMS (FAB) [M + H]^+^ Calcd for C_16_H_19_OS: 259.1151, Found: 259.1162; HPLC purity: 97.1%, t_R_ = 8.3 min.

#### 3.2.12. (1*S*,5*R*)-6,6-dimethyl-4-((*E*)-styryl)bicyclo[3.1.1]hept-3-en-2-one (6a)

The procedure was similar to that of **5a** and afforded **6a** as a brown liquid in 87.0% yield. ^1^H NMR (CDCl_3_, 500 MHz): δ 7.52–7.48 (m, 2H), 7.39–7.35 (m, 2H), 7.34–7.30 (m, 1H), 6.94 (q, *J* = 16.2 Hz, 2H), 5.93 (s, 1H), 3.12 (t, *J* = 5.8 Hz, 1H), 2.92 (dt, *J* = 9.5, 5.6 Hz, 1H), 2.73 (td, *J* = 5.7, 1.5 Hz, 1H), 2.12 (d, *J* = 9.4 Hz, 1H), 1.58 (s, 3H), 1.01 (s, 3H); ^13^C NMR (CDCl_3_, 126 MHz): δ 204.36, 164.41, 136.04, 135.12, 129.28, 128.99, 127.47, 122.76, 58.29, 53.03, 43.76, 40.16, 26.88, 22.27; HRMS (FAB) [M + H]^+^ Calcd for C_17_H_19_O: 239.1431, Found: 239.1440; HPLC purity: 99.8%, t_R_ = 6.8 min.

#### 3.2.13. (1*S*,5*R*)-4-(2-fluorostyryl)-6,6-dimethylbicyclo[3.1.1]hept-3-en-2-one (6b)

The procedure was similar to that of **5a** and afforded **6b** as a yellow solid in 78.8% yield. mp: 65–67 °C; ^1^H NMR (CDCl_3_, 500 MHz): δ 7.58 (t, *J* = 7.2 Hz, 1H), 7.32–7.21 (m, 1H), 7.18–6.94 (m, 4H), 5.93 (s, 1H), 3.18–3.03 (m, 1H), 2.96–2.82 (m, 1H), 2.76–2.65 (m, 1H), 2.09 (d, *J* = 9.4 Hz, 1H), 1.56 (s, 3H), 1.00 (s, 3H); ^13^C NMR (CDCl_3_, 126 MHz): δ 203.92, 164.00, 161.66, 130.37, 129.22, 127.38, 126.79, 124.37, 123.81, 123.19, 116.00, 58.12, 52.78, 43.35, 39.93, 26.65, 22.05; HRMS (FAB) [M + H]^+^ Calcd for C_17_H_18_FO: 257.1336, Found: 257.1344; HPLC purity: 99.4%, t_R_ = 7.3 min.

#### 3.2.14. (1*S*,5*R*)-4-(3-fluorostyryl)-6,6-dimethylbicyclo[3.1.1]hept-3-en-2-one (6c)

The procedure was similar to that of **5a** and afforded **6c** as a yellow solid in 93.9% yield. mp: 58–60 °C; ^1^H NMR (CDCl_3_, 500 MHz): δ 7.47–7.12 (m, 3H), 7.07–6.79 (m, 3H), 5.93 (s, 1H), 3.09 (s, 1H), 3.00–2.84 (m, 1H), 2.72 (s, 1H), 2.10 (d, *J* = 9.3 Hz, 1H), 1.57 (s, 3H), 1.00 (s, 3H); ^13^C NMR (CDCl_3_, 126 MHz): δ 203.94, 163.68, 162.05, 138.30, 133.56, 130.38, 128.60, 123.30, 115.80, 113.64, 58.16, 52.87, 43.58, 39.98, 26.71, 22.12; HRMS (FAB) [M + H]^+^ Calcd for C_17_H_18_FO: 257.1336, Found: 257.1348; HPLC purity: >99.9%, t_R_ = 6.9 min.

#### 3.2.15. (1*S*,5*R*)-4-(4-fluorostyryl)-6,6-dimethylbicyclo[3.1.1]hept-3-en-2-one (6d)

The procedure was similar to that of **5a** and afforded **6d** as a yellow liquid in 65.4% yield. ^1^H NMR (CDCl_3_, 500 MHz): δ 7.54–7.38 (m, 2H), 7.10–6.97 (m, 2H), 6.92–6.79 (m, 2H), 5.91 (s, 1H), 3.10 (t, *J* = 5.6 Hz, 1H), 2.90 (dt, *J* = 9.4, 5.6 Hz, 1H), 2.75–2.65 (m, 1H), 2.09 (d, *J* = 9.4 Hz, 1H), 1.56 (s, 3H), 1.00 (s, 3H); ^13^C NMR (CDCl_3_, 126 MHz): δ 203.88, 163.99, 161.96, 133.57, 132.15, 129.01, 128.95, 127.00, 122.49, 115.90, 115.73, 58.04, 52.71, 43.47, 39.88, 26.61, 22.02; HRMS (FAB) [M + H]^+^ Calcd for C_17_H_18_FO: 257.1336, Found: 257.1349; HPLC purity: 99.8%, t_R_ = 6.7 min.

#### 3.2.16. (1*S*,5*R*)-4-(2,4-difluorostyryl)-6,6-dimethylbicyclo[3.1.1]hept-3-en-2-one (6e)

The procedure was similar to that of **5a** and afforded **6e** as a yellow liquid in 32.1% yield. ^1^H NMR (CDCl_3_, 500 MHz): δ 7.55 (td, *J* = 8.6, 6.4 Hz, 1H), 6.95 (q, *J* = 16.4 Hz, 2H), 6.90–6.84 (m, 1H), 6.81 (ddd, *J* = 11.1, 8.7, 2.5 Hz, 1H), 5.91 (s, 1H), 3.08 (td, *J* = 5.8, 1.4 Hz, 1H), 2.90 (dt, *J* = 9.4, 5.6 Hz, 1H), 2.71 (td, *J* = 5.7, 1.8 Hz, 1H), 2.08 (d, *J* = 9.4 Hz, 1H), 1.56 (s, 3H), 0.99 (s, 3H); ^13^C NMR (CDCl_3_, 126 MHz): δ 203.88, 163.88, 162.01, 159.89, 129.15, 128.58, 125.89, 123.37, 120.57, 112.00, 104.44, 58.32, 52.88, 43.67, 40.05, 26.80, 22.19; HRMS (FAB) [M + H]^+^ Calcd for C_17_H_17_F_2_O: 275.1242, Found: 275.1268; HPLC purity: 97.6%, t_R_ = 7.9 min.

#### 3.2.17. (1*S*,5*R*)-4-(3,4-difluorostyryl)-6,6-dimethylbicyclo[3.1.1]hept-3-en-2-one (6f)

The procedure was similar to that of **5a** and afforded **6f** as a yellow solid in 26.5% yield. mp: 81–83 °C; ^1^H NMR (CDCl_3_, 500 MHz): δ 7.23 (dd, *J* = 12.3, 2.1 Hz, 1H), 7.17 (d, *J* = 8.6 Hz, 1H), 6.92 (t, *J* = 8.5 Hz, 1H), 6.86–6.69 (m, 2H), 5.87 (s, 1H), 3.06 (t, *J* = 5.4 Hz, 1H), 2.88 (dt, *J* = 9.4, 5.6 Hz, 1H), 2.69 (td, *J* = 5.7, 1.6 Hz, 1H), 2.07 (d, *J* = 9.4 Hz, 1H), 1.55 (s, 3H), 0.98 (s, 3H); ^13^C NMR (CDCl_3_, 126 MHz): δ 203.99, 164.14, 151.58, 148.53, 133.59, 129.53, 126.58, 124.26, 122.36, 114.28, 113.44, 58.26, 52.81, 43.86, 40.02, 26.80, 22.20; MS (ESI) [M + H]^+^ Calcd for C_17_H_17_F_2_O: 275.1242, Found: 275.06; HPLC purity: 97.4%, t_R_ = 6.2 min.

#### 3.2.18. (1*S*,5*R*)-4-(3,5-difluorostyryl)-6,6-dimethylbicyclo[3.1.1]hept-3-en-2-one (6g)

The procedure was similar to that of **5a** and afforded **6g** as a yellow solid in 51.3% yield. mp: 106–108 °C; ^1^H NMR (CDCl_3_, 500 MHz): δ 7.09–6.89 (m, 3H), 6.87–6.66 (m, 2H), 5.96 (s, 1H), 3.14–3.01 (m, 1H), 2.99–2.85 (m, 1H), 2.80–2.66 (m, 1H), 2.10 (d, *J* = 9.2 Hz, 1H), 1.58 (s, 3H), 1.00 (s, 3H); ^13^C NMR (CDCl_3_, 126 MHz): δ 203.80, 164.24, 163.14, 162.27, 139.34, 132.36, 129.85, 124.04, 110.00, 109.79, 104.13, 58.18, 52.93, 43.59, 39.98, 26.69, 22.11; HRMS (FAB) [M + H]^+^ Calcd for C_17_H_17_F_2_O: 275.1242, Found: 275.1243; HPLC purity: 99.4%, t_R_ = 7.7 min.

#### 3.2.19. (1*S*,5*R*)-4-(2,4-dichlorostyryl)-6,6-dimethylbicyclo[3.1.1]hept-3-en-2-one (6h)

The procedure was similar to that of **5a** and afforded **6h** as a yellow solid in 56.5% yield. mp: 117–119 °C; ^1^H NMR (CDCl_3_, 500 MHz): δ 7.59 (d, *J* = 8.2 Hz, 1H), 7.38 (s, 1H), 7.31–7.14 (m, 2H), 6.90 (d, *J* = 16.1 Hz, 1H), 5.95 (s, 1H), 3.19–3.04 (m, 1H), 3.01–2.84 (m, 1H), 2.80–2.64 (m, 1H), 2.11 (d, *J* = 9.2 Hz, 1H), 1.58 (s, 3H), 1.01 (s, 3H); ^13^C NMR (CDCl_3_, 126 MHz): δ 203.93, 163.61, 134.99, 134.65, 132.63, 130.13, 129.80, 129.28, 127.62, 127.56, 123.92, 58.21, 52.98, 43.67, 40.08, 26.79, 22.17; HRMS (FAB) [M + H]^+^ Calcd for C_17_H_17_Cl_2_O: 307.0651, Found: 307.0665; HPLC purity: 99.8%, t_R_ = 16.8 min.

#### 3.2.20. (1*S*,5*R*)-4-(3,4-dichlorostyryl)-6,6-dimethylbicyclo[3.1.1]hept-3-en-2-one (6i)

The procedure was similar to that of **5a** and afforded **6i** as a yellow solid in 31.3% yield. mp: 94–96 °C; ^1^H NMR (CDCl_3_, 500 MHz): δ 7.56 (s, 1H), 7.41 (d, *J* = 8.2 Hz, 1H), 7.31 (d, *J* = 7.5 Hz, 1H), 6.85 (dd, *J* = 64.7, 16.1 Hz, 2H), 5.94 (s, 1H), 3.12–3.01 (m, 1H), 2.97–2.84 (m, 1H), 2.78–2.66 (m, 1H), 2.10 (d, *J* = 9.4 Hz, 1H), 1.57 (s, 3H), 0.99 (s, 3H); ^13^C NMR (CDCl_3_, 126 MHz): δ 203.98, 163.43, 136.15, 133.15, 132.81, 132.23, 130.85, 129.18, 128.97, 126.36, 123.74, 58.25, 53.00, 43.66, 40.08, 26.81, 22.22; HRMS (FAB) [M + H]^+^ Calcd for C_17_H_17_Cl_2_O: 307.0651, Found: 307.0667; HPLC purity: 99.5%, t_R_ = 13.1 min.

#### 3.2.21. (1*S*,5*R*)-4-(3,5-dichlorostyryl)-6,6-dimethylbicyclo[3.1.1]hept-3-en-2-one (6j)

The procedure was similar to that of **5a** and afforded **6j** as a yellow solid in 51.4% yield. mp: 122–124 °C; ^1^H NMR (CDCl_3_, 500 MHz): δ 7.33 (d, *J* = 1.8 Hz, 2H), 7.24 (t, *J* = 1.8 Hz, 1H), 6.84 (dd, *J* = 86.8, 16.1 Hz, 2H), 5.96 (s, 1H), 3.05 (td, *J* = 5.8, 1.1 Hz, 1H), 2.92 (dt, *J* = 9.5, 5.6 Hz, 1H), 2.73 (td, *J* = 5.7, 1.7 Hz, 1H), 2.10 (d, *J* = 9.5 Hz, 1H), 1.57 (s, 3H), 0.99 (s, 3H); ^13^C NMR (CDCl_3_, 126 MHz): δ 03.72, 163.05, 138.97, 135.38, 131.80, 129.95, 128.52, 125.47, 124.08, 58.16, 52.91, 43.55, 39.96, 26.71, 22.13; HRMS (FAB) [M + H]^+^ Calcd for C_17_H_17_Cl_2_O: 307.0651, Found: 307.0662; HPLC purity: 99.9%, t_R_ = 15.5 min.

#### 3.2.22. (1*S*,5*R*)-6,6-dimethyl-4-(2-(trifluoromethoxy)styryl)bicyclo[3.1.1]hept-3-en-2-one (6k)

The procedure was similar to that of **5a** and afforded **6k** as a yellow liquid in 56.3% yield. ^1^H NMR (CDCl_3_, 500 MHz): δ 7.70 (dd, *J* = 7.4, 2.1 Hz, 1H), 7.30 (pd, *J* = 7.3, 1.7 Hz, 2H), 7.26–7.21 (m, 1H), 7.07 (dd, *J* = 85.2, 16.3 Hz, 2H), 5.96 (s, 1H), 3.09 (t, *J* = 5.8 Hz, 1H), 2.90 (dt, *J* = 9.5, 5.6 Hz, 1H), 2.72 (td, *J* = 5.7, 1.5 Hz, 1H), 2.08 (d, *J* = 9.4 Hz, 1H), 1.56 (s, 3H), 0.99 (s, 3H); ^13^C NMR (CDCl_3_, 126 MHz): δ 203.21, 163.42, 146.76, 129.82, 129.77, 129.21, 127.00, 126.88, 126.78, 123.39, 121.14, 121.13, 58.00, 52.54, 43.53, 39.58, 26.38, 21.73; HRMS (FAB) [M + H]^+^ Calcd for C_18_H_18_F_3_O_2_: 323.1254, Found: 323.1276; HPLC purity: 98.6%, t_R_ = 10.9 min.

#### 3.2.23. (1*S*,5*R*)-6,6-dimethyl-4-(3-(trifluoromethoxy)styryl)bicyclo[3.1.1]hept-3-en-2-one (6l)

The procedure was similar to that of **5a** and afforded **6l** as a yellow liquid in 59.5% yield. ^1^H NMR (CDCl_3_, 500 MHz): δ 7.42 (dt, *J* = 7.7, 1.1 Hz, 1H), 7.37 (t, *J* = 7.9 Hz, 1H), 7.32 (s, 1H), 7.17–7.11 (m, 1H), 6.92 (dd, *J* = 37.5, 16.2 Hz, 2H), 5.95 (s, 1H), 3.09 (td, *J* = 5.8, 1.3 Hz, 1H), 2.91 (dt, *J* = 9.4, 5.6 Hz, 1H), 2.73 (td, *J* = 5.7, 1.7 Hz, 1H), 2.10 (d, *J* = 9.4 Hz, 1H), 1.57 (s, 3H), 1.00 (s, 3H); ^13^C NMR (CDCl_3_, 126 MHz): δ 203.81, 163.56, 149.75, 138.23, 133.19, 130.23, 129.04, 125.63, 123.53, 121.17, 119.52, 58.27, 52.87, 43.78, 39.98, 26.69, 22.11; MS (ESI) [M + H]^+^ Calcd for C_18_H_18_F_3_O_2_: 323.1254, Found: 323.06; HPLC purity: 99.3%, t_R_ = 10.8 min.

#### 3.2.24. (1*S*,5*R*)-6,6-dimethyl-4-(4-(trifluoromethoxy)styryl)bicyclo[3.1.1]hept-3-en-2-one (6m)

The procedure was similar to that of **5a** and afforded **6m** as a yellow liquid in 43.1% yield. ^1^H NMR (CDCl_3_, 500 MHz): δ 7.51 (d, *J* = 8.5 Hz, 2H), 7.19 (d, *J* = 8.4 Hz, 2H), 6.98–6.84 (m, 2H), 5.93 (s, 1H), 3.09 (t, *J* = 5.7 Hz, 1H), 2.97–2.86 (m, 1H), 2.72 (t, *J* = 5.6 Hz, 1H), 2.10 (d, *J* = 9.4 Hz, 1H), 1.57 (s, 3H), 1.00 (s, 3H); ^13^C NMR (CDCl_3_, 126 MHz): δ 203.81, 163.75, 149.52, 134.80, 133.17, 128.68, 128.39, 123.20, 121.21, 58.29, 52.85, 43.81, 40.00, 26.74, 22.15; MS (ESI) [M + H]^+^ Calcd for C_18_H_18_F_3_O_2_: 323.1254, Found: 323.02; HPLC purity: 99.8%, t_R_ = 10.8 min.

#### 3.2.25. (1*S*,5*R*)-6,6-dimethyl-4-(2-methylstyryl)bicyclo[3.1.1]hept-3-en-2-one (6n)

The procedure was similar to that of **5a** and afforded **6n** as a yellow liquid in 55.1% yield. ^1^H NMR (CDCl_3_, 500 MHz): δ 7.60–7.51 (m, 1H), 7.25–7.10 (m, 4H), 6.85 (d, *J* = 16.0 Hz, 1H), 5.91 (s, 1H), 3.10 (t, *J* = 5.7 Hz, 1H), 2.91 (dt, *J* = 9.4, 5.6 Hz, 1H), 2.75–2.67 (m, 1H), 2.38 (s, 3H), 2.12 (d, *J* = 9.4 Hz, 1H), 1.57 (s, 3H), 1.01 (s, 3H); ^13^C NMR (CDCl_3_, 126 MHz): δ 204.15, 164.53, 136.61, 134.85, 132.42, 130.70, 128.97, 128.50, 126.40, 125.73, 122.55, 58.17, 52.92, 43.82, 40.07, 26.82, 22.15, 19.85; HRMS (FAB) [M + H]^+^ Calcd for C_18_H_21_O: 253.1587, Found: 253.1593; HPLC purity: 99.3%, t_R_ = 8.3 min.

#### 3.2.26. (1*S*,5*R*)-6,6-dimethyl-4-(3-methylstyryl)bicyclo[3.1.1]hept-3-en-2-one (6o)

The procedure was similar to that of **5a** and afforded **6o** as a yellow liquid in 61.8% yield. ^1^H NMR (CDCl_3_, 500 MHz): δ 7.37–7.19 (m, 3H), 7.12 (d, *J* = 7.3 Hz, 1H), 6.91 (q, *J* = 16.2 Hz, 2H), 5.91 (s, 1H), 3.11 (t, *J* = 5.8 Hz, 1H), 2.90 (dt, *J* = 9.4, 5.6 Hz, 1H), 2.72 (td, *J* = 5.8, 1.7 Hz, 1H), 2.35 (s, 3H), 2.10 (d, *J* = 9.4 Hz, 1H), 1.56 (s, 3H), 1.00 (s, 3H); ^13^C NMR (CDCl_3_, 126 MHz): δ 204.20, 164.41, 138.46, 135.87, 135.22, 130.04, 128.77, 128.00, 127.14, 124.66, 122.47, 58.18, 52.87, 43.64, 40.02, 26.78, 22.16, 21.40; HRMS (FAB) [M + H]^+^ Calcd for C_18_H_21_O: 253.1587, Found: 253.1598; HPLC purity: 98.6%, t_R_ = 8.9 min.

#### 3.2.27. (1*S*,5*R*)-6,6-dimethyl-4-(4-methylstyryl)bicyclo[3.1.1]hept-3-en-2-one (6p)

The procedure was similar to that of **5a** and afforded **6p** as a yellow solid in 89.4% yield. mp: 101–103 °C; ^1^H NMR (CDCl_3_, 500 MHz): δ 7.38 (d, *J* = 8.1 Hz, 2H), 7.16 (d, *J* = 7.8 Hz, 2H), 6.89 (s, 2H), 5.89 (s, 1H), 3.10 (t, *J* = 5.8 Hz, 1H), 2.89 (dt, *J* = 9.4, 5.6 Hz, 1H), 2.71 (td, *J* = 5.8, 1.7 Hz, 1H), 2.34 (s, 3H), 2.09 (d, *J* = 9.4 Hz, 1H), 1.56 (s, 3H), 1.00 (s, 3H); ^13^C NMR (CDCl_3_, 126 MHz): δ 204.19, 164.52, 139.40, 135.07, 133.19, 129.62, 127.34, 126.35, 122.16, 58.16, 52.81, 43.63, 40.02, 26.77, 22.16, 21.43; HRMS (FAB) [M + H]^+^ Calcd for C_18_H_21_O: 253.1587, Found: 253.1597; HPLC purity: 99.9%, t_R_ = 8.8 min.

#### 3.2.28. (1*S*,5*R*)-4-(4-ethylstyryl)-6,6-dimethylbicyclo[3.1.1]hept-3-en-2-one (6q)

The procedure was similar to that of **5a** and afforded **6q** as a yellow solid in 86.9% yield. mp: 89–91 °C; ^1^H NMR (CDCl_3_, 500 MHz): δ 7.39 (d, *J* = 8.1 Hz, 2H), 7.16 (d, *J* = 8.0 Hz, 2H), 6.89 (s, 2H), 5.88 (s, 1H), 3.15–3.04 (m, 1H), 2.87 (dt, *J* = 9.4, 5.6 Hz, 1H), 2.74–2.66 (m, 1H), 2.64–2.54 (m, 2H), 2.07 (d, *J* = 9.3 Hz, 1H), 1.54 (s, 3H), 1.20 (t, *J* = 7.6 Hz, 3H), 0.98 (s, 3H); ^13^C NMR (CDCl_3_, 126 MHz): δ 203.84, 164.32, 145.53, 134.93, 133.28, 128.25, 127.29, 126.21, 121.97, 57.98, 52.58, 43.43, 39.80, 28.57, 26.59, 21.98, 15.29; HRMS (FAB) [M + H]^+^ Calcd for C_19_H_23_O: 267.1744, Found: 267.1758; HPLC purity: 99.8%, t_R_ = 11.4 min.

#### 3.2.29. (1*S*,5*R*)-6,6-dimethyl-4-phenethylbicyclo[3.1.1]hept-3-en-2-one (7a)

To a stirred solution of **6a** (220.1 mg, 0.92 mmol) in ethyl acetate (5 mL), 10% Pd/C (97.2 mg) was added. The reaction mixture was stirred under H_2_ (1 atm, balloon) for 6 h. After completion of the reaction, the catalyst was removed by filtration. The filtrate was concentrated in vacuo and diluted with H_2_O, then extracted with ethyl acetate. The combined organic layers were washed with H_2_O and brine. It was dried over MgSO_4_ and evaporated under vacuum (55 °C) to give a crude product. Purification by flash column chromatography (silica gel, 14% ethyl acetate in hexane) afforded **7a** as colorless liquid in 37.1% yield (82.0 mg). ^1^H NMR (CDCl_3_, 500 MHz): δ 7.31–7.26 (m, 2H), 7.22–7.16 (m, 3H), 5.75 (p, *J* = 1.5 Hz, 1H), 2.83–2.77 (m, 3H), 2.67–2.63 (m, 1H), 2.62–2.56 (m, 2H), 2.51–2.47 (m, 1H), 2.05 (d, *J* = 9.2 Hz, 1H), 1.50 (s, 3H), 1.00 (s, 3H); ^13^C NMR (CDCl_3_, 126 MHz): δ 203.97, 172.41, 140.72, 128.60, 128.29, 126.34, 120.52, 57.97, 54.05, 48.89, 41.08, 38.67, 32.78, 26.69, 22.42; HRMS (FAB) [M + H]^+^ Calcd for C_17_H_21_O: 241.1587, Found: 241.1593; HPLC purity: 99.0%, t_R_ = 7.2 min.

#### 3.2.30. (1*S*,5*R*)-4-(4-ethylphenethyl)-6,6-dimethylbicyclo[3.1.1]hept-3-en-2-one (7b)

The procedure was similar to that of **7a** and afforded **7b** from **30** as a colorless liquid in 51.9% yield. ^1^H NMR (CDCl_3_, 500 MHz): δ 7.11 (q, *J* = 8.1 Hz, 4H), 5.75 (s, 1H), 2.79 (ddd, *J* = 25.7, 13.3, 6.7 Hz, 3H), 2.68–2.53 (m, 5H), 2.50 (t, *J* = 5.7 Hz, 1H), 2.05 (d, *J* = 9.1 Hz, 1H), 1.50 (s, 3H), 1.23 (t, *J* = 7.6 Hz, 3H), 1.00 (s, 3H); ^13^C NMR (CDCl_3_, 126 MHz): δ 203.99, 172.57, 142.24, 137.87, 128.20, 128.05, 120.45, 57.96, 54.02, 48.88, 41.07, 38.78, 32.35, 28.49, 26.68, 22.41, 15.67; HRMS (FAB) [M + H]^+^ Calcd for C_19_H_25_O: 269.1900, Found: 269.1916; HPLC purity: 99.4%, t_R_ = 12.5 min.

### 3.3. Evaluation of Insecticidal Activity against P. xylostella

The larvicidal activity against *P. xylostella* was evaluated by the leaf dipping assay [21,22] and the following procedures. The stock solutions of each verbenone derivative were prepared immediately before use at a concentration of 500 mg/L. They were prepared by completely dissolving 50 mg of each verbenone derivative in 5 mL of acetone, then diluting with an aqueous solution of 0.05% (*w/v*) Triton X-100 up to 100 mL. The stock solution was serially diluted with 5% (*v/v*) solution of acetone in distilled water containing 0.05% (*w/v*) Triton X-100 to prepare the test solution at concentrations of 100, 50, and 10 mg/L used for the insecticidal activity evaluation against *Plutella xylostella*. The commercial pesticide methoxyfenozide was used as the positive control. The positive control solution at a concentration of 40 mg/L was also prepared by serial dilutions of its stock solution in the same manner. Its concentration was selected according to the crop protection guidelines of the Korea Crop Protection Association. Conversely, the negative control was a 5% (*v/v*) solution of acetone in distilled water containing 0.05% (*w/v*) Triton X-100 without any verbenone derivatives. Fresh cabbage leaves were cut into discs with a diameter of 6 cm. The discs were then dipped in the prepared compound solutions for 30 s and then dried to evaporate water and acetone at room temperature for 3 h. The dried leaf discs were placed into Petri dishes 9 cm in diameter. After that, ten individuals of 3rd instar larvae of *P. xylostella* were transferred to each Petri dish. The Petri dishes were stored in an incubator at 25 ± 1 °C, 65 ± 15% RH (relative humidity), with a 16:8 h light/dark photoperiod. Surviving larvae were counted at 24, 48, 72, and 96 h after transfer. The larvae that did not move when touched with a pen were considered dead. The mortality was calculated by the following equation.

Mortality (%) = (C − T)/C × 100

where C represents the survival rate (%) in the negative control group, and T represents the survival rate (%) in the group of tested compounds. All experiments were carried out in three replicates, and all the mortalities were averaged [21].

### 3.4. QSAR Study

In this study, a 3D QSAR model was constructed using 28 compounds, excluding compounds **7a**–**b** due to their unique scaffold design and limited derivatives for representative classification. First, the compounds were minimized using the ligprep module of Maestro Schrödinger and then aligned using the quick align module. Then, the prepared compounds were randomly divided into two sets, a training set consisting of 70% of the dataset for building the models and a test set consisting of 30% of the dataset for validating the performance of the generated models. The QSAR was calculated using the mortality percent directly under the default Gaussian field of the field-based QSAR module (Maestro Schrödinger version 2021.2). The model generation settings were set to a Maximum PLS factor of 4 and grid spacing of 1.0, thereby balancing accuracy and overfitting prevention. One ligand was left out for cross-validation. The results of the most statistically significant model (model 4) are summarized in Table 5.

## 4. Conclusions

This study aimed to conduct a thorough investigation into the insecticidal activities of a novel series of (1*R*)-(+)-verbenone derivatives against *Plutella xylostella*, commonly known as the diamondback moth. To achieve this, rational design and synthesis approaches were employed to create a diverse range of derivatives with varying chemical structures with the aim of enabling a comprehensive exploration of the structure–activity relationship to better understand the compounds’ insecticidal potential. Among the synthesized compounds, compounds **6a**, **6h**, **6i**, and **6q** exhibited potent insecticidal activity resulting in 100% mortality at 100 mg/L concentration after 4 days. These compounds also exhibited a promising anti-feeding effect on cabbage leaves, which is particularly significant in pest management strategies. To gain a deeper understanding of the structure–activity relationship, a 3D QSAR model was constructed for the synthesized derivatives. This model exhibited a robust correlation (R^2^ = 0.94) with high statistical significance (*p* < 0.001) in predicting the insecticidal activity of the compounds. The 3D QSAR model contours provided insights into the influence of bulky groups, electrostatic contributions, and hydrophobic substitutions on optimizing the insecticidal potency of the compounds. These insights serve as valuable guidance for the design and optimization of novel insecticides based on the (1*R*)-(+)-verbenone scaffold, offering promising avenues for future advancements in insecticide development.

## Figures and Tables

**Table 1:** Insecticidal activity against *P. xylostella* of the (1*S*)-(-)- and (1*R*)-(+)-verbenone derivatives **2a**–**c** and **5a–g** containing heterocyclic aromatic ring.

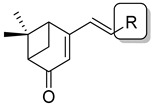				
Entry	Compound	(*R*)/(*S*)	R	Mortality (% ± SD) ^a^
1	**2a**	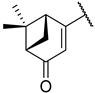	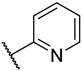	0.0 ± 0.0
2	**2b**	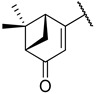	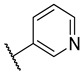	0.0 ± 0.0
3	**2c**	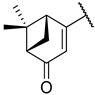	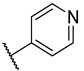	30.0 ± 10.0
4	**5a**	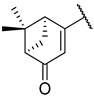	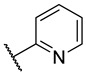	13.3 ± 5.8
5	**5b**	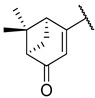	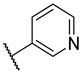	46.7 ± 5.8
6	**5c**	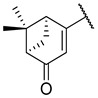	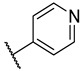	50.0 ± 0.0
7	**5d**	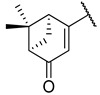	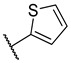	23.3 ± 5.8
8	**5e**	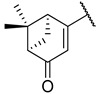	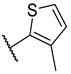	46.7 ± 5.8
9	**5f**	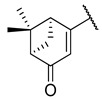	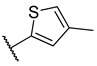	26.7 ± 15.3
10	**5g**	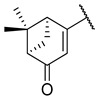	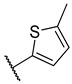	46.7 ± 5.8

^a^ Determined at 100 mg/L concentration after 4 days.

**Table 2:** Insecticidal activity against *P. xylostella* of compounds **6a–q** containing aromatic rings.

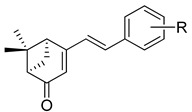			
Entry	Compound	R	Mortality (% ± SD) ^a^
**1**	**6** **a**	**Phenyl**	**100 ± 0.0**
2	**6b**	2-F-Ph	13.3 ± 5.8
3	**6c**	3-F-Ph	16.7 ± 5.8
4	**6d**	4-F-Ph	30.0 ± 17.3
5	**6e**	2,4-F_2_-Ph	0.0 ± 0.0
6	**6f**	3,4-F_2_-Ph	0.0 ± 0.0
7	**6g**	3,5-F_2_-Ph	86.7 ± 5.8
**8**	**6h**	**2,4-Cl_2_-Ph**	**100 ± 0.0**
**9**	**6i**	**3,4-Cl_2_-Ph**	**100 ± 0.0**
10	**6j**	3,5-Cl_2_-Ph	0.0 ± 0.0
**11**	**6k**	**2-OCF_3_-Ph**	**96.7 ± 5.8**
12	**6l**	3-OCF_3_-Ph	30.0 ± 10.0
13	**6m**	4-OCF_3_-Ph	46.7 ± 11.5
14	**6n**	2-Me-Ph	16.7 ± 5.8
15	**6o**	3-Me-Ph	20.0 ± 10.0
16	**6p**	4-Me-Ph	13.3 ± 5.8
**17**	**6q**	**4-Et-Ph**	**100 ± 0.0**

^a^ Determined at 100 mg/L concentration after 4 days.

**Table 3:** Insecticidal activity against *P. xylostella* of compounds **7a–b** possessing saturated linker between verbenone and phenyl ring moieties.

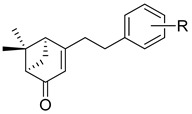			
Entry	Compound	R	Mortality (% ± SD) ^a^
1	**7a**	Phenyl	0.0 ± 0.0
2	**7b**	4-Et-Ph	0.0 ± 0.0

^a^ Determined at 100 mg/L concentration after 4 days.

**Table 4:** Larvicidal activity of compounds **6a**, **6h**, **6i**, and **6q** at different concentrations and times.

Compound	Conc. (mg/L)	Mortality after Incubation (% ± SD)			
		24 h	48 h	72 h	96 h
**6a**	100	0.0 ± 0.0	43.3 ± 5.8	93.3 ± 11.5	100.0 ± 0.0
	50	0.0 ± 0.0	33.3 ± 5.8	53.3 ± 11.5	100.0 ± 0.0
	10	0.0 ± 0.0	23.3 ± 5.8	26.7 ± 5.8	53.3 ± 11.5
**6h**	100	0.0 ± 0.0	40.0 ± 10.0	93.3 ± 5.8	100.0 ± 0.0
	50	0.0 ± 0.0	23.3 ± 5.8	23.3 ± 5.8	50.0 ± 0.0
	10	0.0 ± 0.0	13.3 ± 5.8	16.7 ± 5.8	36.7 ± 5.8
**6i**	100	0.0 ± 0.0	36.7 ± 5.8	90.0 ± 10.0	100.0 ± 0.0
	50	0.0 ± 0.0	20.0 ± 0.0	23.3 ± 5.8	50.0 ± 0.0
	10	0.0 ± 0.0	20.0 ± 10.0	20.0 ± 10.0	33.3 ± 5.8
**6q**	100	0.0 ± 0.0	43.3 ± 5.8	93.3 ± 5.8	100.0 ± 0.0
	50	0.0 ± 0.0	36.7 ± 5.8	36.7 ± 5.8	56.7 ± 5.8
	10	0.0 ± 0.0	23.3 ± 5.8	26.7 ± 5.8	43.3 ± 5.8
methoxyfenozide	40	0.0 ± 0.0	33.3 ± 5.8	73.3 ± 5.8	96.7 ± 5.8
(-) control		0.0 ± 0.0	0.0 ± 0.0	0.0 ± 0.0	0.0 ± 0.0

**Table 5:** Actual vs. predicted mortality for model 4 of 3D QSAR model.

Compound	Experimental Mortality (%)	Predicted Mortality (%)	Prediction Error (%)
**2a**	0.00	−6.4	−6.40
**2b**	0.00	11.37	11.37
**2c**	30.00	16.54	−13.49
**5a**	13.00	22.17	9.17
**5c**	50.00	47.03	−2.97
**5b**	46.70	30.41	−16.29
**5d**	23.30	30.52	7.22
**5e**	46.70	39.14	−7.56
**5f**	26.70	33.08	6.38
**5g**	46.70	19.61	−27.09
**6a**	100.00	24.05	−75.95
**6b**	13.30	14.03	0.73
**6c**	16.70	2.93	−13.77
**6d**	30.00	25.17	−4.83
**6e**	0.00	13.51	13.51
**6f**	0.00	4.25	4.25
**6g**	86.70	80.46	−6.24
**6h**	100.00	98.89	−1.11
**6i**	100.00	99.02	−0.98
**6j**	0.00	−3.86	−3.86
**6k**	96.70	99.83	3.14
**6l**	30.00	30.81	0.81
**6m**	46.70	40.31	−6.39
**6n**	16.70	17.72	1.025
**6o**	20.00	40.33	20.33
**6p**	13.30	19.99	6.69
**6q**	100.00	54.11	−45.89

## Data Availability

The data presented in this study are available on request from the corresponding author.

## Supplementary Materials

The following supporting information can be downloaded at: https://www.mdpi.com/article/10.3390/biom13081272/s1, Additional File S1 of Cantharidin-based verbenone derivatives as a novel insecticide against *Plutella xylostella*: Design, synthesis, insecticidal activity evaluation, and 3D QSAR study. Additional File S1: Images of ^1^H NMR and ^13^C NMR of the synthesized compounds **2a–c**, **5a–g**, **6a–q**, and **7a–b** are available in the Supplementary Information.

## Abbreviations

*DBM*	Diamondback moth
*DDT*	Dichlorodiphenyltrichloroethane
*PP1*	Protein phosphatase 1
*PP2A*	Protein phosphatase 2A
*rPxPP5*	Recombinant *Plutella xylostella* protein serine/threonine phosphatase gene 5
*MeOH*	Methanol
*NaOMe*	Sodium methoxide
*TFA*	Trifluoroacetic acid
*Pd/C*	Palladium on carbon
*^1^H NMR*	Proton nuclear magnetic resonance
*^13^* *C NMR*	Carbon-13 nuclear magnetic resonance
*HRMS*	High-resolution mass spectrometry
*HPLC*	High-performance liquid chromatography

## References

[1] Sarfraz M., Keddie A.B., Dosdall L.M. (2005). Biological control of the diamondback moth, *Plutella xylostella*: A review. Biocontrol Sci. Tech..

[2] Attique M.N., Khaliq A., Sayyed A.H. (2006). Could resistance to insecticides in *Plutella xylostella* (Lep., Plutellidae) be overcome by insecticide mixtures?. J. Appl. Entomol..

[3] Sidhu A. (2017). Understanding the Diamondback Moth (*Plutella xylostella*) Performance in Plant Alternative Cropping Systems, Brock University. http://hdl.handle.net/10464/12955.

[4] Kumar J., Ramlal A., Mallick D., Mishra V. (2021). An overview of some biopesticides and their importance in plant protection for commercial acceptance. Plants.

[5] Liu J., Zhang Y. (2014). Cantharidin impedes the activity of protein serine/threonine phosphatase in *Plutella xylostella*. Mol. Biosyst..

[6] Ye L., Jia Y., Ji K.E., Sanders A.J., Xue K., Ji J., Mason M.D., Jiang W.G. (2015). Traditional Chinese medicine in the prevention and treatment of cancer and cancer metastasis. Oncol. Lett..

[7] Khan R.A., Rashid M., Naveed M. (2022). Cantharidin: A chemical precursor for the development of novel bioinsecticides. Bulg. Chem. Commun..

[8] Wang G., Dong J., Deng L. (2018). Overview of cantharidin and its analogues. Curr. Med. Chem..

[9] Tresch S., Schmotz J., Grossmann K. (2011). Probing mode of action in plant cell cycle by the herbicide endothall, a protein phosphatase inhibitor. Pestic. Biochem. Physiol..

[10] Mumby M.C., Walter G. (1993). Protein serine/threonine phosphatases: Structure, regulation, and functions in cell growth. Physiol. Rev..

[11] Khan R.A., Liu J., Zhang Y. (2014). Catalytic inactivation of alkaline phosphatase by cantharidin, an inhibitor of protein phosphatase. RSC Adv..

[12] Yadav D.K., Saloni S.P., Misra S., Singh H., Mancera R.L., Kumar S. (2018). Studies of the benzopyran class of selective COX-2 inhibitors using 3D-QSAR and molecular docking. Arch. Pharm. Res..

[13] José L.V., Juliana A.M., Alexander F., Julio C. (2019). Structural requirements of n-alpha-mercaptoacetyl dipeptide (namdp) inhibitors of pseudomonas Aeruginosa Virulence factor LasB: 3D-QSAR, molecular docking, and interaction fingerprint studies. Int. J. Mol. Sci..

[14] Varpe B.D., Jadhav S.B., Chatale B.C., Mali A.S., Jadhav S.Y., Kulkarni A.A. (2020). 3D-QSAR and Pharmacophore modeling of 3, 5-disubstituted indole derivatives as Pim kinase inhibitors. Struct. Chem..

[15] Balasubramanian P.K., Balupuri A., Kang H.Y., Cho S.J. (2017). Receptor-guided 3D-QSAR studies, molecular dynamics simulation and free energy calculations of Btk kinase inhibitors. BMC Syst. Biol..

[16] Fan N., Zhang S., Sheng T., Zhao L., Liu Z., Liu J., Wang X. (2018). Docking field-based QSAR and pharmacophore studies on the substituted pyrimidine derivatives targeting HIV-1 reverse transcriptase. Chem. Biol. Drug Des..

[17] Kumari A., Kaur M., Bahia M.S., Silakari O. (2013). 3D-QSAR analysis of anilinoquinoline inhibitors of colony stimulating factor-1 kinase (cFMS): Implementation of field-based molecular alignment. Med. Chem. Res..

[18] Kamaria P., Kawathekar N. (2014). Ligand-based 3D-QSAR analysis and virtual screening in exploration of new scaffolds as Plasmodium falciparum glutathione reductase inhibitors. Med. Chem. Res..

[19] Osman E.A., Abdalla M.A., Abdelraheem M.O., Ali M.F., Osman S.A., Tanir Y.M., Alzain A.A. (2021). Design of novel coumarins as potent Mcl-1 inhibitors for cancer treatment guided by 3D-QSAR, molecular docking and molecular dynamics. Inform. Med. Unlocked.

[20] Islam S., Paul S., Roy A.S., Banerjee S., Ghosh K., Dey R.C., Santra S.C. (2013). Catalytic activity of an iron (III) Schiff base complex bound in a polymer resin. Transit. Met. Chem..

[21] Zhang C., Tian Q., Li Y. (2022). Design, synthesis, and insecticidal activity evaluation of piperine derivatives. Front. Chem..

[22] Zhao Z., Xu Q., Chen W., Wang S., Yang Q., Dong Y., Zhang J. (2022). Rational Design, Synthesis, and Biological Investigations of *N*-Methylcarbamoylguanidinyl Azamacrolides as a Novel Chitinase Inhibitor. J. Agric. Food. Chem..

