# Supplementary material for: Cantharidin-Based Verbenone Derivatives as a Novel Insecticide against Plutella xylostella: Design, Synthesis, Insecticidal Activity Evaluation, and 3D QSAR Study
Source: Biomolecules. 2023 Aug 21;13(8):1272. doi: 10.3390/biom13081272 (PMC10452746; doi:10.3390/biom13081272)
Supplement: Supplementary file 1 [file biomolecules-13-01272-s001.zip › biomolecules-2504965-supplementary.pdf]

SUPPLEMENTARY INFORMATION

Cantharidin-based verbenone derivatives as a novel insecticide against *Plutella xylostella*: Design, synthesis, insecticidal activity evaluation, and 3D QSAR study

(1*S*,5*R*)-6,6-dimethyl-4-((*E*)-2-(pyridin-2-yl)vinyl)bicyclo[3.1.1]hept-3-en-2-one (2a):

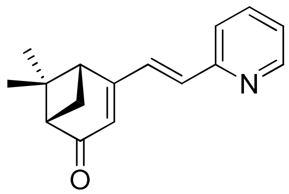

To a stirred solution of (1*S*)-(-)-verbenone (**1**) (200.0 mg, 1.33 mmol) and picolinaldehyde (171.6 mg, 1.60 mmol) in MeOH (7 mL) was added $NaOCH_3$ (108.4 mg, 2.00 mmol). The reaction mixture was stirred at 60 °C for 6 h, and then cooled to room temperature. A few drops of H_2O were added, and it was allowed to stand at room temperature for 24 h. After removal of MeOH by evaporation in vacuo, the reaction mixture was diluted with H_2O, and extracted with ethyl acetate. The combined organic layers were washed with H_2O and brine. It was dried over $MgSO_4$, and evaporated under vacuum (55 °C) to give a crude product. Purification by flash column chromatography (silica gel, 5% ethyl acetate in hexane) afforded **2a** as a brown liquid in 77.4% yield (246.3 mg). ^{1}H NMR ($CDCl_3$, 500 MHz): δ 8.60 (d, *J* = 4.7 Hz, 1H), 7.67 (td, *J* = 7.7, 1.7 Hz, 1H), 7.45 (d, *J* = 15.9 Hz, 1H), 7.38 (d, *J* = 7.9 Hz, 1H), 7.18 (ddd, *J* = 7.5, 4.7, 0.9 Hz, 1H), 6.96 (d, *J* = 15.9 Hz, 1H), 6.02 (s, 1H), 3.11 (t, *J* = 5.6 Hz, 1H), 2.91 (dt, *J* = 9.6, 5.6 Hz, 1H), 2.73 (td, *J* = 5.7, 1.5 Hz, 1H), 2.11 (d, *J* = 9.5 Hz, 1H), 1.56 (s, 3H), 0.99 (s, 3H); ^{13}C NMR ($CDCl_3$, 126 MHz): δ 204.07, 163.65, 154.47, 150.10, 136.80, 134.04, 131.29, 124.43, 123.28, 58.42, 53.05, 44.00, 40.11, 26.86, 22.26; HRMS (FAB) $[M + H]^+$ Calcd for $C_{16}H_{18}NO$: 240.1383, Found: 240.1396; HPLC purity: 98.4%, t_R = 4.9 min.

(1*S*,5*R*)-6,6-dimethyl-4-((*E*)-2-(pyridin-3-yl)vinyl)bicyclo[3.1.1]hept-3-en-2-one (2b):

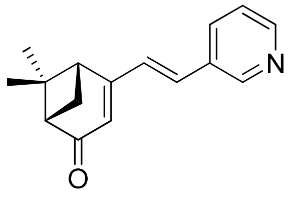

The procedure was similar to that of **2a** and afforded **2b** as a yellow solid in 73.6% yield. mp: 102-104 °C; ^{1}H NMR ($CDCl_3$, 500 MHz): δ 8.68 (d, *J* = 1.9 Hz, 1H), 8.51 (dd, *J* = 4.7, 1.3 Hz, 1H), 7.82

(dt, J = 8.0, 1.9 Hz, 1H), 7.28 (dd, J = 8.0, 4.8 Hz, 1H), 6.99 (d, J = 16.2 Hz, 1H), 6.88 (d, J = 16.2 Hz, 1H), 5.95 (s, 1H), 3.10 (td, J = 5.8, 1.2 Hz, 1H), 2.92 (dt, J = 9.4, 5.6 Hz, 1H), 2.73 (td, J = 5.7, 1.7 Hz, 1H), 2.10 (d, J = 9.5 Hz, 1H), 1.57 (s, 3H), 1.00 (s, 3H); ^{13}C NMR ($CDCl_3$, 126 MHz): δ 203.85, 163.41, 149.94, 149.47, 133.25, 131.85, 131.10, 129.47, 123.74, 58.35, 52.97, 43.77, 40.08, 26.85, 22.26; HRMS (FAB) $[M + H]^+$ Calcd for $C_{16}H_{18}NO$: 240.1383, Found: 240.1393; HPLC purity: 98.5%, t_R = 3.9 min.

(1*S*,5*R*)-6,6-dimethyl-4-((*E*)-2-(pyridin-4-yil)vinyl)bicyclo[3.1.1]hept-3-en-2-one (2c):

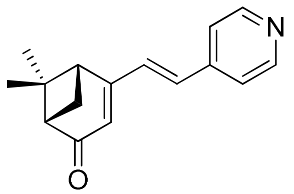

The procedure was similar to that of **2a** and afforded **2c** as a brown liquid in 76.8% yield. ^{1}H NMR ($CDCl_3$, 500 MHz): δ 8.59 (d, J = 5.9 Hz, 2H), 7.32 (d, J = 6.0 Hz, 2H), 7.10 (d, J = 16.2 Hz, 1H), 6.81 (d, J = 16.2 Hz, 1H), 5.99 (s, 1H), 3.07 (t, J = 5.8 Hz, 1H), 2.92 (dt, J = 9.5, 5.6 Hz, 1H), 2.74 (td, J = 5.7, 1.6 Hz, 1H), 2.10 (d, J = 9.5 Hz, 1H), 1.58 (s, 3H), 0.99 (s, 3H); ^{13}C NMR (126 MHz, $cdcl_3$) δ 203.70, 162.91, 150.55, 143.25, 132.02, 131.70, 124.75, 121.37, 58.37, 53.03, 43.82, 40.08, 26.84, 22.26; HRMS (FAB) $[M + H]^+$ Calcd for $C_{16}H_{18}NO$: 240.1383, Found: 240.1390; HPLC purity: 98.1%, t_R = 4.3 min.

(1*R*,5*R*)-4,6,6-trimethylbicyclo[3.1.1]hept-3-en-2-one (4) [1]:

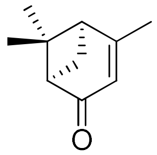

To a two-neck round-bottom flask with acetone (45 mL) and water (5 mL), (*R*)-(+)-α-pinene (**3**) (1.3 g, 0.01 mol) and *N*-hydroxyphthalimide (1.8 g, 0.01 mol) were added once. The mixture was stirred vigorously at room temperature, and then chrominum trioxide (1.0 g, 0.01 mol) was added to the

mixture. After 3 hours of stirring, chrominum trioxide (1.0 g, 0.01 mol) was added once again to the mixture. The reaction mixture was stirred continuously for 20 hours, and then worked up by evaporating the acetone in vacuo. The residue was diluted with dichloromethane and filtered. It was dried over $MgSO_4$, and evaporated under vacuum (55 °C) to give a crude product. Purification by flash column chromatography (silica gel, 5% ethyl acetate in hexane) afforded **4** as a yellow liquid in 50.6% yield. (0.76 g); ^{1}H NMR ($CDCl_3$, 500 MHz): δ 5.71 (dd, *J* = 3.1, 1.5 Hz, 1H), 2.79 (dt, *J* = 9.2, 5.5 Hz, 1H), 2.63 (td, *J* = 6.0, 1.7 Hz, 1H), 2.44 – 2.37 (m, 1H), 2.07 (d, *J* = 9.2 Hz, 1H), 2.00 (d, *J* = 1.5 Hz, 3H), 1.48 (s, 3H), 1.00 (s, 3H); ^{13}C NMR ($CDCl_3$, 126 MHz): δ 203.7, 169.9, 121.1, 57.6, 53.9, 49.7, 40.7, 26.5, 23.4, 22.0; HRMS (FAB) $[M + H]^+$ Calcd for $C_{10}H_{15}O$: 151.1118, Found: 151.1100.

(1*R*,5*S*)-6,6-dimethyl-4-((*E*)-2-(pyridin-2-yl)vinyl)bicyclo[3.1.1]hept-3-en-2-one (5a):

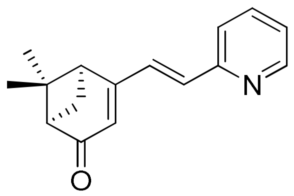

To a stirred solution of (1*R*)-(+)-verbenone (**4**) (200.2 mg, 1.33 mmol) and picolinaldehyde (214.1 mg, 2.00 mmol) in MeOH (7 mL) was added KOH (149.6 mg, 2.66 mmol). The reaction mixture was stirred at 60 °C for 6 h, and then cooled to room temperature. A few drops of H_2O were added, and it was allowed to stand at room temperature for 24 h. After removal of MeOH by evaporation in vacuo, the reaction mixture was diluted with H_2O, and extracted with ethyl acetate. The combined organic layers were washed with H_2O and brine. It was dried over $MgSO_4$, and evaporated under vacuum (55 °C) to give a crude product. Purification by flash column chromatography (silica gel, 5% ethyl acetate in hexane) afforded **5a** as yellow liquid in 47.1% yield (150.3 mg). ^{1}H NMR ($CDCl_3$, 500 MHz): δ 8.59 (ddd, *J* = 4.8, 1.8, 0.8 Hz, 1H), 7.66 (td, *J* = 7.7, 1.8 Hz, 1H), 7.44 (d, *J* = 15.8 Hz, 1H), 7.37 (dt, *J* = 7.9, 0.9 Hz, 1H), 7.17 (ddd, *J* = 7.6, 4.8, 1.1 Hz, 1H), 6.95 (d, *J* = 15.9 Hz, 1H), 6.00 (s, 1H), 3.10 (td, *J* = 5.9, 1.4 Hz, 1H), 2.90 (dt, *J* = 9.5, 5.6 Hz, 1H), 2.72 (td, *J* = 5.7, 1.8 Hz, 1H), 2.10

(d, J = 9.5 Hz, 1H), 1.55 (s, 3H), 0.98 (s, 3H); ^{13}C NMR ($CDCl_3$, 126 MHz): δ 204.00, 163.62, 154.41, 150.06, 136.76, 134.01, 131.22, 124.37, 123.24, 58.37, 52.99, 43.95, 40.06, 26.81, 22.22; HRMS (FAB) $[M + H]^+$ Calcd for $C_{16}H_{18}NO$: 240.1383, Found: 240.1392; HPLC purity: 99.1%, t_R = 3.9 min.

(1*R*,5*S*)-6,6-dimethyl-4-((*E*)-2-(pyridin-3-yl)vinyl)bicyclo[3.1.1]hept-3-en-2-one (5b):

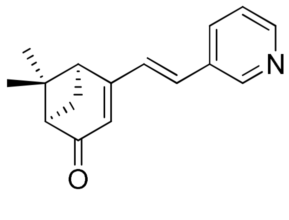

The procedure was similar to that of **5a** and afforded **5b** as a yellow solid in 86.9% yield. mp: 86-89 °C; ^{1}H NMR ($CDCl_3$, 500 MHz): δ 8.69 (d, J = 1.9 Hz, 1H), 8.52 (dd, J = 4.7, 1.4 Hz, 1H), 7.84 (dt, J = 8.0, 1.9 Hz, 1H), 7.30 (dd, J = 8.0, 4.8 Hz, 1H), 6.95 (dd, J = 55.9, 16.2 Hz, 2H), 5.95 (s, 1H), 3.11 (td, J = 5.8, 1.3 Hz, 1H), 2.93 (dt, J = 9.5, 5.6 Hz, 1H), 2.73 (td, J = 5.7, 1.7 Hz, 1H), 2.11 (d, J = 9.5 Hz, 1H), 1.58 (s, 3H), 1.01 (s, 3H); ^{13}C NMR ($CDCl_3$, 126 MHz): δ 203.74, 163.36, 149.83, 149.36, 133.21, 131.78, 131.05, 129.37, 123.70, 123.60, 58.26, 52.87, 43.68, 40.00, 26.76, 22.17; HRMS (FAB) $[M + H]^+$ Calcd for $C_{16}H_{18}NO$: 240.1383, Found: 240.1393; HPLC purity: 98.2%, t_R = 3.4 min.

(1*R*,5*S*)-6,6-dimethyl-4-((*E*)-2-(pyridin-4-yl)vinyl)bicyclo[3.1.1]hept-3-en-2-one (5c):

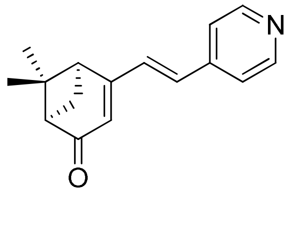

The procedure was similar to that of **5a** and afforded **5c** as a brown liquid in 23.6% yield. ^{1}H NMR ($CDCl_3$, 500 MHz): δ 8.60 (d, J = 5.8 Hz, 2H), 7.33 (dd, J = 4.6, 1.6 Hz, 2H), 7.11 (d, J = 16.1 Hz, 1H), 6.82 (d, J = 16.2 Hz, 1H), 6.00 (s, 1H), 3.08 (td, J = 5.8, 1.4 Hz, 1H), 2.93 (dt, J = 9.5, 5.6 Hz, 1H), 2.75 (td, J = 5.7, 1.8 Hz, 1H), 2.11 (d, J = 9.5 Hz, 1H), 1.58 (s, 3H), 1.00 (s, 3H); ^{13}C NMR ($CDCl_3$, 126 MHz): δ 203.73, 162.93, 150.54, 143.31, 132.03, 131.74, 124.80, 121.38, 58.39, 53.06,

43.85, 40.11, 26.85, 22.27; HRMS (FAB) $[M + H]^+$ Calcd for $C_{16}H_{18}NO$: 240.1383, Found: 240.1392; HPLC purity: 98.6%, $t_R = 3.5$ min.

(1*R*,5*S*)-6,6-dimethyl-4-((E)-2-(thiophen-2-yl)vinyl)bicyclo[3.1.1]hept-3-en-2-one (5d):

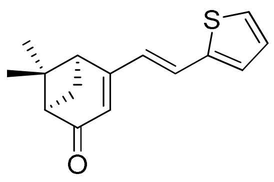

The procedure was similar to that of **5a** and afforded **5d** as a brown liquid in 66.5% yield. ^{1}H NMR ($CDCl_3$, 500 MHz): δ 7.28 (d, *J* = 5.0 Hz, 1H), 7.13 (d, *J* = 3.6 Hz, 1H), 7.04 (d, *J* = 15.9 Hz, 1H), 7.00 (dd, *J* = 5.1, 3.6 Hz, 1H), 6.72 (d, *J* = 15.9 Hz, 1H), 5.86 (s, 1H), 3.02 (td, *J* = 5.9, 1.2 Hz, 1H), 2.87 (dt, *J* = 9.4, 5.6 Hz, 1H), 2.69 (td, *J* = 5.7, 1.7 Hz, 1H), 2.07 (d, *J* = 9.4 Hz, 1H), 1.54 (s, 3H), 0.98 (s, 3H); ^{13}C NMR ($CDCl_3$, 126 MHz): δ 203.86, 163.85, 141.76, 128.72, 128.06, 127.85, 127.04, 126.80, 122.18, 58.22, 52.74, 43.80, 39.95, 26.80, 22.20; HRMS (FAB) $[M + H]^+$ Calcd for $C_{15}H_{17}OS$: 245.0995, Found: 245.1004; HPLC purity: 97.7%, $t_R = 6.2$ min.

(1*R*,5*S*)-6,6-dimethyl-4-((*E*)-2-(3-methylthiophen-2-yl)vinyl)bicyclo[3.1.1]hept-3-en-2-one (5e):

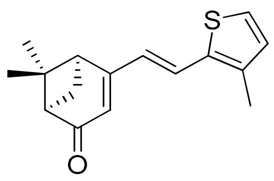

The procedure was similar to that of **5a** and afforded **5e** as a brown liquid in 64.0% yield. ^{1}H NMR ($CDCl_3$, 500 MHz): δ 7.17 (d, *J* = 5.1 Hz, 1H), 7.05 (d, *J* = 15.8 Hz, 1H), 6.81 (dd, *J* = 5.1, 1.6 Hz, 1H), 6.65 (d, *J* = 15.8 Hz, 1H), 5.84 (s, 1H), 3.05 (t, *J* = 5.8 Hz, 1H), 2.91 – 2.85 (m, 1H), 2.68 (td, *J* = 5.7, 1.7 Hz, 1H), 2.29 (s, 3H), 2.08 (dd, *J* = 8.9, 2.1 Hz, 1H), 1.55 (s, 3H), 0.99 (s, 3H); ^{13}C NMR ($CDCl_3$, 126 MHz): δ 203.74, 164.08, 138.70, 135.63, 131.09, 126.15, 125.96, 125.61, 121.58, 58.11, 52.61, 43.81, 39.88, 26.75, 22.11, 14.11; HRMS (FAB) $[M + H]^+$ Calcd for $C_{16}H_{19}OS$: 259.1151, Found: 259.1159; HPLC purity: 96.2%, $t_R = 7.4$ min.

(1*R*,5*S*)-6,6-dimethyl-4-((*E*)-2-(4-methylthiophen-2-yl)vinyl)bicyclo[3.1.1]hept-3-en-2-one (5f):

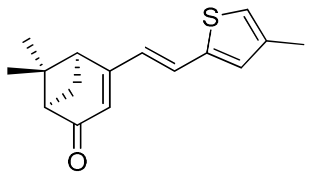

The procedure was similar to that of **5a** and afforded **5f** as a brown solid in 70.6% yield. mp: 69-71 °C; ^{1}H NMR ($CDCl_3$, 500 MHz): δ 6.98 (d, *J* = 15.9 Hz, 1H), 6.96 (d, *J* = 0.7 Hz, 1H), 6.89 (s, 1H), 6.71 (d, *J* = 15.8 Hz, 1H), 5.87 (s, 1H), 3.02 (td, *J* = 5.9, 1.4 Hz, 1H), 2.89 (dt, *J* = 9.4, 5.6 Hz, 1H), 2.71 (td, *J* = 5.7, 1.8 Hz, 1H), 2.23 (s, 3H), 2.09 (d, *J* = 9.4 Hz, 1H), 1.56 (s, 3H), 1.00 (s, 3H); ^{13}C NMR ($CDCl_3$, 126 MHz): δ 204.00, 164.00, 141.54, 138.79, 130.92, 128.03, 126.53, 122.72, 122.12, 58.31, 52.83, 43.92, 40.03, 26.89, 22.28, 15.70; HRMS (FAB) $[M + H]^+$ Calcd for $C_{16}H_{19}OS$: 259.1151, Found: 259.1160; HPLC purity: 97.8%, t_R = 8.1 min.

(1*R*,5*S*)-6,6-dimethyl-4-((*E*)-2-(5-methylthiophen-2-yl)vinyl)bicyclo[3.1.1]hept-3-en-2-one (5g):

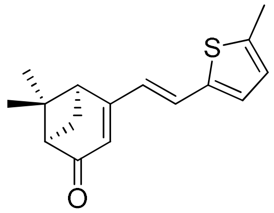

The procedure was similar to that of **5a** and afforded **5g** as a brown liquid in 53.0% yield. ^{1}H NMR ($CDCl_3$, 500 MHz): δ 7.16 (d, *J* = 5.1 Hz, 1H), 7.06 (d, *J* = 15.7 Hz, 1H), 6.81 (d, *J* = 5.1 Hz, 1H), 6.65 (d, *J* = 15.7 Hz, 1H), 5.83 (s, 1H), 3.05 (td, *J* = 5.9, 1.3 Hz, 1H), 2.88 (dt, *J* = 9.3, 5.6 Hz, 1H), 2.68 (td, *J* = 5.7, 1.8 Hz, 1H), 2.28 (s, 3H), 2.06 (d, *J* = 9.3 Hz, 1H), 1.55 (s, 3H), 0.99 (s, 3H); ^{13}C NMR ($CDCl_3$, 126 MHz): δ 203.47, 163.93, 138.59, 135.51, 130.98, 126.05, 125.81, 125.50, 121.44, 57.98, 52.43, 43.66, 39.74, 26.63, 22.00, 14.00; HRMS (FAB) $[M + H]^+$ Calcd for $C_{16}H_{19}OS$: 259.1151, Found: 259.1162; HPLC purity: 97.1%, t_R = 8.3 min.

(1*R*,5*S*)-6,6-dimethyl-4-((*E*)-styryl)bicyclo[3.1.1]hept-3-en-2-one (6a):

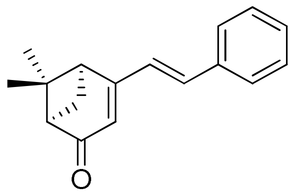

The procedure was similar to that of **5a** and afforded **6a** as a brown liquid in 87.0% yield. ^{1}H NMR ($CDCl_3$, 500 MHz): δ 7.52 – 7.48 (m, 2H), 7.39 – 7.35 (m, 2H), 7.34 – 7.30 (m, 1H), 6.94 (q, *J* = 16.2 Hz, 2H), 5.93 (s, 1H), 3.12 (t, *J* = 5.8 Hz, 1H), 2.92 (dt, *J* = 9.5, 5.6 Hz, 1H), 2.73 (td, *J* = 5.7, 1.5 Hz, 1H), 2.12 (d, *J* = 9.4 Hz, 1H), 1.58 (s, 3H), 1.01 (s, 3H); ^{13}C NMR ($CDCl_3$, 126 MHz): δ 204.36, 164.41, 136.04, 135.12, 129.28, 128.99, 127.47, 122.76, 58.29, 53.03, 43.76, 40.16, 26.88, 22.27; HRMS (FAB) $[M + H]^+$ Calcd for $C_{17}H_{19}O$: 239.1431, Found: 239.1440; HPLC purity: 99.8%, t_R = 6.8 min.

(1*R*,5*S*)-4-(2-fluorostyryl)-6,6-dimethylbicyclo[3.1.1]hept-3-en-2-one (6b):

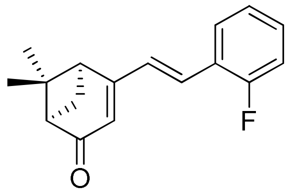

The procedure was similar to that of **5a** and afforded **6b** as a yellow solid in 78.8% yield. mp: 65-67 °C; ^{1}H NMR ($CDCl_3$, 500 MHz): δ 7.58 (t, *J* = 7.2 Hz, 1H), 7.32 – 7.21 (m, 1H), 7.18 – 6.94 (m, 4H), 5.93 (s, 1H), 3.18 – 3.03 (m, 1H), 2.96 – 2.82 (m, 1H), 2.76 – 2.65 (m, 1H), 2.09 (d, *J* = 9.4 Hz, 1H), 1.56 (s, 3H), 1.00 (s, 3H); ^{13}C NMR ($CDCl_3$, 126 MHz): δ 203.92, 164.00, 161.66, 130.37, 129.22, 127.38, 126.79, 124.37, 123.81, 123.19, 116.00, 58.12, 52.78, 43.35, 39.93, 26.65, 22.05; HRMS (FAB) $[M + H]^+$ Calcd for $C_{17}H_{18}FO$: 257.1336, Found: 257.1344; HPLC purity: 99.4%, t_R = 7.3 min.

(1*R*,5*S*)-4-(3-fluorostyryl)-6,6-dimethylbicyclo[3.1.1]hept-3-en-2-one (6c):

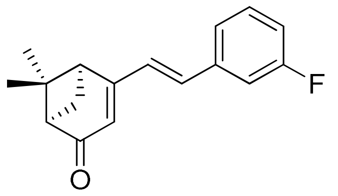

The procedure was similar to that of **5a** and afforded **6c** as a yellow solid in 93.9% yield. mp: 58-60 °C; ^{1}H NMR (CDCl$_3$, 500 MHz): δ 7.47 – 7.12 (m, 3H), 7.07 – 6.79 (m, 3H), 5.93 (s, 1H), 3.09 (s, 1H), 3.00 – 2.84 (m, 1H), 2.72 (s, 1H), 2.10 (d, J = 9.3 Hz, 1H), 1.57 (s, 3H), 1.00 (s, 3H); ^{13}C NMR (CDCl$_3$, 126 MHz): δ 203.94, 163.68, 162.05, 138.30, 133.56, 130.38, 128.60, 123.30, 115.80, 113.64, 58.16, 52.87, 43.58, 39.98, 26.71, 22.12; HRMS (FAB) [M + H]$^+$ Calcd for $C_{17}H_{18}FO$: 257.1336, Found: 257.1348; HPLC purity: >99.9%, t_R = 6.9 min.

(1*R*,5*S*)-4-(4-fluorostyryl)-6,6-dimethylbicyclo[3.1.1]hept-3-en-2-one (6d):

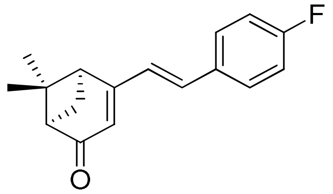

The procedure was similar to that of **5a** and afforded **6d** as a yellow liquid in 65.4% yield. ^{1}H NMR (CDCl$_3$, 500 MHz): δ 7.54 – 7.38 (m, 2H), 7.10 – 6.97 (m, 2H), 6.92 – 6.79 (m, 2H), 5.91 (s, 1H), 3.10 (t, J = 5.6 Hz, 1H), 2.90 (dt, J = 9.4, 5.6 Hz, 1H), 2.75 – 2.65 (m, 1H), 2.09 (d, J = 9.4 Hz, 1H), 1.56 (s, 3H), 1.00 (s, 3H); ^{13}C NMR (CDCl$_3$, 126 MHz): δ 203.88, 163.99, 161.96, 133.57, 132.15, 129.01, 128.95, 127.00, 122.49, 115.90, 115.73, 58.04, 52.71, 43.47, 39.88, 26.61, 22.02; HRMS (FAB) [M + H]$^+$ Calcd for $C_{17}H_{18}FO$: 257.1336, Found: 257.1349; HPLC purity: 99.8%, t_R = 6.7 min.

(1*R*,5*S*)-4-(2,4-difluorostyryl)-6,6-dimethylbicyclo[3.1.1]hept-3-en-2-one (6e):

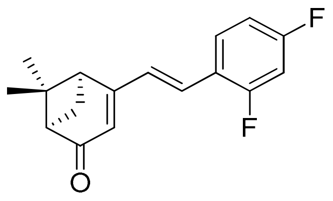

The procedure was similar to that of **5a** and afforded **6e** as a yellow liquid in 32.1% yield. ^{1}H NMR ($CDCl_3$, 500 MHz): δ 7.55 (td, *J* = 8.6, 6.4 Hz, 1H), 6.95 (q, *J* = 16.4 Hz, 2H), 6.90 – 6.84 (m, 1H), 6.81 (ddd, *J* = 11.1, 8.7, 2.5 Hz, 1H), 5.91 (s, 1H), 3.08 (td, *J* = 5.8, 1.4 Hz, 1H), 2.90 (dt, *J* = 9.4, 5.6 Hz, 1H), 2.71 (td, *J* = 5.7, 1.8 Hz, 1H), 2.08 (d, *J* = 9.4 Hz, 1H), 1.56 (s, 3H), 0.99 (s, 3H); ^{13}C NMR ($CDCl_3$, 126 MHz): δ 203.88, 163.88, 162.01, 159.89, 129.15, 128.58, 125.89, 123.37, 120.57, 112.00, 104.44, 58.32, 52.88, 43.67, 40.05, 26.80, 22.19; HRMS (FAB) $[M + H]^+$ Calcd for $C_{17}H_{17}F_2O$: 275.1242, Found: 275.1268; HPLC purity: 97.6%, t_R = 7.9 min.

(1*R*,5*S*)-4-(3,4-difluorostyryl)-6,6-dimethylbicyclo[3.1.1]hept-3-en-2-one (6f):

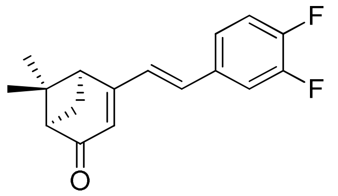

The procedure was similar to that of **5a** and afforded **6f** as a yellow solid in 26.5% yield. mp: 81-83 °C; ^{1}H NMR ($CDCl_3$, 500 MHz): δ 7.23 (dd, *J* = 12.3, 2.1 Hz, 1H), 7.17 (d, *J* = 8.6 Hz, 1H), 6.92 (t, *J* = 8.5 Hz, 1H), 6.86 – 6.69 (m, 2H), 5.87 (s, 1H), 3.06 (t, *J* = 5.4 Hz, 1H), 2.88 (dt, *J* = 9.4, 5.6 Hz, 1H), 2.69 (td, *J* = 5.7, 1.6 Hz, 1H), 2.07 (d, *J* = 9.4 Hz, 1H), 1.55 (s, 3H), 0.98 (s, 3H); ^{13}C NMR ($CDCl_3$, 126 MHz): δ 203.99, 164.14, 151.58, 148.53, 133.59, 129.53, 126.58, 124.26, 122.36, 114.28, 113.44, 58.26, 52.81, 43.86, 40.02, 26.80, 22.20; MS (ESI) $[M + H]^+$ Calcd for $C_{17}H_{17}F_2O$: 275.1242, Found: 275.06; HPLC purity: 97.4%, t_R = 6.2 min.

(1*R*,5*S*)-4-(3,5-difluorostyryl)-6,6-dimethylbicyclo[3.1.1]hept-3-en-2-one (6g):

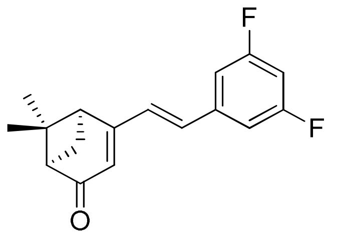

The procedure was similar to that of **5a** and afforded **6g** as a yellow solid in 51.3% yield. mp: 106-

108 °C; ^{1}H NMR ($CDCl_3$, 500 MHz): δ 7.09 – 6.89 (m, 3H), 6.87 – 6.66 (m, 2H), 5.96 (s, 1H), 3.14 – 3.01 (m, 1H), 2.99 – 2.85 (m, 1H), 2.80 – 2.66 (m, 1H), 2.10 (d, *J* = 9.2 Hz, 1H), 1.58 (s, 3H), 1.00 (s, 3H); ^{13}C NMR ($CDCl_3$, 126 MHz): δ 203.80, 164.24, 163.14, 162.27, 139.34, 132.36, 129.85, 124.04, 110.00, 109.79, 104.13, 58.18, 52.93, 43.59, 39.98, 26.69, 22.11; HRMS (FAB) $[M + H]^+$ Calcd for $C_{17}H_{17}F_2O$: 275.1242, Found: 275.1243; HPLC purity: 99.4%, t_R = 7.7 min.

(1*R*,5*S*)-4-(2,4-dichlorostyryl)-6,6-dimethylbicyclo[3.1.1]hept-3-en-2-one (6h):

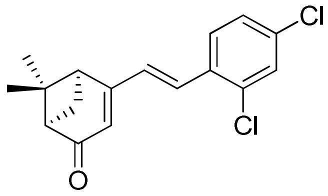

The procedure was similar to that of **5a** and afforded **6h** as a yellow solid in 56.5% yield. mp: 117-119 °C; ^{1}H NMR ($CDCl_3$, 500 MHz): δ 7.59 (d, *J* = 8.2 Hz, 1H), 7.38 (s, 1H), 7.31 – 7.14 (m, 2H), 6.90 (d, *J* = 16.1 Hz, 1H), 5.95 (s, 1H), 3.19 – 3.04 (m, 1H), 3.01 – 2.84 (m, 1H), 2.80 – 2.64 (m, 1H), 2.11 (d, *J* = 9.2 Hz, 1H), 1.58 (s, 3H), 1.01 (s, 3H); ^{13}C NMR ($CDCl_3$, 126 MHz): δ 203.93, 163.61, 134.99, 134.65, 132.63, 130.13, 129.80, 129.28, 127.62, 127.56, 123.92, 58.21, 52.98, 43.67, 40.08, 26.79, 22.17; HRMS (FAB) $[M + H]^+$ Calcd for $C_{17}H_{17}Cl_2O$: 307.0651, Found: 307.0665; HPLC purity: 99.8%, t_R = 16.8 min.

(1*R*,5*S*)-4-(3,4-dichlorostyryl)-6,6-dimethylbicyclo[3.1.1]hept-3-en-2-one (6i):

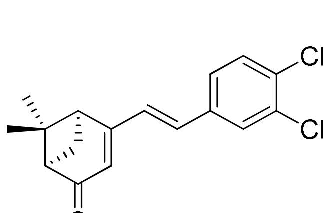

The procedure was similar to that of **5a** and afforded **6i** as a yellow solid in 31.3% yield. mp: 94-96 °C; ^{1}H NMR ($CDCl_3$, 500 MHz): δ 7.56 (s, 1H), 7.41 (d, *J* = 8.2 Hz, 1H), 7.31 (d, *J* = 7.5 Hz, 1H), 6.85 (dd, *J* = 64.7, 16.1 Hz, 2H), 5.94 (s, 1H), 3.12 – 3.01 (m, 1H), 2.97 – 2.84 (m, 1H), 2.78 – 2.66

(m, 1H), 2.10 (d, *J* = 9.4 Hz, 1H), 1.57 (s, 3H), 0.99 (s, 3H); ^{13}C NMR ($CDCl_3$, 126 MHz): δ 203.98, 163.43, 136.15, 133.15, 132.81, 132.23, 130.85, 129.18, 128.97, 126.36, 123.74, 58.25, 53.00, 43.66, 40.08, 26.81, 22.22; HRMS (FAB) $[M + H]^+$ Calcd for $C_{17}H_{17}Cl_2O$: 307.0651, Found: 307.0667; HPLC purity: 99.5%, t_R = 13.1 min.

(1*R*,5*S*)-4-(3,5-dichlorostyryl)-6,6-dimethylbicyclo[3.1.1]hept-3-en-2-one (6j):

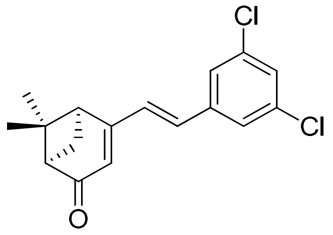

The procedure was similar to that of **5a** and afforded **6j** as a yellow solid in 51.4% yield. mp: 122-124 °C; ^{1}H NMR ($CDCl_3$, 500 MHz): δ 7.33 (d, *J* = 1.8 Hz, 2H), 7.24 (t, *J* = 1.8 Hz, 1H), 6.84 (dd, *J* = 86.8, 16.1 Hz, 2H), 5.96 (s, 1H), 3.05 (td, *J* = 5.8, 1.1 Hz, 1H), 2.92 (dt, *J* = 9.5, 5.6 Hz, 1H), 2.73 (td, *J* = 5.7, 1.7 Hz, 1H), 2.10 (d, *J* = 9.5 Hz, 1H), 1.57 (s, 3H), 0.99 (s, 3H); ^{13}C NMR ($CDCl_3$, 126 MHz): δ 03.72, 163.05, 138.97, 135.38, 131.80, 129.95, 128.52, 125.47, 124.08, 58.16, 52.91, 43.55, 39.96, 26.71, 22.13; HRMS (FAB) $[M + H]^+$ Calcd for $C_{17}H_{17}Cl_2O$: 307.0651, Found: 307.0662; HPLC purity: 99.9%, t_R = 15.5 min.

(1*R*,5*S*)-6,6-dimethyl-4-(2-(trifluoromethoxy)styryl)bicyclo[3.1.1]hept-3-en-2-one (6k):

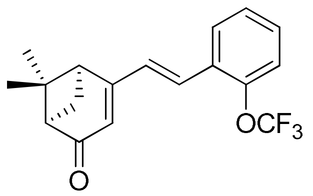

The procedure was similar to that of **5a** and afforded **6k** as a yellow liquid in 56.3% yield. ^{1}H NMR ($CDCl_3$, 500 MHz): δ 7.70 (dd, *J* = 7.4, 2.1 Hz, 1H), 7.30 (pd, *J* = 7.3, 1.7 Hz, 2H), 7.26 – 7.21 (m, 1H), 7.07 (dd, *J* = 85.2, 16.3 Hz, 2H), 5.96 (s, 1H), 3.09 (t, *J* = 5.8 Hz, 1H), 2.90 (dt, *J* = 9.5, 5.6 Hz, 1H), 2.72 (td, *J* = 5.7, 1.5 Hz, 1H), 2.08 (d, *J* = 9.4 Hz, 1H), 1.56 (s, 3H), 0.99 (s, 3H); ^{13}C NMR

(CDCl$_3$, 126 MHz): δ 203.21, 163.42, 146.76, 129.82, 129.77, 129.21, 127.00, 126.88, 126.78, 123.39, 121.14, 121.13, 58.00, 52.54, 43.53, 39.58, 26.38, 21.73; HRMS (FAB) [M + H]$^+$ Calcd for $C_{18}H_{18}F_3O_2$: 323.1254, Found: 323.1276; HPLC purity: 98.6%, t_R = 10.9 min.

(1*R*,5*S*)-6,6-dimethyl-4-(3-(trifluoromethoxy)styryl)bicyclo[3.1.1]hept-3-en-2-one (6l):

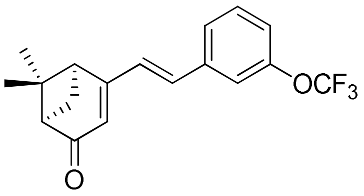

The procedure was similar to that of **5a** and afforded **6l** as a yellow liquid in 59.5% yield. ^{1}H NMR (CDCl$_3$, 500 MHz): δ 7.42 (dt, *J* = 7.7, 1.1 Hz, 1H), 7.37 (t, *J* = 7.9 Hz, 1H), 7.32 (s, 1H), 7.17 – 7.11 (m, 1H), 6.92 (dd, *J* = 37.5, 16.2 Hz, 2H), 5.95 (s, 1H), 3.09 (td, *J* = 5.8, 1.3 Hz, 1H), 2.91 (dt, *J* = 9.4, 5.6 Hz, 1H), 2.73 (td, *J* = 5.7, 1.7 Hz, 1H), 2.10 (d, *J* = 9.4 Hz, 1H), 1.57 (s, 3H), 1.00 (s, 3H); ^{13}C NMR (CDCl$_3$, 126 MHz): δ 203.81, 163.56, 149.75, 138.23, 133.19, 130.23, 129.04, 125.63, 123.53, 121.17, 119.52, 58.27, 52.87, 43.78, 39.98, 26.69, 22.11; MS (ESI) [M + H]$^+$ Calcd for $C_{18}H_{18}F_3O_2$: 323.1254, Found: 323.06; HPLC purity: 99.3%, t_R = 10.8 min.

(1*R*,5*S*)-6,6-dimethyl-4-(4-(trifluoromethoxy)styryl)bicyclo[3.1.1]hept-3-en-2-one (6m):

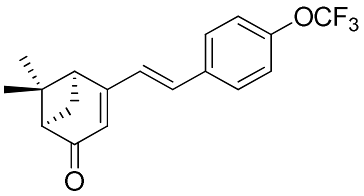

The procedure was similar to that of **5a** and afforded **6m** as a yellow liquid in 43.1% yield. ^{1}H NMR (CDCl$_3$, 500 MHz): δ 7.51 (d, *J* = 8.5 Hz, 2H), 7.19 (d, *J* = 8.4 Hz, 2H), 6.98 – 6.84 (m, 2H), 5.93 (s, 1H), 3.09 (t, *J* = 5.7 Hz, 1H), 2.97 – 2.86 (m, 1H), 2.72 (t, *J* = 5.6 Hz, 1H), 2.10 (d, *J* = 9.4 Hz, 1H), 1.57 (s, 3H), 1.00 (s, 3H); ^{13}C NMR (CDCl$_3$, 126 MHz): δ 203.81, 163.75, 149.52, 134.80, 133.17, 128.68, 128.39, 123.20, 121.21, 58.29, 52.85, 43.81, 40.00, 26.74, 22.15; MS (ESI) [M + H]$^+$ Calcd

for $C_{18}H_{18}F_3O_2$: 323.1254, Found: 323.02; HPLC purity: 99.8%, t_R = 10.8 min.

(1*R*,5*S*)-6,6-dimethyl-4-(2-methylstyryl)bicyclo[3.1.1]hept-3-en-2-one (6n):

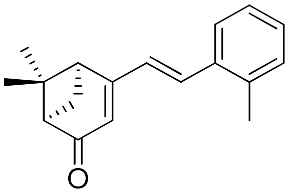

The procedure was similar to that of **5a** and afforded **6n** as a yellow liquid in 55.1% yield. ^{1}H NMR ($CDCl_3$, 500 MHz): δ 7.60 – 7.51 (m, 1H), 7.25 – 7.10 (m, 4H), 6.85 (d, *J* = 16.0 Hz, 1H), 5.91 (s, 1H), 3.10 (t, *J* = 5.7 Hz, 1H), 2.91 (dt, *J* = 9.4, 5.6 Hz, 1H), 2.75 – 2.67 (m, 1H), 2.38 (s, 3H), 2.12 (d, *J* = 9.4 Hz, 1H), 1.57 (s, 3H), 1.01 (s, 3H); ^{13}C NMR ($CDCl_3$, 126 MHz): δ 204.15, 164.53, 136.61, 134.85, 132.42, 130.70, 128.97, 128.50, 126.40, 125.73, 122.55, 58.17, 52.92, 43.82, 40.07, 26.82, 22.15, 19.85; HRMS (FAB) $[M + H]^+$ Calcd for $C_{18}H_{21}O$: 253.1587, Found: 253.1593; HPLC purity: 99.3%, t_R = 8.3 min.

(1*R*,5*S*)-6,6-dimethyl-4-(3-methylstyryl)bicyclo[3.1.1]hept-3-en-2-one (6o):

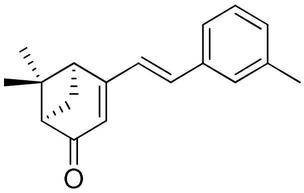

The procedure was similar to that of **5a** and afforded **6o** as a yellow liquid in 61.8% yield. ^{1}H NMR ($CDCl_3$, 500 MHz): δ 7.37 – 7.19 (m, 3H), 7.12 (d, *J* = 7.3 Hz, 1H), 6.91 (q, *J* = 16.2 Hz, 2H), 5.91 (s, 1H), 3.11 (t, *J* = 5.8 Hz, 1H), 2.90 (dt, *J* = 9.4, 5.6 Hz, 1H), 2.72 (td, *J* = 5.8, 1.7 Hz, 1H), 2.35 (s, 3H), 2.10 (d, *J* = 9.4 Hz, 1H), 1.56 (s, 3H), 1.00 (s, 3H); ^{13}C NMR ($CDCl_3$, 126 MHz): δ 204.20, 164.41, 138.46, 135.87, 135.22, 130.04, 128.77, 128.00, 127.14, 124.66, 122.47, 58.18, 52.87, 43.64, 40.02, 26.78, 22.16, 21.40; HRMS (FAB) $[M + H]^+$ Calcd for $C_{18}H_{21}O$: 253.1587, Found: 253.1598; HPLC purity: 98.6%, t_R = 8.9 min.

(1*R*,5*S*)-6,6-dimethyl-4-(4-methylstyryl)bicyclo[3.1.1]hept-3-en-2-one (6p):

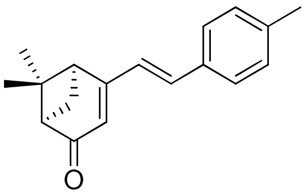

The procedure was similar to that of **5a** and afforded **6p** as a yellow solid in 89.4% yield. mp: 101-103 °C; ^{1}H NMR ($CDCl_3$, 500 MHz): δ 7.38 (d, *J* = 8.1 Hz, 2H), 7.16 (d, *J* = 7.8 Hz, 2H), 6.89 (s, 2H), 5.89 (s, 1H), 3.10 (t, *J* = 5.8 Hz, 1H), 2.89 (dt, *J* = 9.4, 5.6 Hz, 1H), 2.71 (td, *J* = 5.8, 1.7 Hz, 1H), 2.34 (s, 3H), 2.09 (d, *J* = 9.4 Hz, 1H), 1.56 (s, 3H), 1.00 (s, 3H); ^{13}C NMR ($CDCl_3$, 126 MHz): δ 204.19, 164.52, 139.40, 135.07, 133.19, 129.62, 127.34, 126.35, 122.16, 58.16, 52.81, 43.63, 40.02, 26.77, 22.16, 21.43; HRMS (FAB) $[M + H]^+$ Calcd for $C_{18}H_{21}O$: 253.1587, Found: 253.1597; HPLC purity: 99.9%, t_R = 8.8 min.

(1*R*,5*S*)-4-(4-ethylstyryl)-6,6-dimethylbicyclo[3.1.1]hept-3-en-2-one (6q):

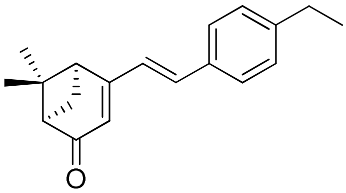

The procedure was similar to that of **5a** and afforded **6q** as a yellow solid in 86.9% yield. mp: 89-91 °C; ^{1}H NMR ($CDCl_3$, 500 MHz): δ 7.39 (d, *J* = 8.1 Hz, 2H), 7.16 (d, *J* = 8.0 Hz, 2H), 6.89 (s, 2H), 5.88 (s, 1H), 3.15 – 3.04 (m, 1H), 2.87 (dt, *J* = 9.4, 5.6 Hz, 1H), 2.74 – 2.66 (m, 1H), 2.64 – 2.54 (m, 2H), 2.07 (d, *J* = 9.3 Hz, 1H), 1.54 (s, 3H), 1.20 (t, *J* = 7.6 Hz, 3H), 0.98 (s, 3H); ^{13}C NMR ($CDCl_3$, 126 MHz): δ 203.84, 164.32, 145.53, 134.93, 133.28, 128.25, 127.29, 126.21, 121.97, 57.98, 52.58, 43.43, 39.80, 28.57, 26.59, 21.98, 15.29; HRMS (FAB) $[M + H]^+$ Calcd for $C_{19}H_{23}O$: 267.1744, Found: 267.1758; HPLC purity: 99.8%, t_R = 11.4 min.

(1*R*,5*S*)-6,6-dimethyl-4-phenethylbicyclo[3.1.1]hept-3-en-2-one (7a):

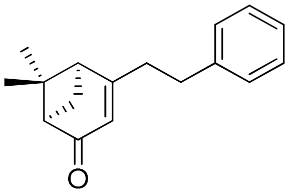

To a stirred solution of **6a** (220.1 mg, 0.92 mmol) in ethyl acetate (5 mL) was added 10% Pd/C (97.2 mg). The reaction mixture was stirred under H_2 (1 atm, balloon) for 6 h. After completion of the reaction, the catalyst was removed by filtration. The filtrate was concentrated in vacuo, and diluted with H_2O, then extracted with ethyl acetate. The combined organic layers were washed with H_2O and brine. It was dried over $MgSO_4$, and evaporated under vacuum (55 °C) to give a crude product. Purification by flash column chromatography (silica gel, 14% ethyl acetate in hexane) afforded **7a** as colorless liquid in 37.1% yield (82.0 mg). ^{1}H NMR ($CDCl_3$, 500 MHz): δ 7.31 – 7.26 (m, 2H), 7.22 – 7.16 (m, 3H), 5.75 (p, *J* = 1.5 Hz, 1H), 2.83 – 2.77 (m, 3H), 2.67 – 2.63 (m, 1H), 2.62 – 2.56 (m, 2H), 2.51 – 2.47 (m, 1H), 2.05 (d, *J* = 9.2 Hz, 1H), 1.50 (s, 3H), 1.00 (s, 3H); ^{13}C NMR ($CDCl_3$, 126 MHz): δ 203.97, 172.41, 140.72, 128.60, 128.29, 126.34, 120.52, 57.97, 54.05, 48.89, 41.08, 38.67, 32.78, 26.69, 22.42; HRMS (FAB) $[M + H]^+$ Calcd for $C_{17}H_{21}O$: 241.1587, Found: 241.1593; HPLC purity: 99.0%, t_R = 7.2 min.

(1*R*,5*S*)-4-(4-ethylphenethyl)-6,6-dimethylbicyclo[3.1.1]hept-3-en-2-one (7b):

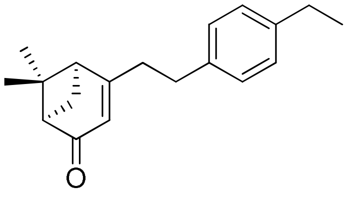

The procedure was similar to that of **7a** and afforded **7b** from **30** as a colorless liquid in 51.9% yield. ^{1}H NMR ($CDCl_3$, 500 MHz): δ 7.11 (q, *J* = 8.1 Hz, 4H), 5.75 (s, 1H), 2.79 (ddd, *J* = 25.7, 13.3, 6.7 Hz, 3H), 2.68 – 2.53 (m, 5H), 2.50 (t, *J* = 5.7 Hz, 1H), 2.05 (d, *J* = 9.1 Hz, 1H), 1.50 (s, 3H), 1.23 (t, *J* = 7.6 Hz, 3H), 1.00 (s, 3H); ^{13}C NMR ($CDCl_3$, 126 MHz): δ 203.99, 172.57, 142.24, 137.87, 128.20, 128.05, 120.45, 57.96, 54.02, 48.88, 41.07, 38.78, 32.35, 28.49, 26.68, 22.41, 15.67; HRMS (FAB) [M

+ H]$^+$ Calcd for $C_{19}H_{25}O$: 269.1900, Found: 269.1916; HPLC purity: 99.4%, t_R = 12.5 min.

2a (LMT-357)

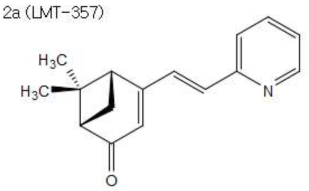

^{1}H NMR (500 MHz, cdcl$_3$) δ 8.60 (d, J = 4.7 Hz, 1H), 7.67 (td, J = 7.7, 1.7 Hz, 1H), 7.45 (d, J = 15.9 Hz, 1H), 7.38 (d, J = 7.9 Hz, 1H), 7.18 (ddd, J = 7.5, 4.7, 0.9 Hz, 1H), 6.96 (d, J = 15.9 Hz, 1H), 6.02 (s, 1H), 3.11 (t, J = 5.6 Hz, 1H), 2.91 (dt, J = 9.6, 5.6 Hz, 1H), 2.73 (td, J = 5.7, 1.5 Hz, 1H), 2.11 (d, J = 9.5 Hz, 1H), 1.56 (s, 3H), 0.99 (s, 3H).

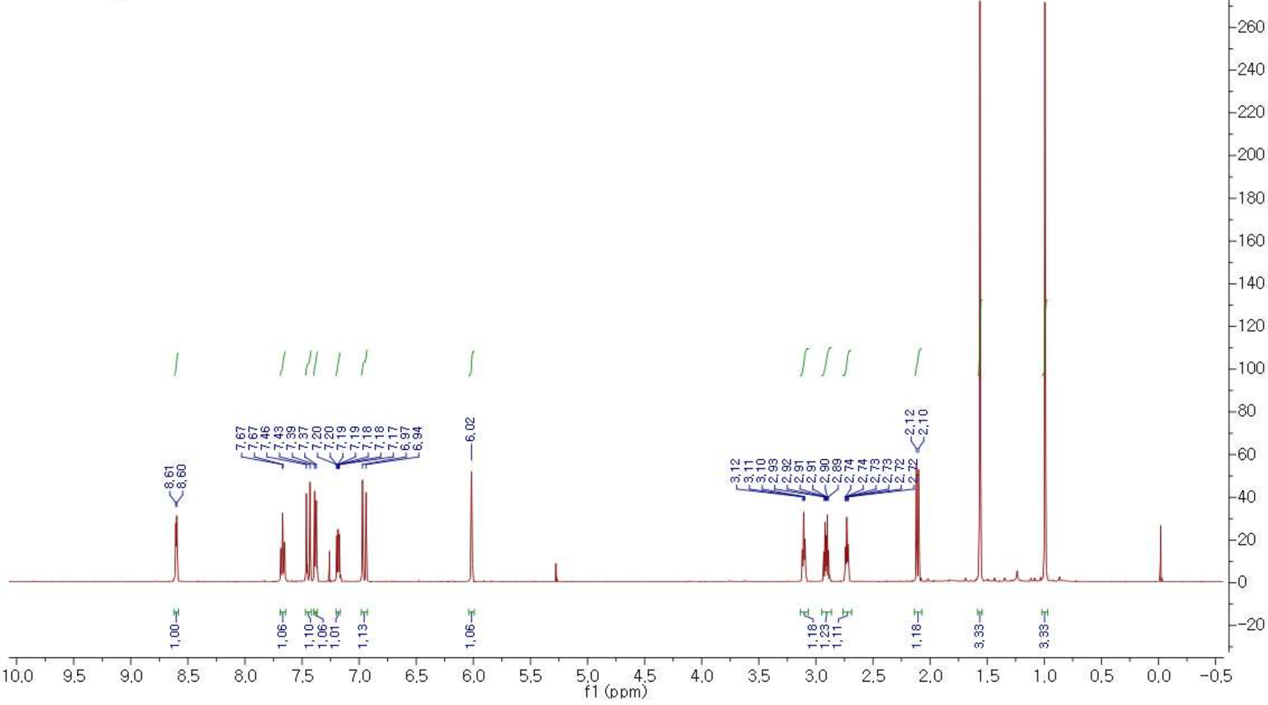

2a (LMT-357)

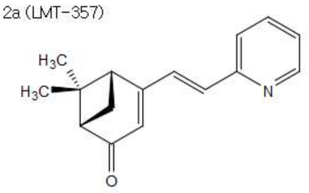

^{13}C NMR (126 MHz, cdcl$_3$) δ 204.07, 163.65, 154.47, 150.10, 136.80, 134.04, 131.29, 124.43, 123.28, 58.42, 53.05, 44.00, 40.11, 26.86, 22.26.

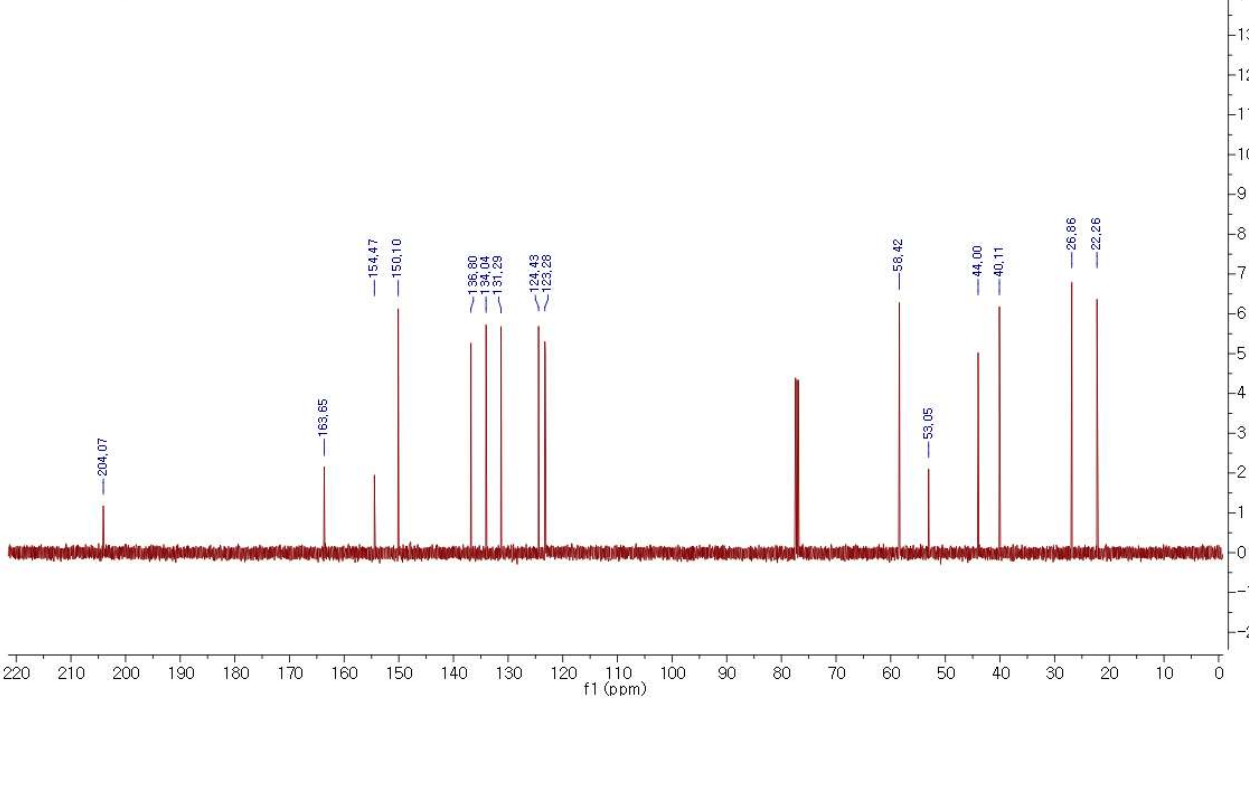

2b (LMT-358)

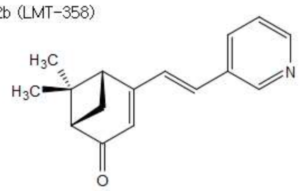

^{1}H NMR (500 MHz, cdcl$_3$) δ 8.68 (d, J = 1.9 Hz, 1H), 8.51 (dd, J = 4.7, 1.3 Hz, 1H), 7.82 (dt, J = 8.0, 1.9 Hz, 1H), 7.28 (dd, J = 8.0, 4.8 Hz, 1H), 6.99 (d, J = 16.2 Hz, 1H), 6.88 (d, J = 16.2 Hz, 1H), 5.95 (s, 1H), 3.10 (td, J = 5.8, 1.2 Hz, 1H), 2.92 (dt, J = 9.4, 5.6 Hz, 1H), 2.73 (td, J = 5.7, 1.7 Hz, 1H), 2.10 (d, J = 9.5 Hz, 1H), 1.57 (s, 3H), 1.00 (s, 3H).

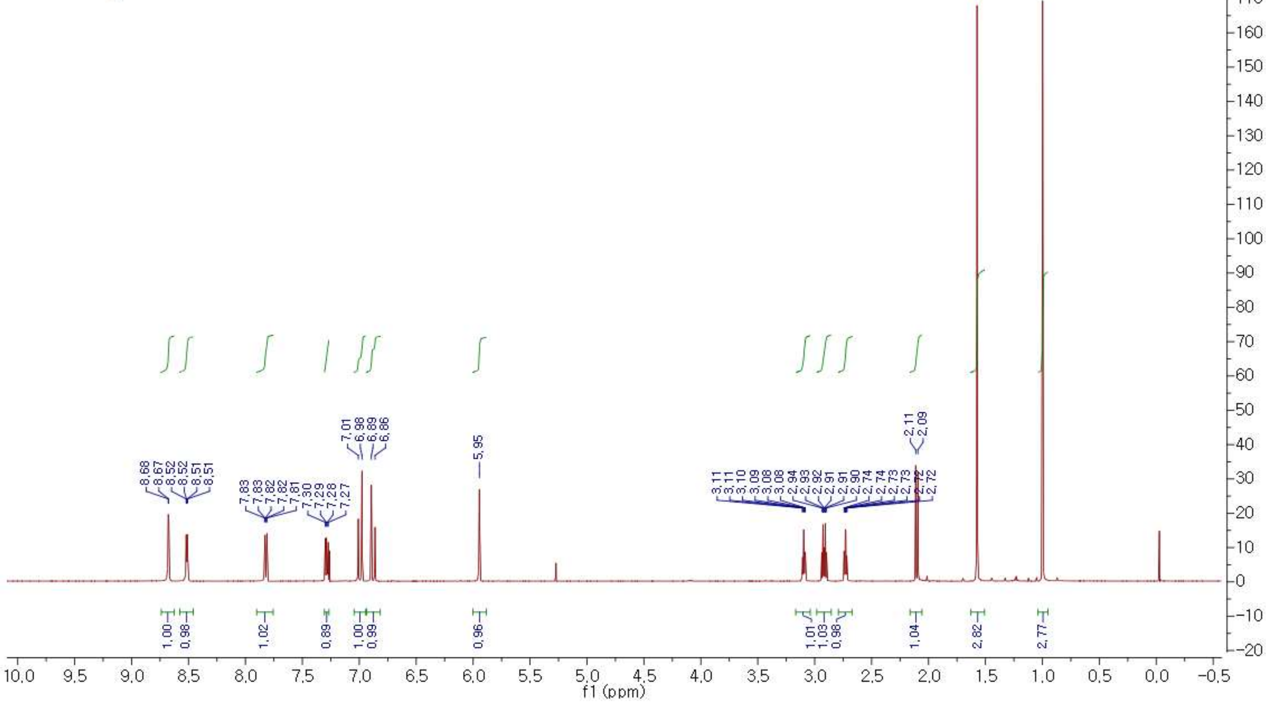

2b (LMT-358)

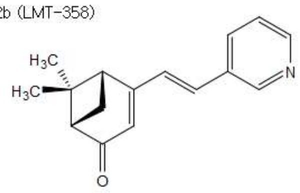

^{13}C NMR (126 MHz, cdcl$_3$) δ 203.85, 163.41, 149.94, 149.47, 133.25, 131.85, 131.10, 129.47, 123.74, 58.35, 52.97, 43.77, 40.08, 26.85, 22.26.

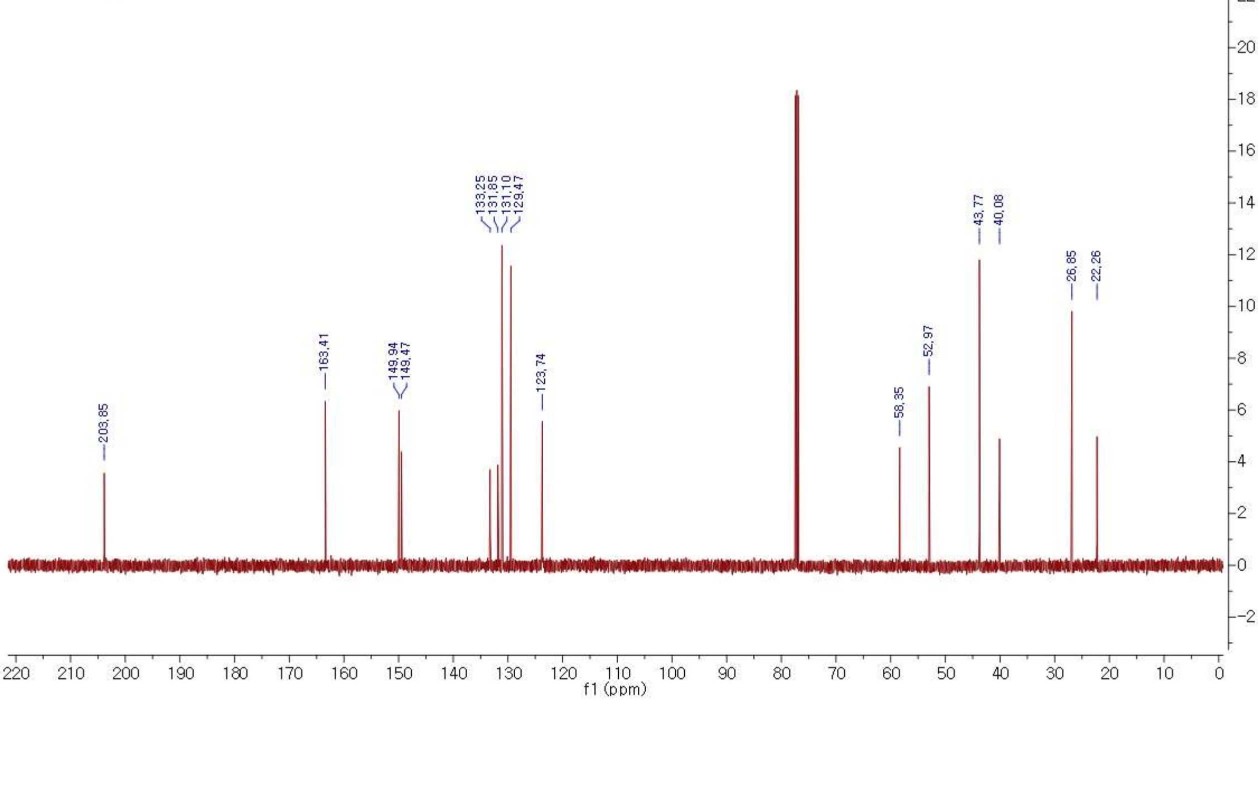

2c (LMT-359)

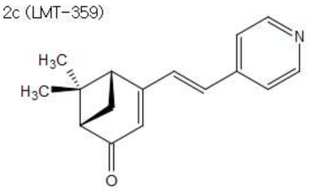

^{1}H NMR (500 MHz, cdcl$_3$) δ 8.59 (d, J = 5.9 Hz, 2H), 7.32 (d, J = 6.0 Hz, 2H), 7.10 (d, J = 16.2 Hz, 1H), 6.81 (d, J = 16.2 Hz, 1H), 5.99 (s, 1H), 3.07 (t, J = 5.8 Hz, 1H), 2.92 (dt, J = 9.5, 5.6 Hz, 1H), 2.74 (td, J = 5.7, 1.6 Hz, 1H), 2.10 (d, J = 9.5 Hz, 1H), 1.58 (s, 3H), 0.99 (s, 3H).

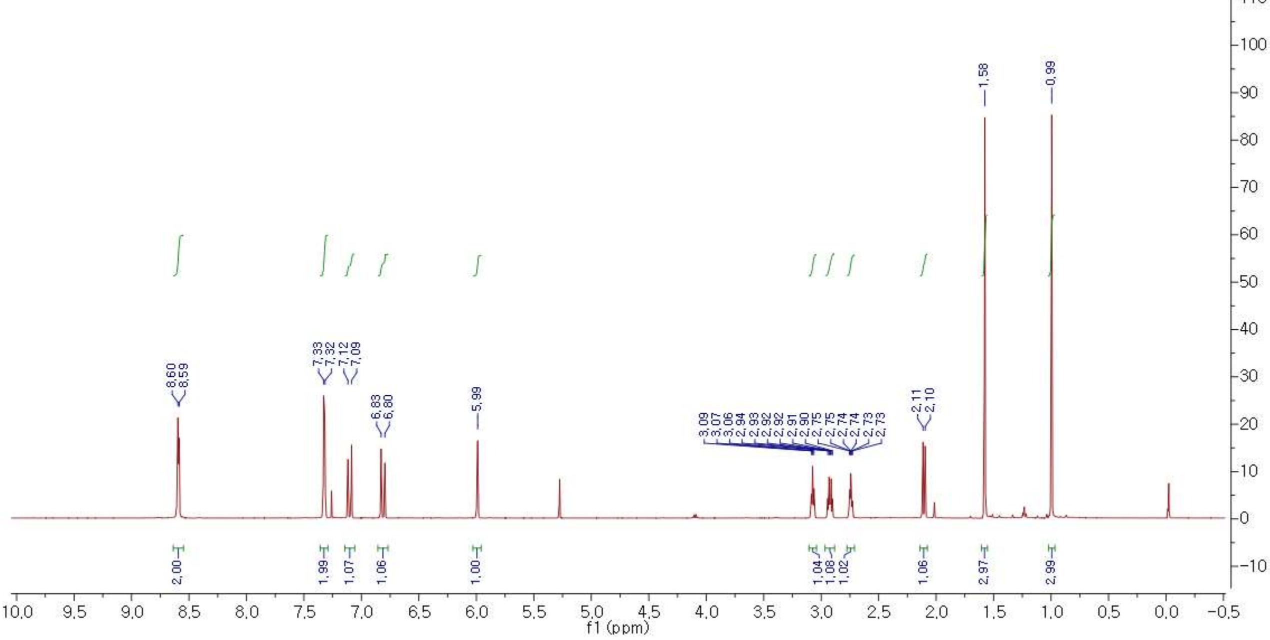

2c (LMT-359)

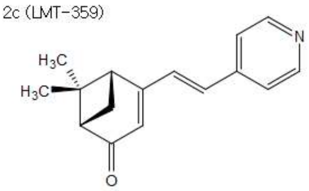

^{13}C NMR (126 MHz, cdcl$_3$) δ 203.70, 162.91, 150.55, 143.25, 132.02, 131.70, 124.75, 121.37, 58.37, 53.03, 43.82, 40.08, 26.84, 22.26.

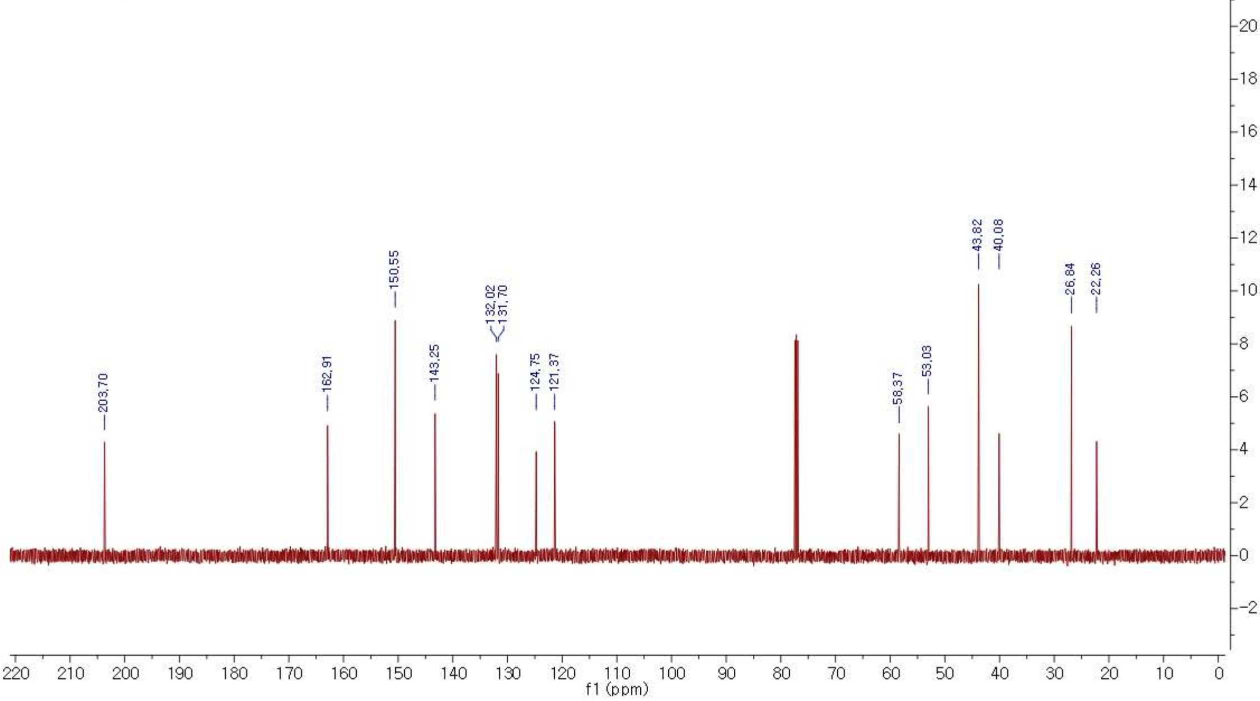

5a (LMT-459)

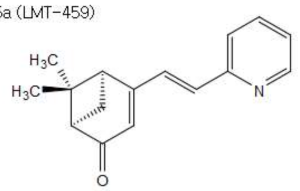

^{1}H NMR (500 MHz, cdcl$_3$) δ 8.59 (ddd, J = 4.8, 1.8, 0.8 Hz, 1H), 7.66 (td, J = 7.7, 1.8 Hz, 1H), 7.44 (d, J = 15.8 Hz, 1H), 7.37 (dt, J = 7.9, 0.9 Hz, 1H), 7.17 (ddd, J = 7.6, 4.8, 1.1 Hz, 1H), 6.95 (d, J = 15.9 Hz, 1H), 6.00 (s, 1H), 3.10 (td, J = 5.9, 1.4 Hz, 1H), 2.90 (dt, J = 9.5, 5.6 Hz, 1H), 2.72 (td, J = 5.7, 1.8 Hz, 1H), 2.10 (d, J = 9.5 Hz, 1H), 1.55 (s, 3H), 0.98 (s, 3H).

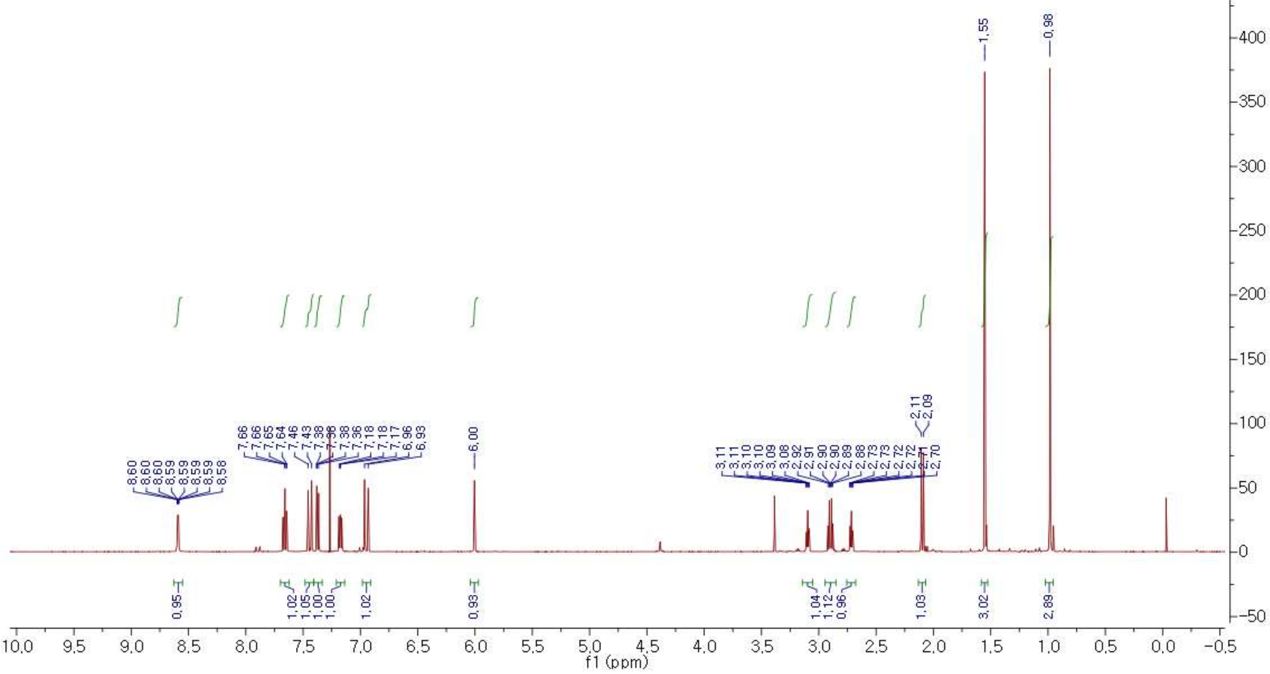

5a (LMT-459)

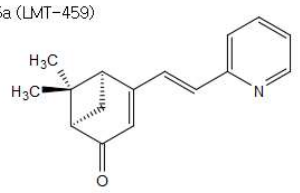

^{13}C NMR (126 MHz, cdcl$_3$) δ 204.00, 163.62, 154.41, 150.06, 136.76, 134.01, 131.22, 124.37, 123.24, 58.37, 52.99, 43.95, 40.06, 26.81, 22.22.

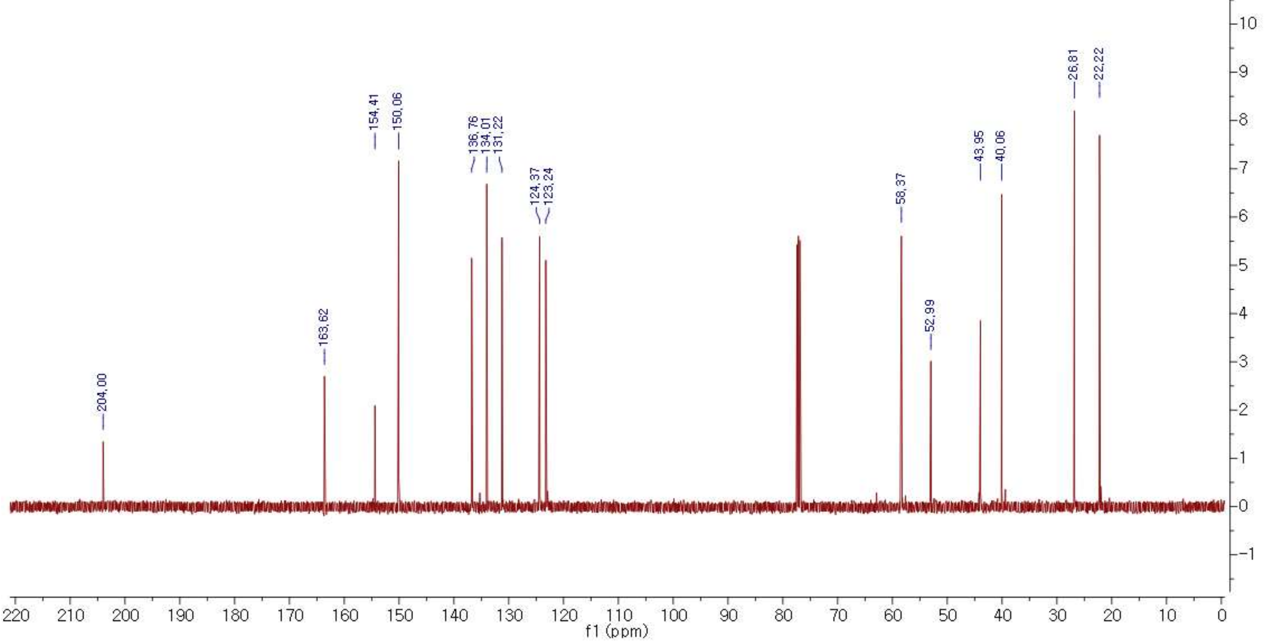

5b (LMT-458)

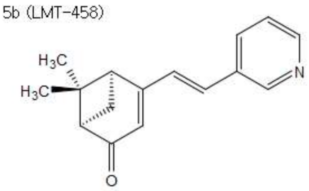

^{1}H NMR (500 MHz, cdcl$_3$) δ 8.69 (d, J = 1.9 Hz, 1H), 8.52 (dd, J = 4.7, 1.4 Hz, 1H), 7.84 (dt, J = 8.0, 1.9 Hz, 1H), 7.30 (dd, J = 8.0, 4.8 Hz, 1H), 6.95 (dd, J = 55.9, 16.2 Hz, 2H), 5.95 (s, 1H), 3.11 (td, J = 5.8, 1.3 Hz, 1H), 2.93 (dt, J = 9.5, 5.6 Hz, 1H), 2.73 (td, J = 5.7, 1.7 Hz, 1H), 2.11 (d, J = 9.5 Hz, 1H), 1.58 (s, 3H), 1.01 (s, 3H).

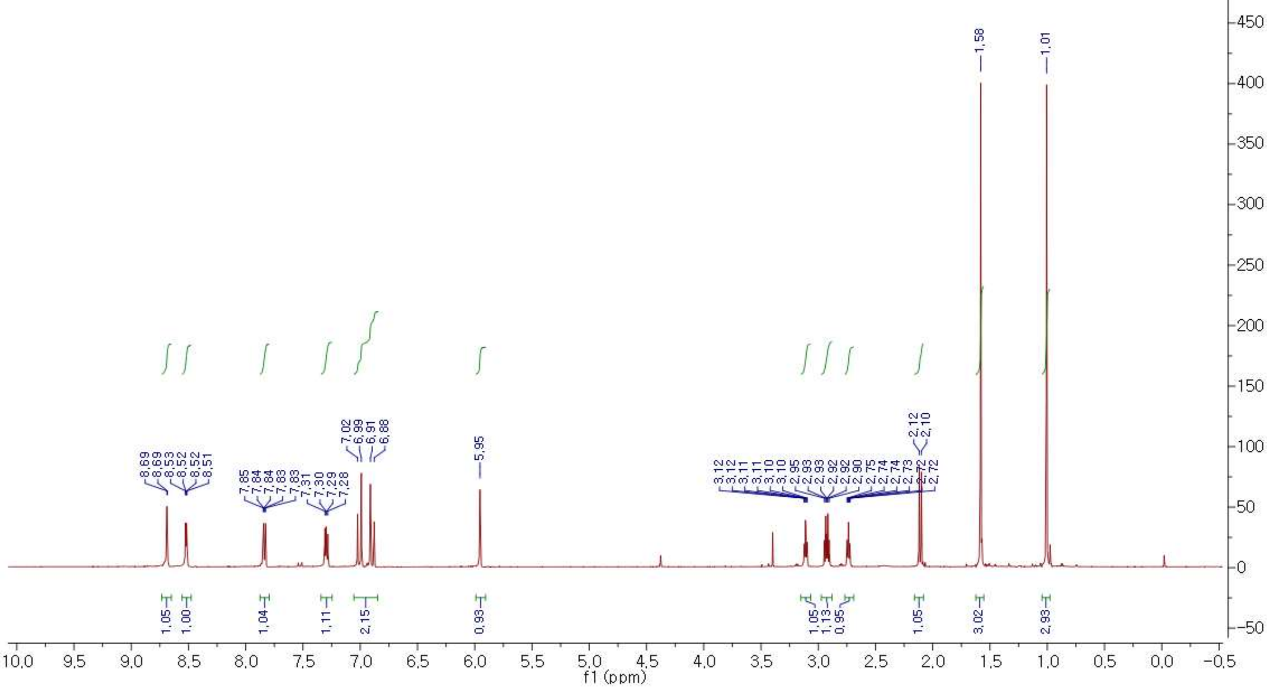

5b (LMT-458)

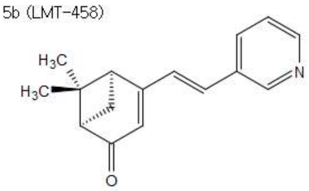

^{13}C NMR (126 MHz, cdcl$_3$) δ 203.74, 163.36, 149.83, 149.36, 133.21, 131.78, 131.05, 129.37, 123.70, 123.60, 58.26, 52.87, 43.68, 40.00, 26.76, 22.17.

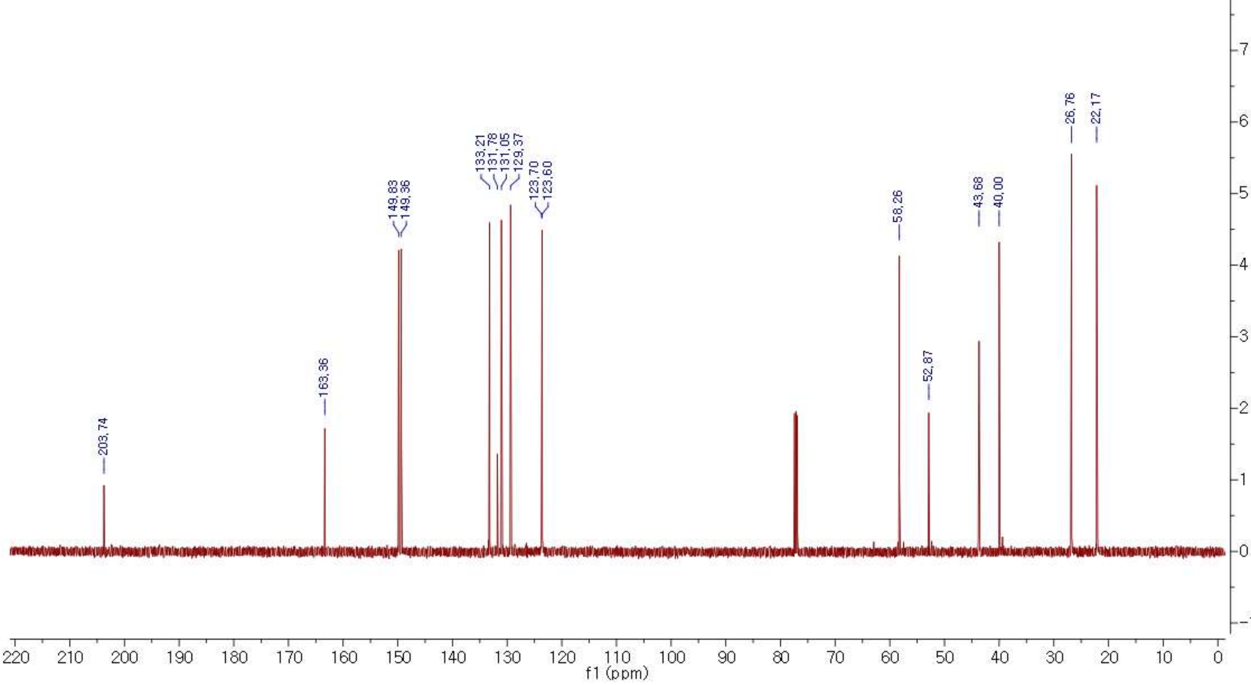

5c (LMT-457)

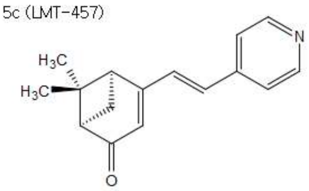

^{1}H NMR (500 MHz, cdcl$_3$) δ 8.60 (d, J = 5.8 Hz, 2H), 7.33 (dd, J = 4.6, 1.6 Hz, 2H), 7.11 (d, J = 16.1 Hz, 1H), 6.82 (d, J = 16.2 Hz, 1H), 6.00 (s, 1H), 3.08 (td, J = 5.8, 1.4 Hz, 1H), 2.93 (dt, J = 9.5, 5.6 Hz, 1H), 2.75 (td, J = 5.7, 1.8 Hz, 1H), 2.11 (d, J = 9.5 Hz, 1H), 1.58 (s, 3H), 1.00 (s, 3H).

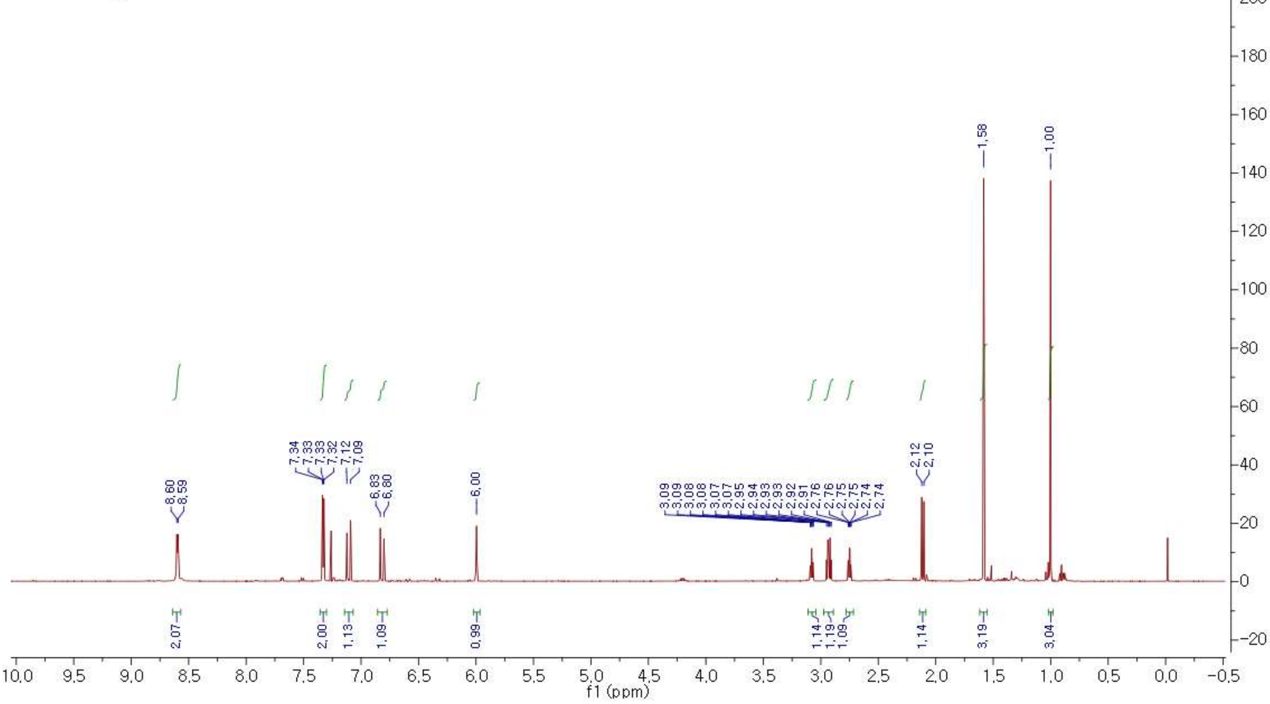

5c (LMT-457)

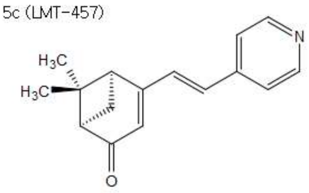

^{13}C NMR (126 MHz, cdcl$_3$) δ 203.73, 162.93, 150.54, 143.31, 132.03, 131.74, 124.80, 121.38, 58.39, 53.06, 43.85, 40.11, 26.85, 22.27.

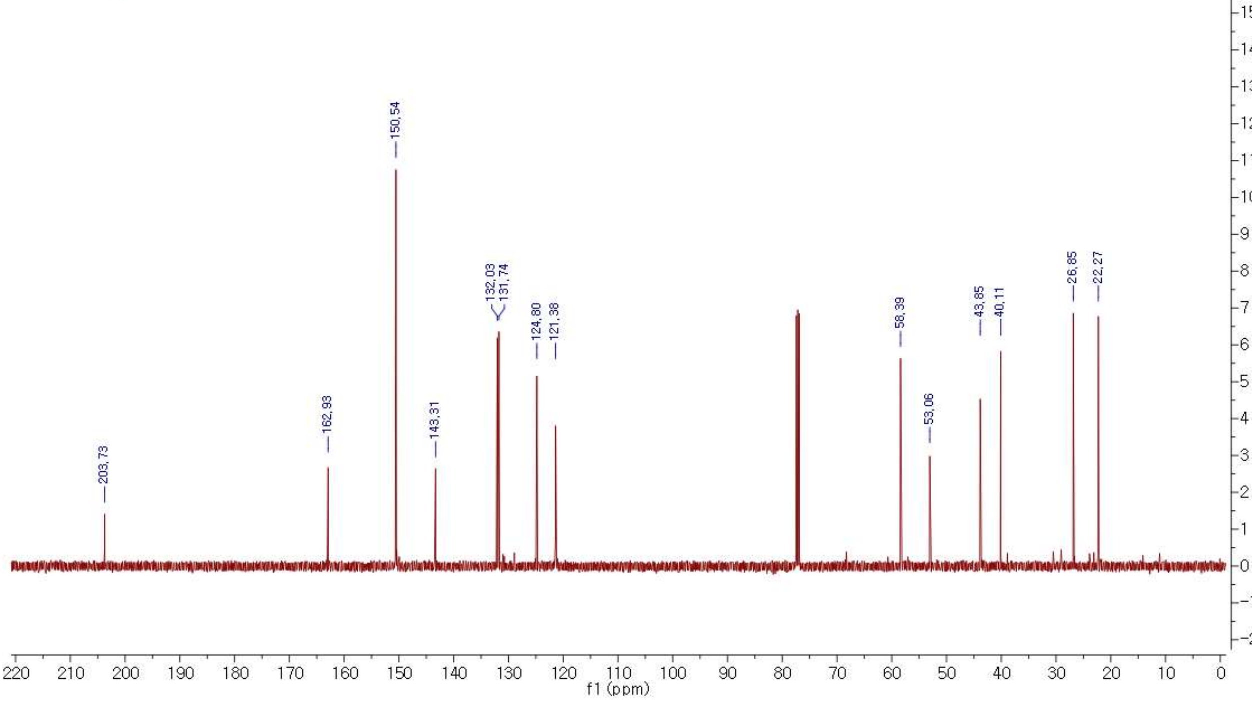

5d (LMT-460)

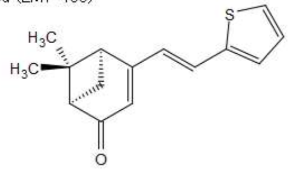

^{1}H NMR (500 MHz, cdcl$_3$) δ 7.28 (d, J = 5.0 Hz, 1H), 7.13 (d, J = 3.6 Hz, 1H), 7.04 (d, J = 15.9 Hz, 1H), 7.00 (dd, J = 5.1, 3.6 Hz, 1H), 6.72 (d, J = 15.9 Hz, 1H), 5.86 (s, 1H), 3.02 (td, J = 5.9, 1.2 Hz, 1H), 2.87 (dt, J = 9.4, 5.6 Hz, 1H), 2.69 (td, J = 5.7, 1.7 Hz, 1H), 2.07 (d, J = 9.4 Hz, 1H), 1.54 (s, 3H), 0.98 (s, 3H).

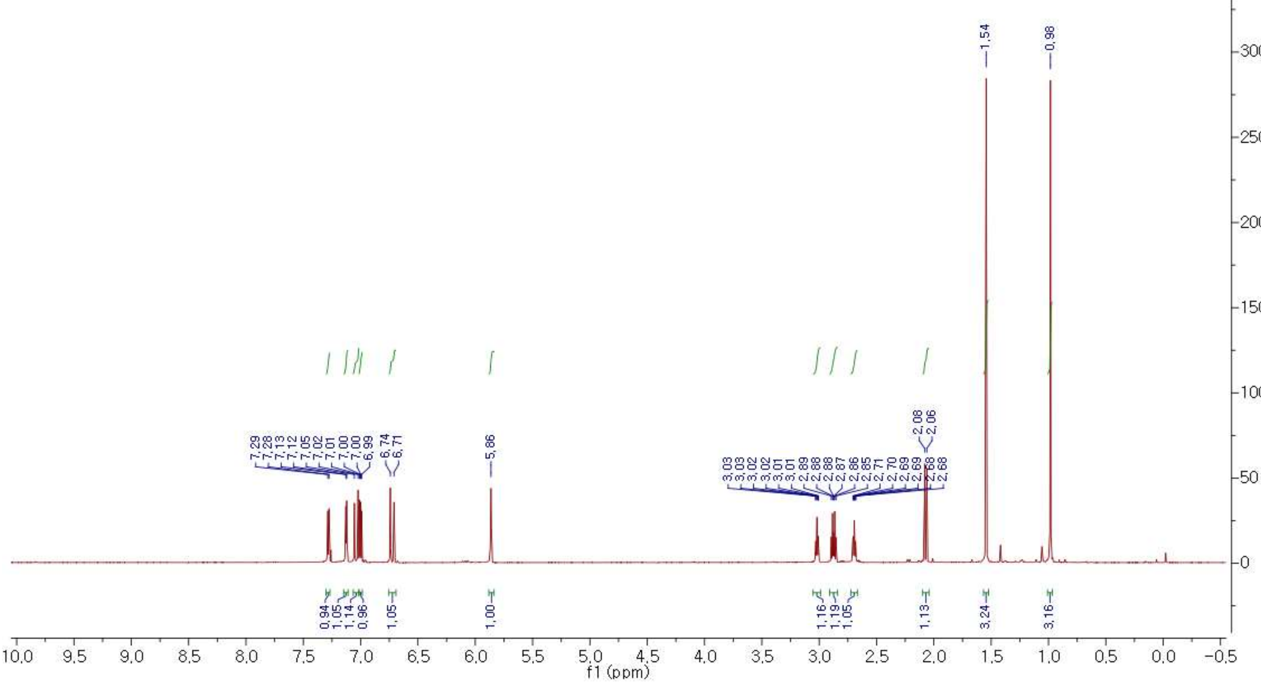

5d (LMT-460)

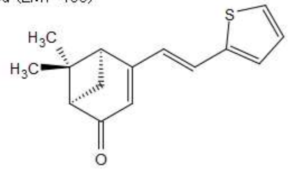

^{13}C NMR (126 MHz, cdcl$_3$) δ 203.86, 163.85, 141.76, 128.72, 128.06, 127.85, 127.04, 126.80, 122.18, 58.22, 52.74, 43.80, 39.95, 26.80, 22.20.

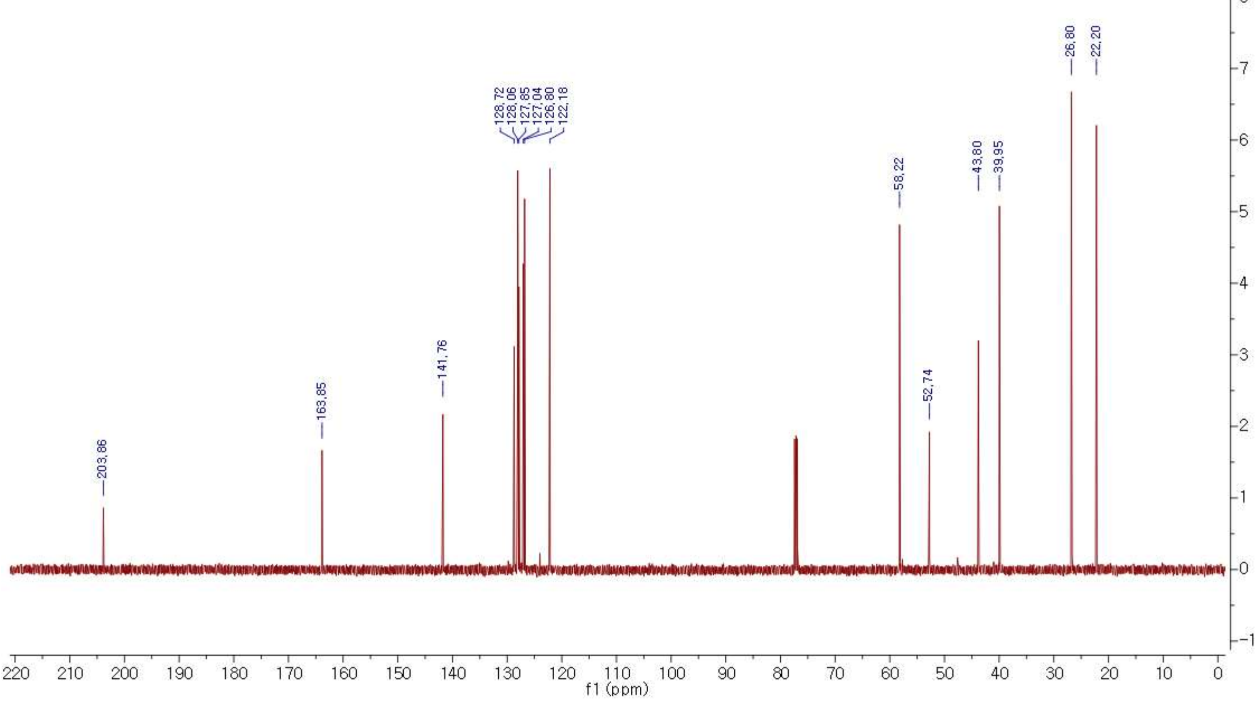

5e (LMT-462)

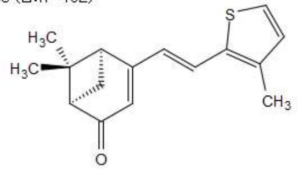

^{1}H NMR (500 MHz, cdcl$_3$) δ 7.17 (d, J = 5.1 Hz, 1H), 7.05 (d, J = 15.8 Hz, 1H), 6.81 (dd, J = 5.1, 1.6 Hz, 1H), 6.65 (d, J = 15.8 Hz, 1H), 5.84 (s, 1H), 3.05 (t, J = 5.8 Hz, 1H), 2.91 – 2.85 (m, 1H), 2.68 (td, J = 5.7, 1.7 Hz, 1H), 2.29 (s, 3H), 2.08 (dd, J = 8.9, 2.1 Hz, 1H), 1.55 (s, 3H), 0.99 (s, 3H).

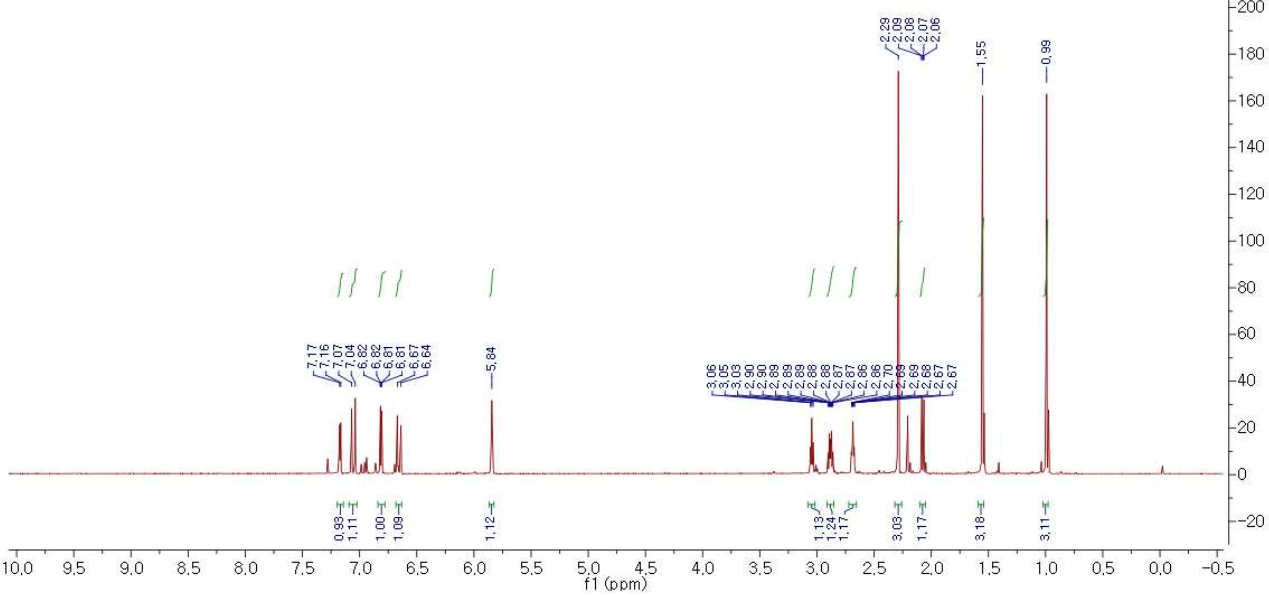

5e (LMT-462)

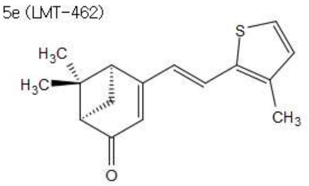

^{13}C NMR (126 MHz, cdcl$_3$) δ 203.74, 164.08, 138.70, 135.63, 131.09, 126.15, 125.96, 125.61, 121.58, 58.11, 52.61, 43.81, 39.88, 26.75, 22.11, 14.11.

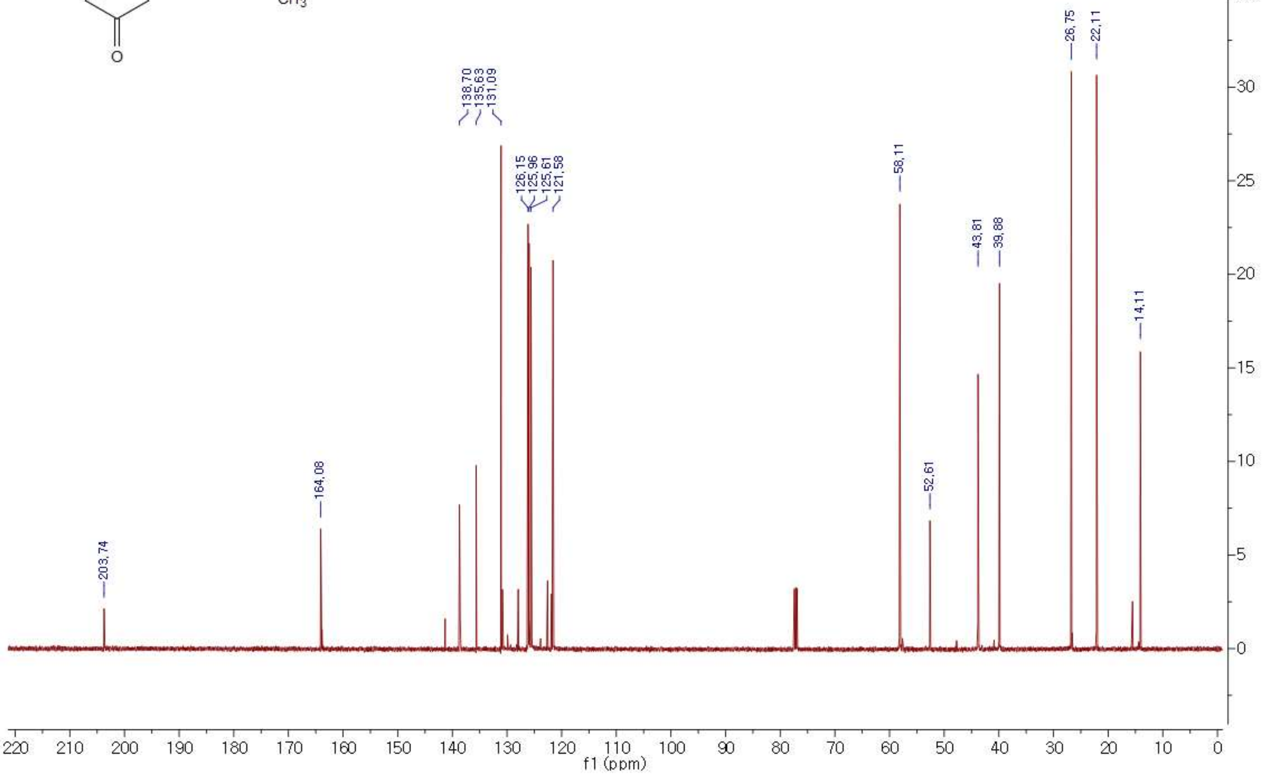

5f (LMT-465)

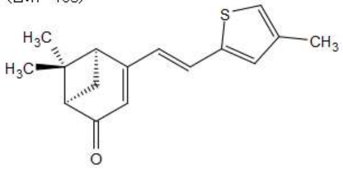

^{1}H NMR (500 MHz, cdcl$_3$) δ 6.98 (d, J = 15.9 Hz, 1H), 6.96 (d, J = 0.7 Hz, 1H), 6.89 (s, 1H), 6.71 (d, J = 15.8 Hz, 1H), 5.87 (s, 1H), 3.02 (td, J = 5.9, 1.4 Hz, 1H), 2.89 (dt, J = 9.4, 5.6 Hz, 1H), 2.71 (td, J = 5.7, 1.8 Hz, 1H), 2.23 (s, 3H), 2.09 (d, J = 9.4 Hz, 1H), 1.56 (s, 3H), 1.00 (s, 3H).

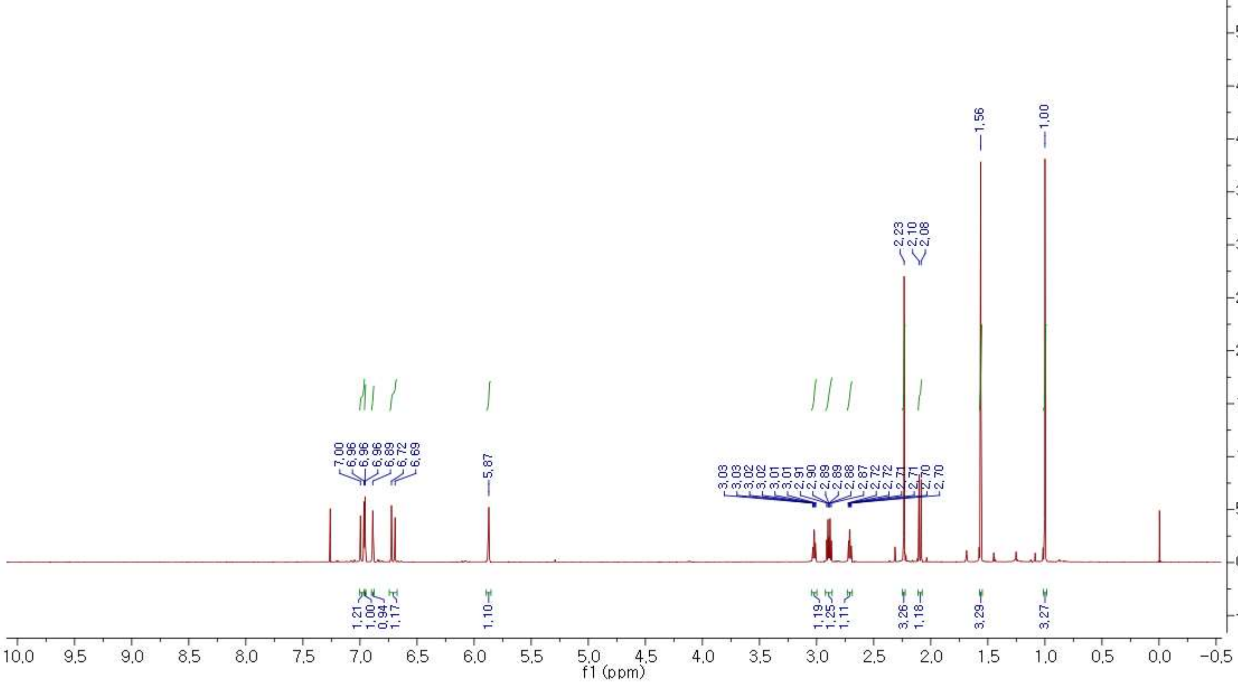

5f (LMT-465)

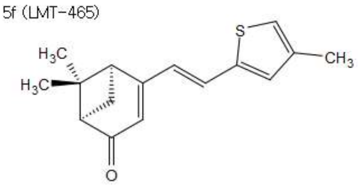

^{13}C NMR (126 MHz, cdcl$_3$) δ 204.00, 164.00, 141.54, 138.79, 130.92, 128.03, 126.53, 122.72, 122.12, 58.31, 52.83, 43.92, 40.03, 26.89, 22.28, 15.70.

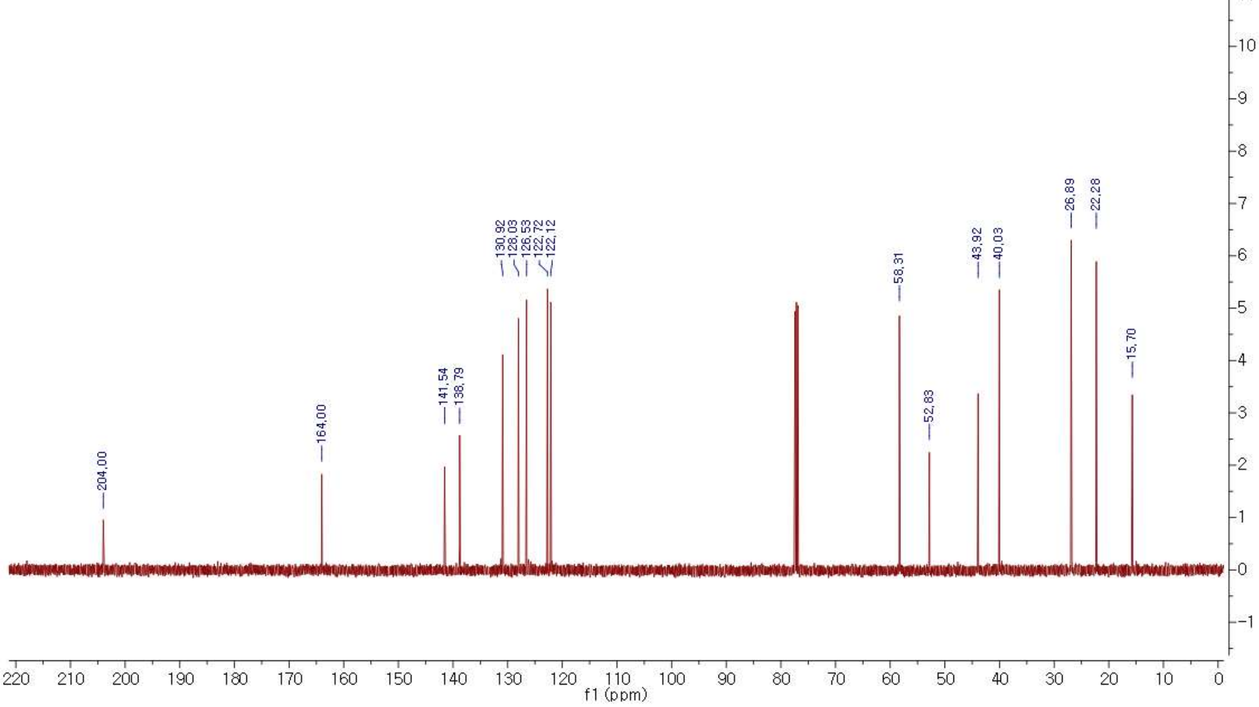

5g (LMT-463)

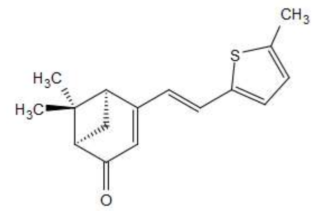

^{1}H NMR (500 MHz, cdcl$_3$) δ 7.16 (d, J = 5.1 Hz, 1H), 7.06 (d, J = 15.7 Hz, 1H), 6.81 (d, J = 5.1 Hz, 1H), 6.65 (d, J = 15.7 Hz, 1H), 5.83 (s, 1H), 3.05 (td, J = 5.9, 1.3 Hz, 1H), 2.88 (dt, J = 9.3, 5.6 Hz, 1H), 2.68 (td, J = 5.7, 1.8 Hz, 1H), 2.28 (s, 3H), 2.06 (d, J = 9.3 Hz, 1H), 1.55 (s, 3H), 0.99 (s, 3H).

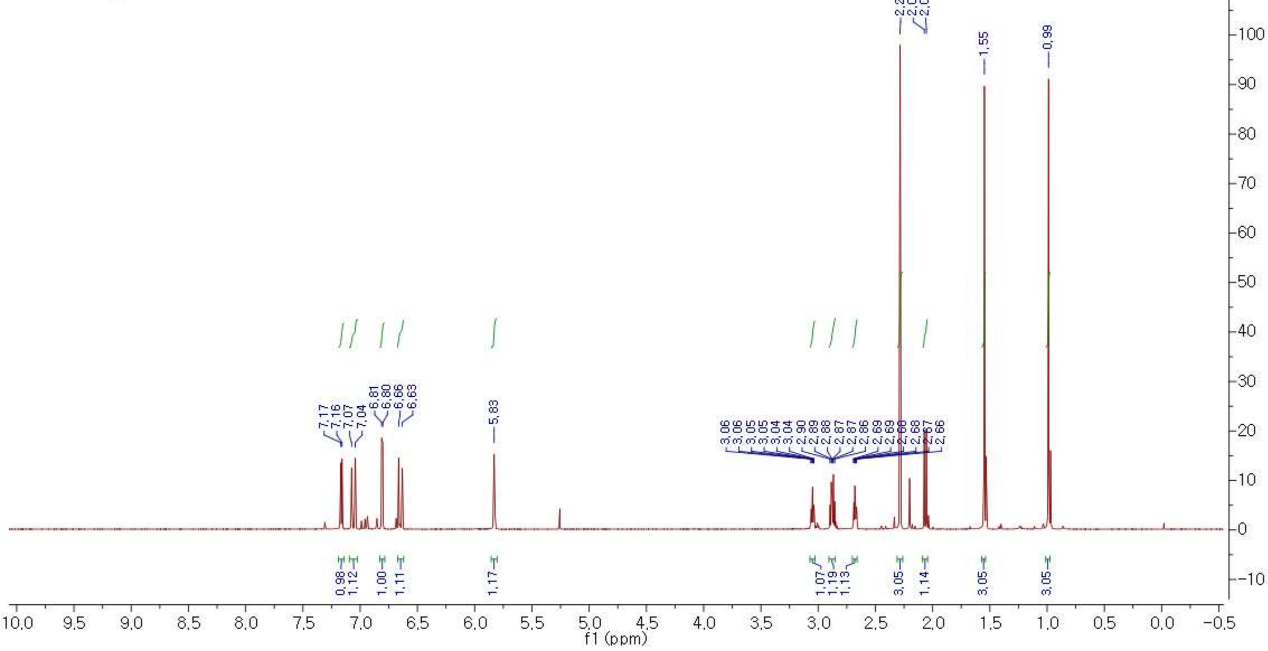

5g (LMT-463)

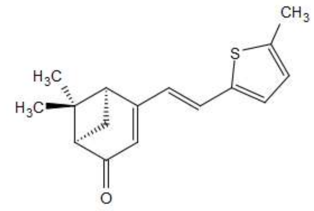

^{13}C NMR (126 MHz, cdcl$_3$) δ 203.47, 163.93, 138.59, 135.51, 130.98, 126.05, 125.81, 125.50, 121.44, 57.98, 52.43, 43.66, 39.74, 26.63, 22.00, 14.00.

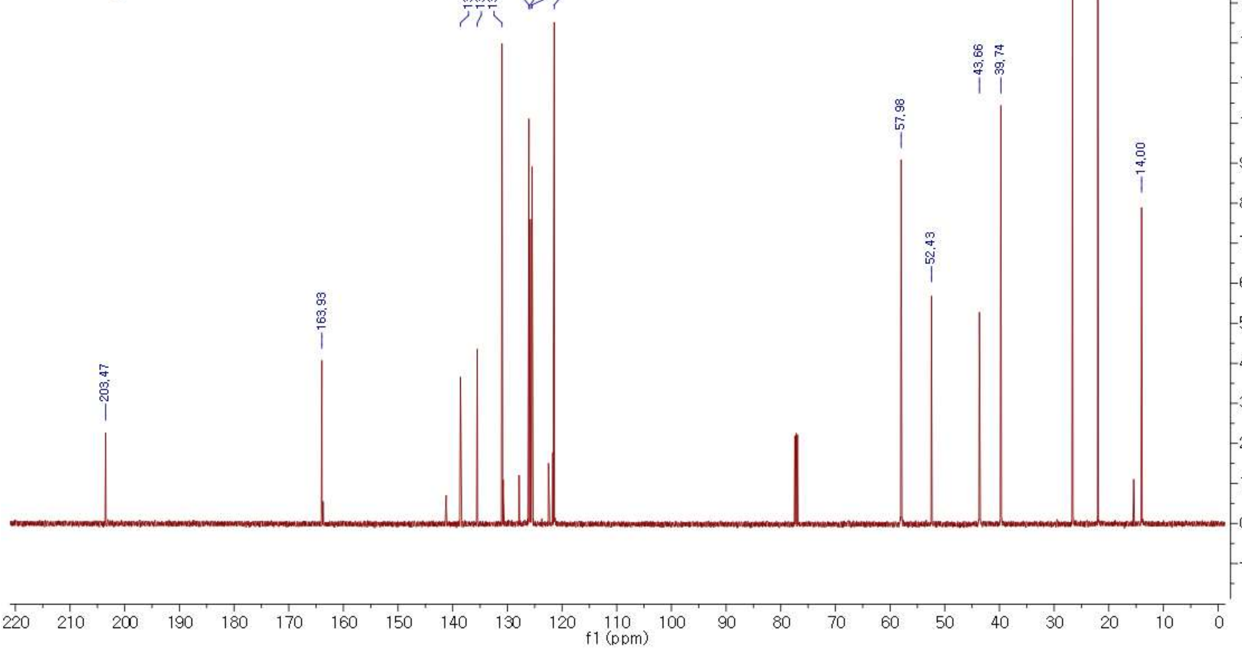

6a (LMT-385)

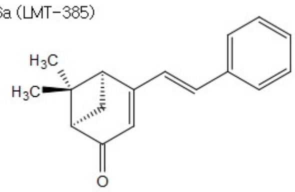

^{1}H NMR (500 MHz, cdcl$_3$) δ 7.52 – 7.48 (m, 2H), 7.39 – 7.35 (m, 2H), 7.34 – 7.30 (m, 1H), 6.94 (q, J = 16.2 Hz, 2H), 5.93 (s, 1H), 3.12 (t, J = 5.8 Hz, 1H), 2.92 (dt, J = 9.5, 5.6 Hz, 1H), 2.73 (td, J = 5.7, 1.5 Hz, 1H), 2.12 (d, J = 9.4 Hz, 1H), 1.58 (s, 3H), 1.01 (s, 3H).

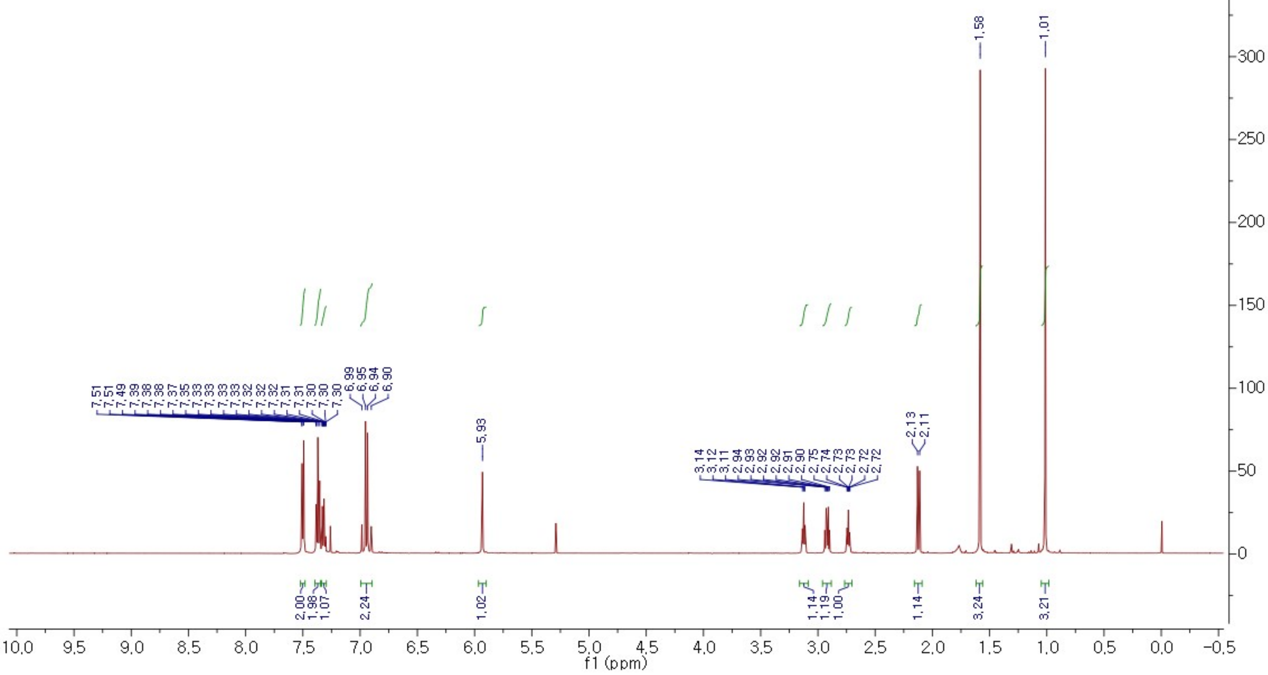

6a (LMT-385)

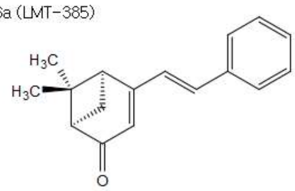

^{13}C NMR (126 MHz, cdcl$_3$) δ 204.36, 164.41, 136.04, 135.12, 129.28, 128.99, 127.47, 122.76, 58.29, 53.03, 43.76, 40.16, 26.88, 22.27.

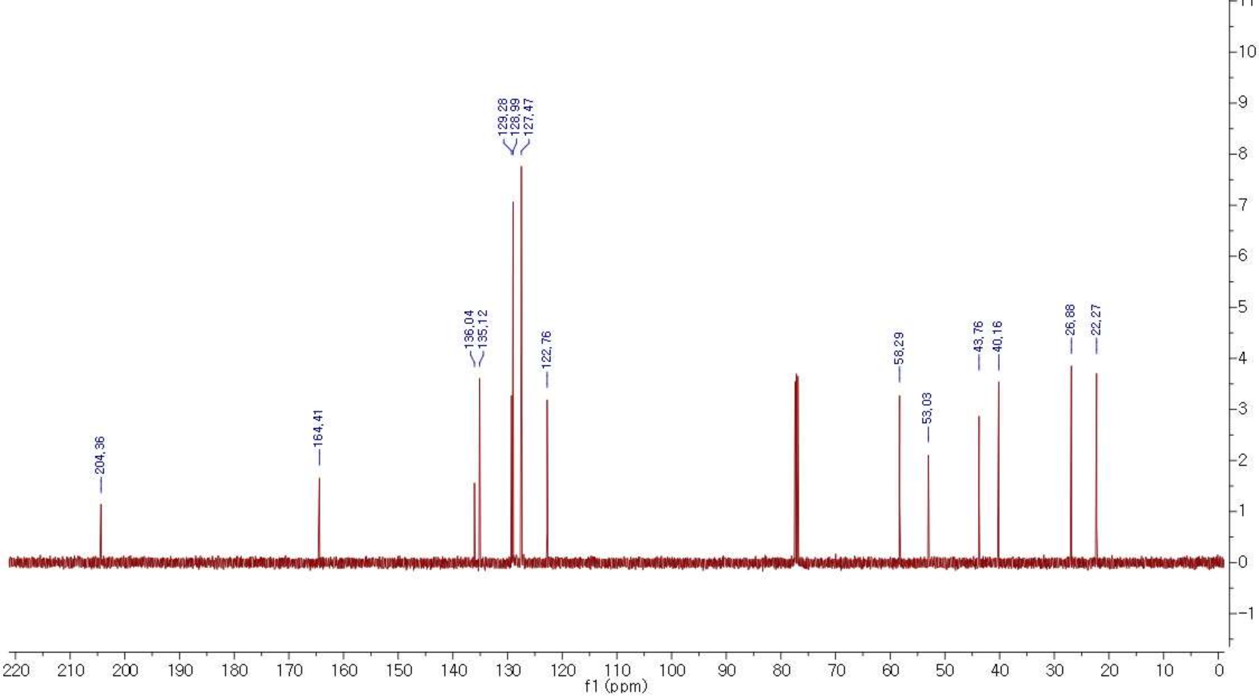

6b (LMT-407)

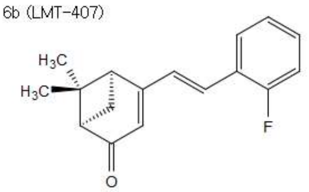

^1H NMR (500 MHz, cdcl$_3$) δ 7.58 (t, J = 7.2 Hz, 1H), 7.32 – 7.21 (m, 1H), 7.18 – 6.94 (m, 4H), 5.93 (s, 1H), 3.18 – 3.03 (m, 1H), 2.96 – 2.82 (m, 1H), 2.76 – 2.65 (m, 1H), 2.09 (d, J = 9.4 Hz, 1H), 1.56 (s, 3H), 1.00 (s, 3H).

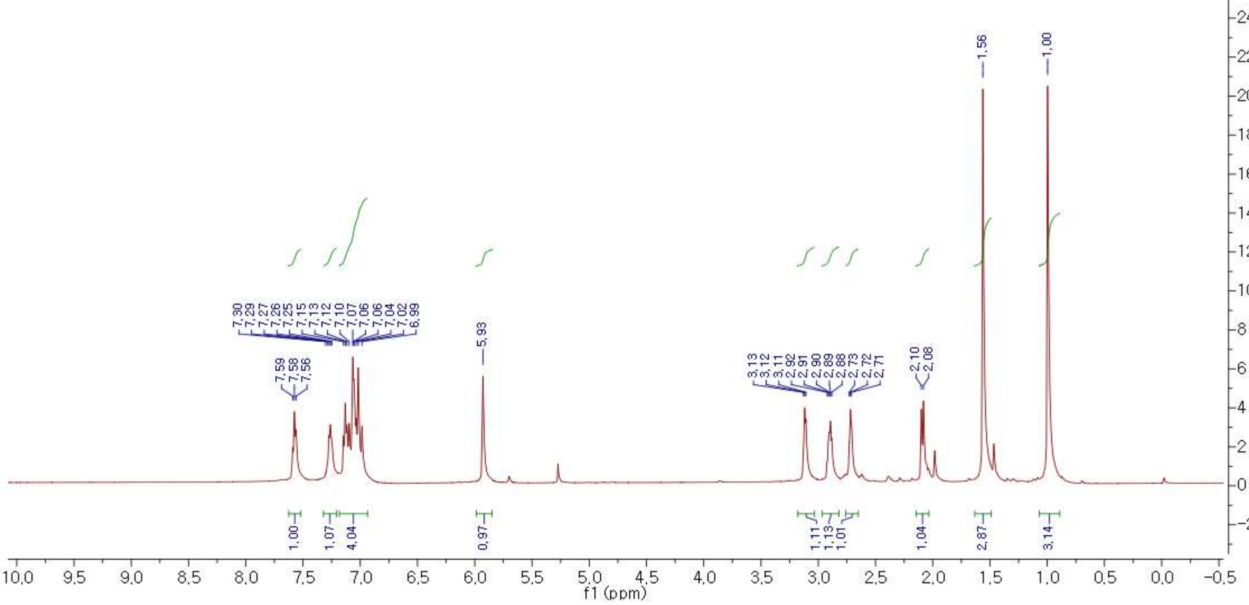

6b (LMT-407)

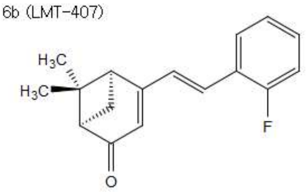

^{13}C NMR (126 MHz, cdcl$_3$) δ 203.92, 164.00, 161.66, 130.37, 129.22, 127.38, 126.79, 124.37, 123.81, 123.19, 116.00, 58.12, 52.78, 43.35, 39.93, 26.65, 22.05.

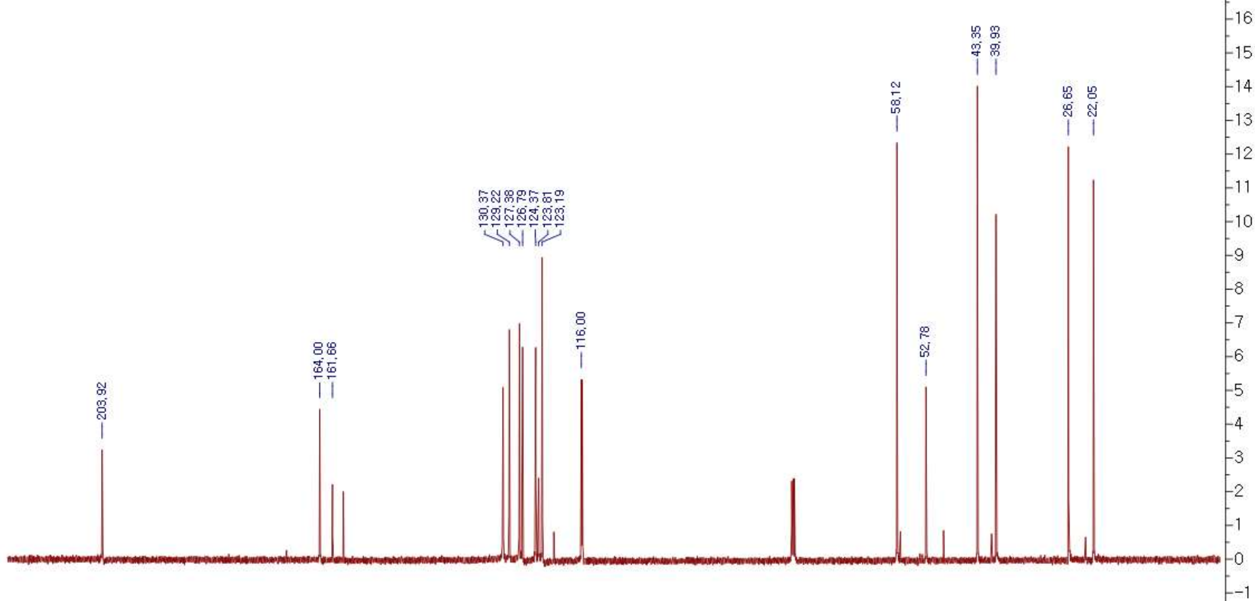

6c (LMT-406)

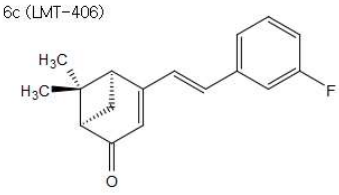

^{1}H NMR (500 MHz, cdcl$_3$) δ 7.47 – 7.12 (m, 3H), 7.07 – 6.79 (m, 3H), 5.93 (s, 1H), 3.09 (s, 1H), 3.00 – 2.84 (m, 1H), 2.72 (s, 1H), 2.10 (d, J = 9.3 Hz, 1H), 1.57 (s, 3H), 1.00 (s, 3H).

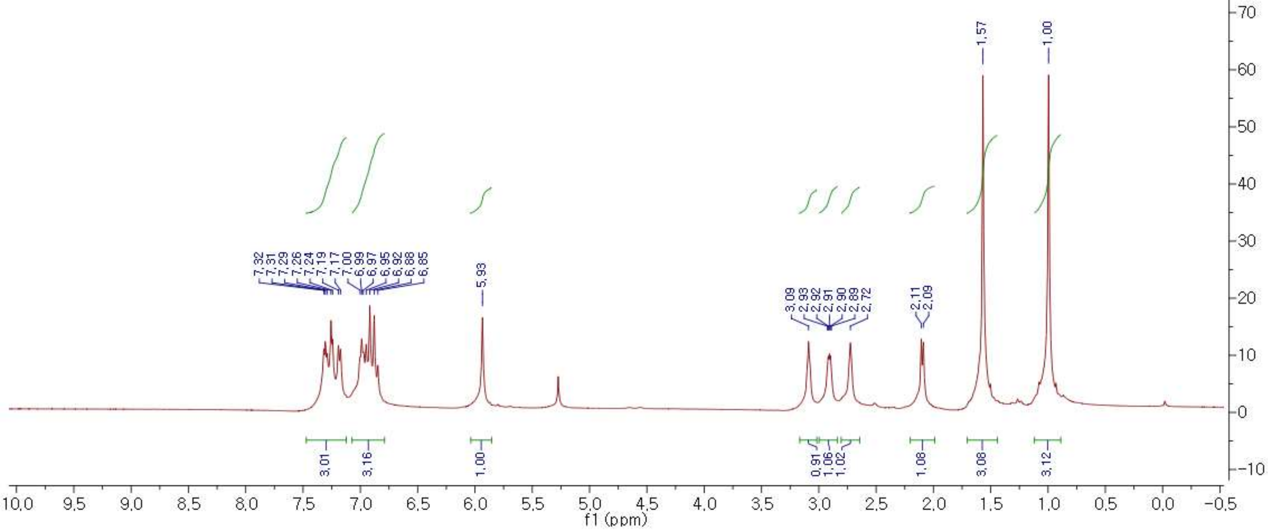

6c (LMT-406)

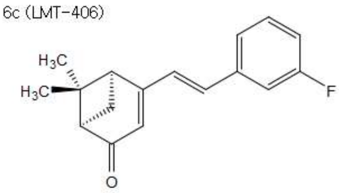

^{13}C NMR (126 MHz, cdcl$_3$) δ 203.94, 163.68, 162.05, 138.30, 133.56, 130.38, 128.60, 123.30, 115.80, 113.64, 58.16, 52.87, 43.58, 39.98, 26.71, 22.12.

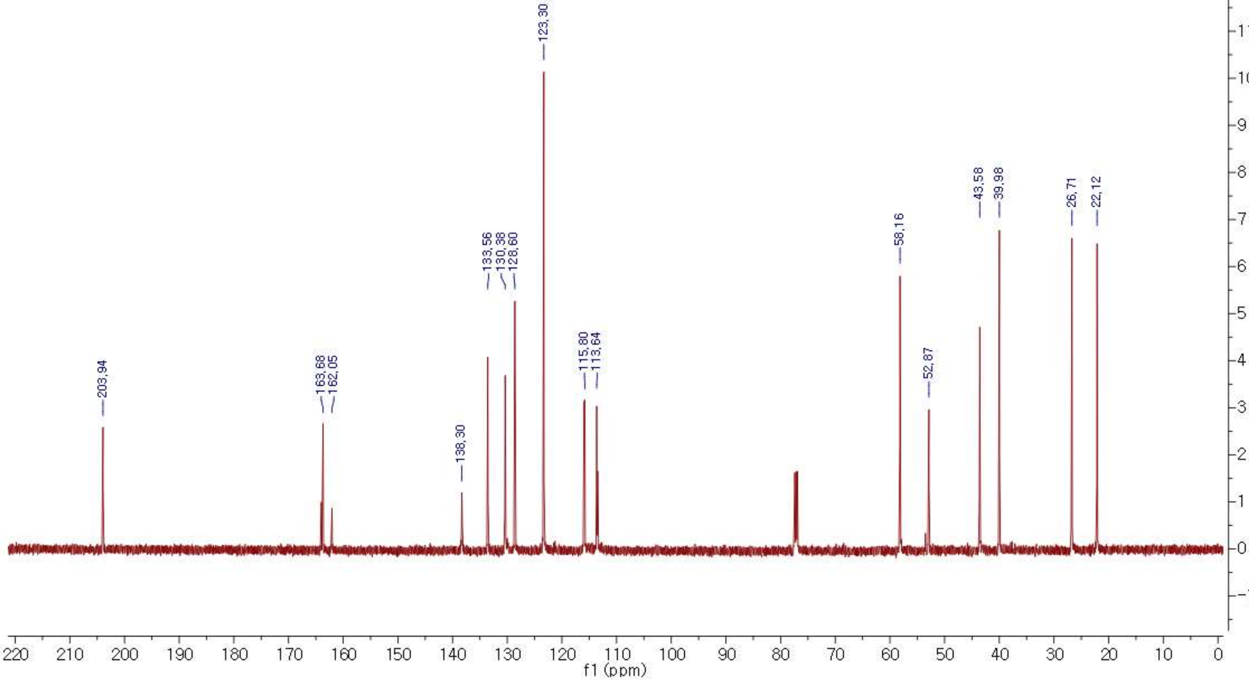

6d (LMT-405)

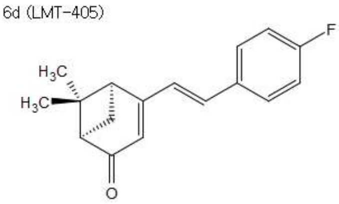

^{1}H NMR (500 MHz, cdcl$_3$) δ 7.54 – 7.38 (m, 2H), 7.10 – 6.97 (m, 2H), 6.92 – 6.79 (m, 2H), 5.91 (s, 1H), 3.10 (t, J = 5.6 Hz, 1H), 2.90 (dt, J = 9.4, 5.6 Hz, 1H), 2.75 – 2.65 (m, 1H), 2.09 (d, J = 9.4 Hz, 1H), 1.56 (s, 3H), 1.00 (s, 3H).

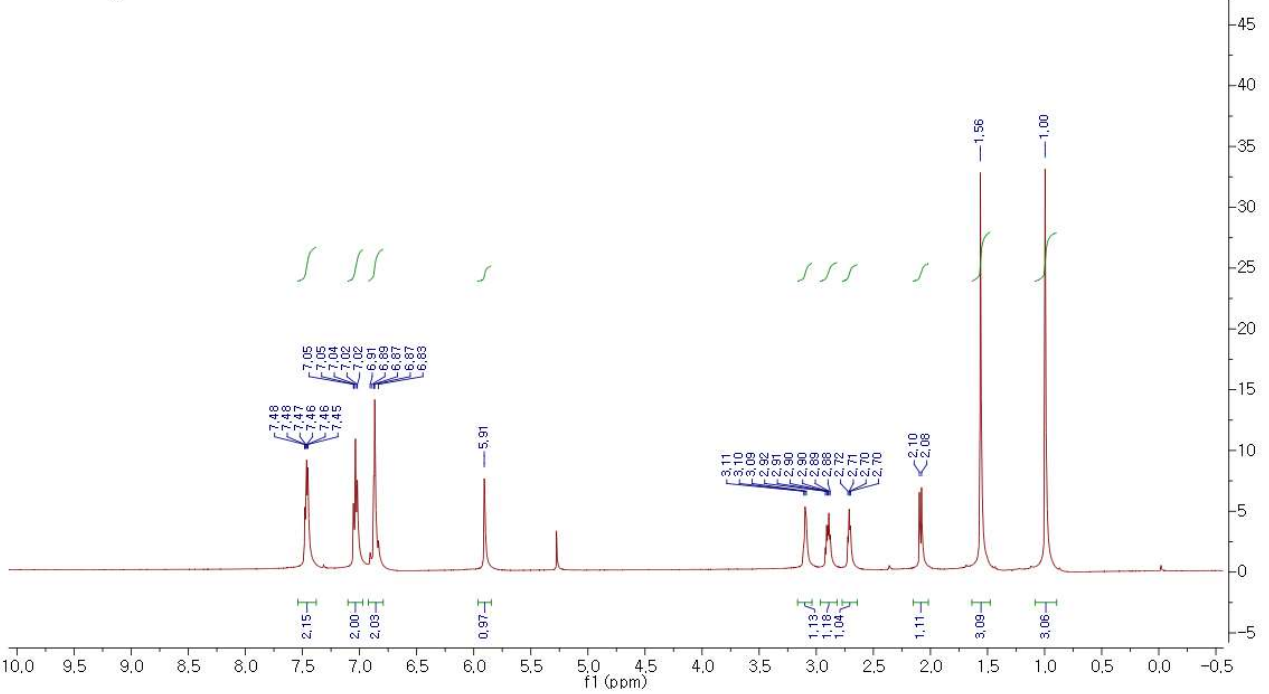

6d (LMT-405)

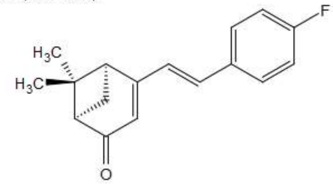

^{13}C NMR (126 MHz, cdcl$_3$) δ 203.88, 163.99, 161.96, 133.57, 132.15, 129.01, 128.95, 127.00, 122.49, 115.90, 115.73, 58.04, 52.71, 43.47, 39.88, 26.61, 22.02.

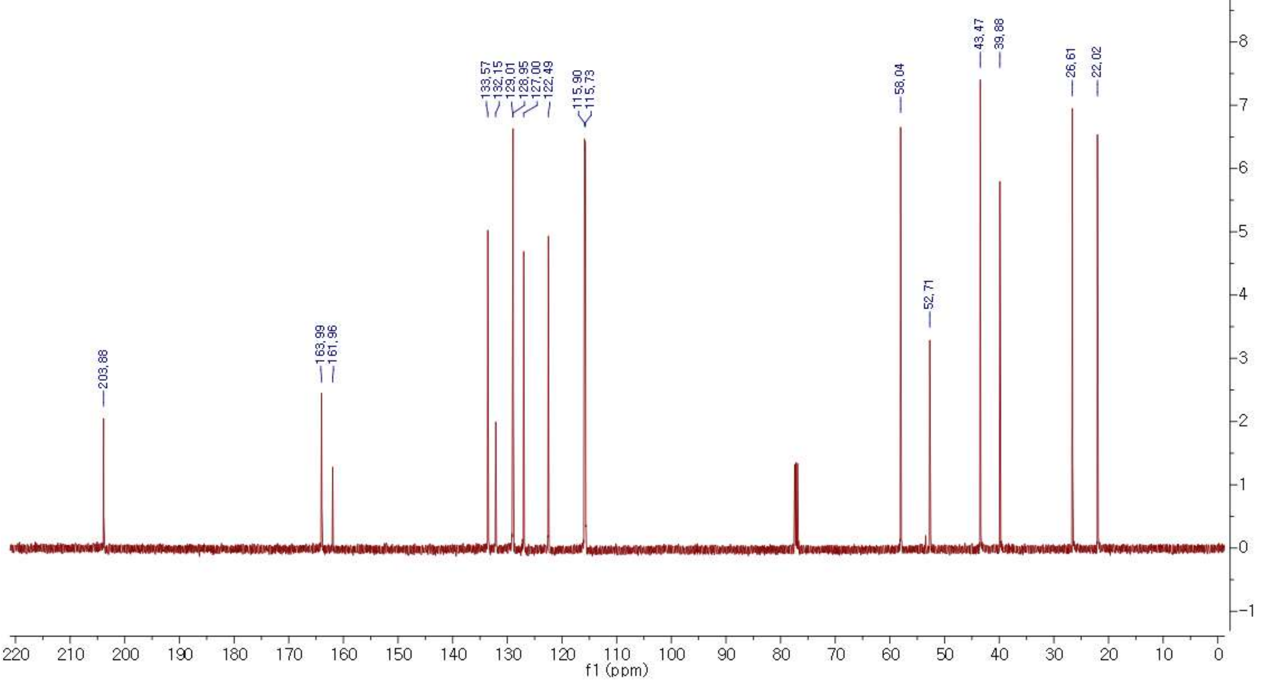

6e (LMT-419)

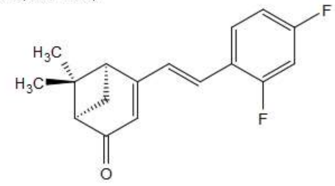

^{1}H NMR (500 MHz, cdcl$_3$) δ 7.55 (td, J = 8.6, 6.4 Hz, 1H), 6.95 (q, J = 16.4 Hz, 2H), 6.90 – 6.84 (m, 1H), 6.81 (ddd, J = 11.1, 8.7, 2.5 Hz, 1H), 5.91 (s, 1H), 3.08 (td, J = 5.8, 1.4 Hz, 1H), 2.90 (dt, J = 9.4, 5.6 Hz, 1H), 2.71 (td, J = 5.7, 1.8 Hz, 1H), 2.08 (d, J = 9.4 Hz, 1H), 1.56 (s, 3H), 0.99 (s, 3H).

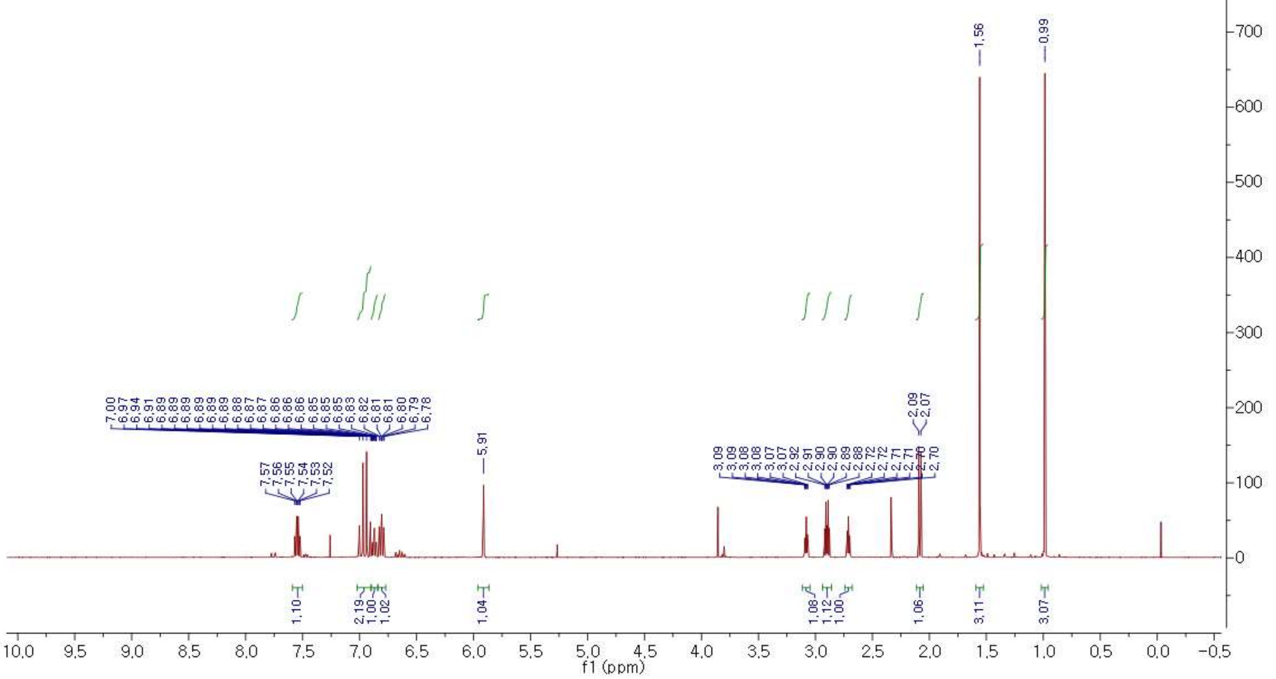

6e (LMT-419)

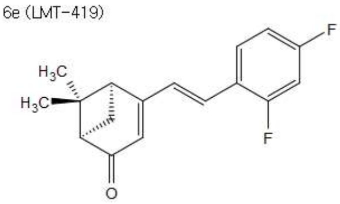

^{13}C NMR (126 MHz, cdcl$_3$) δ 203.88, 163.88, 162.01, 159.89, 129.15, 128.58, 125.89, 123.37, 120.57, 112.00, 104.44, 58.32, 52.88, 43.67, 40.05, 26.80, 22.19.

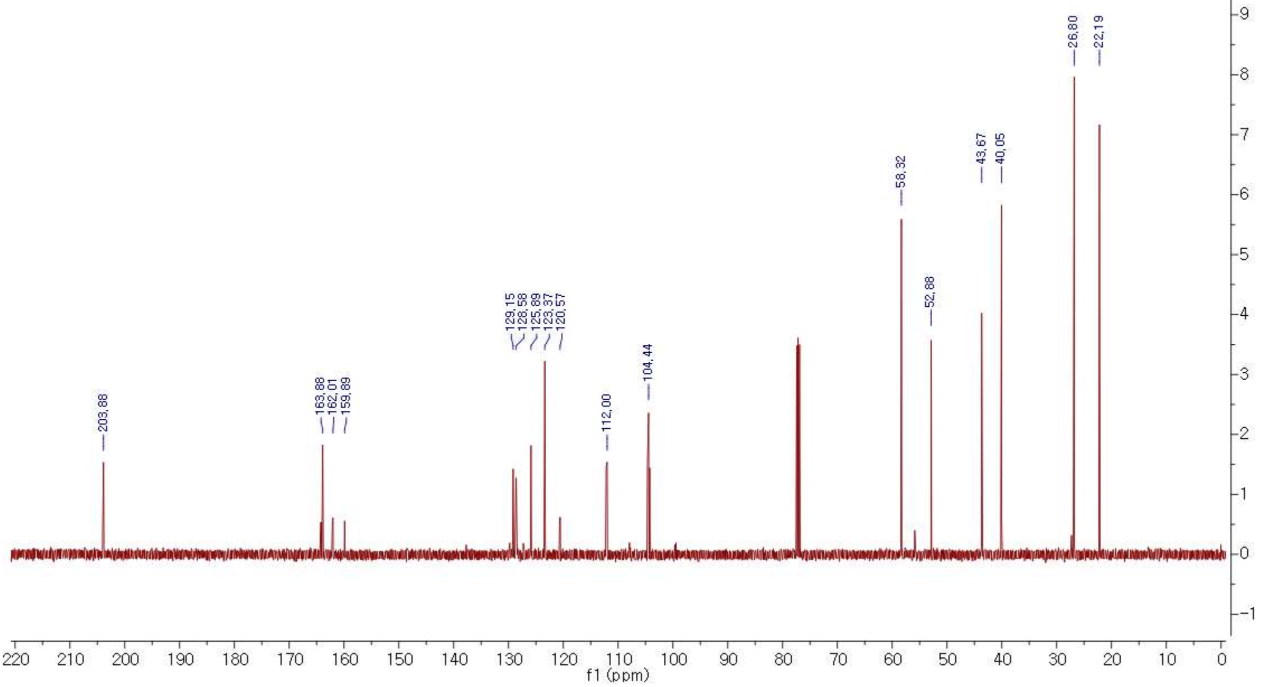

6f (LMT-418)

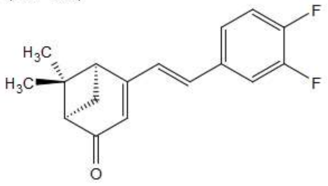

^{1}H NMR (500 MHz, cdcl$_3$) δ 7.23 (dd, J = 12.3, 2.1 Hz, 1H), 7.17 (d, J = 8.6 Hz, 1H), 6.92 (t, J = 8.5 Hz, 1H), 6.86 – 6.69 (m, 2H), 5.87 (s, 1H), 3.06 (t, J = 5.4 Hz, 1H), 2.88 (dt, J = 9.4, 5.6 Hz, 1H), 2.69 (td, J = 5.7, 1.6 Hz, 1H), 2.07 (d, J = 9.4 Hz, 1H), 1.55 (s, 3H), 0.98 (s, 3H).

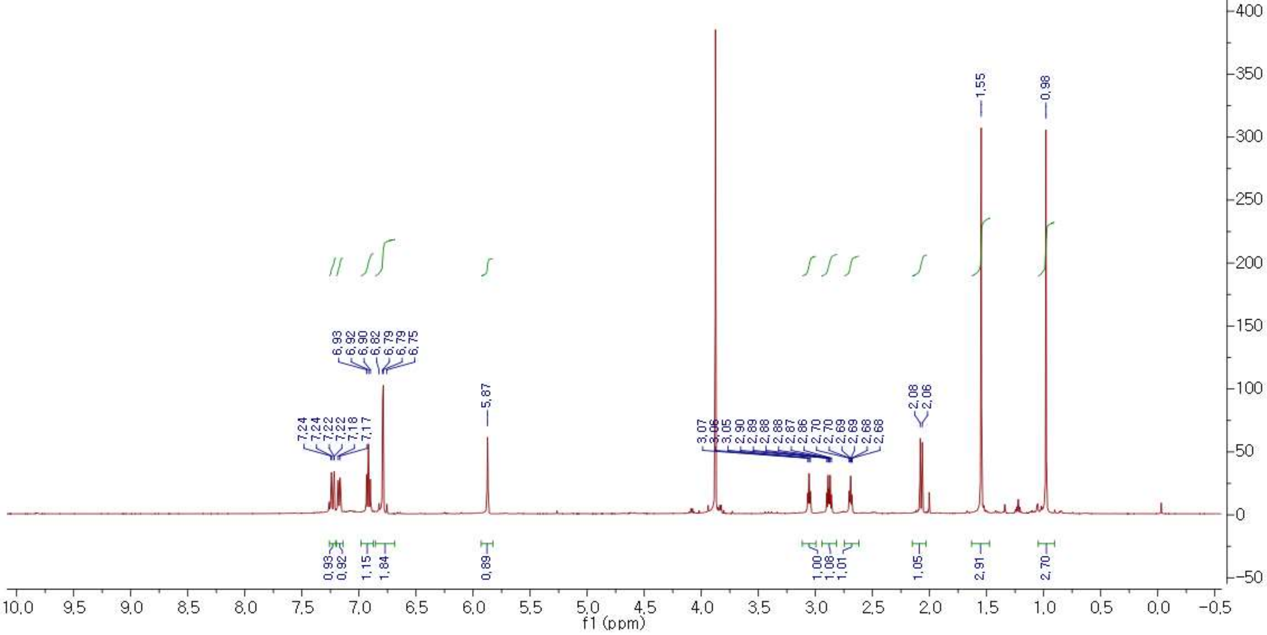

6f (LMT-418)

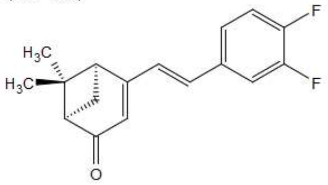

^{13}C NMR (126 MHz, cdcl$_3$) δ 203.99, 164.14, 151.58, 148.53, 133.59, 129.53, 126.58, 124.26, 122.36, 114.28, 113.44, 58.26, 52.81, 43.86, 40.02, 26.80, 22.20.

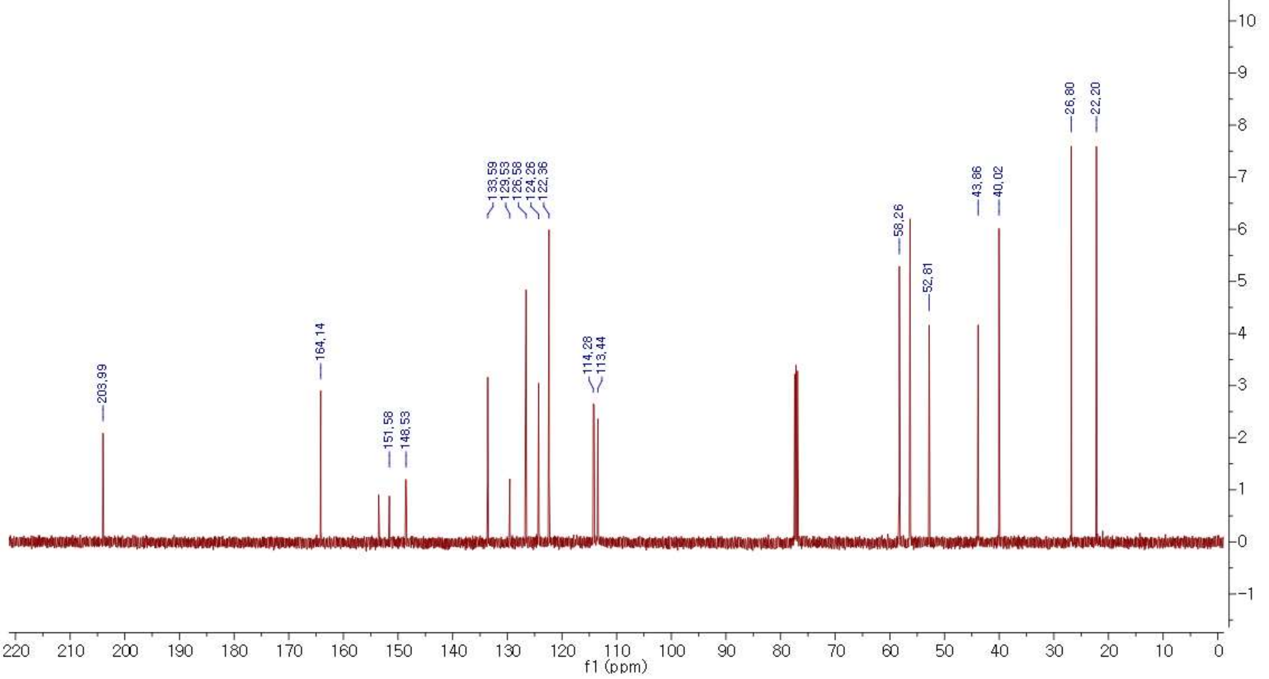

6g (LMT-409)

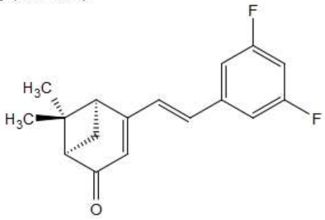

^{1}H NMR (500 MHz, cdcl$_3$) δ 7.09 – 6.89 (m, 3H), 6.87 – 6.66 (m, 2H), 5.96 (s, 1H), 3.14 – 3.01 (m, 1H), 2.99 – 2.85 (m, 1H), 2.80 – 2.66 (m, 1H), 2.10 (d, J = 9.2 Hz, 1H), 1.58 (s, 3H), 1.00 (s, 3H).

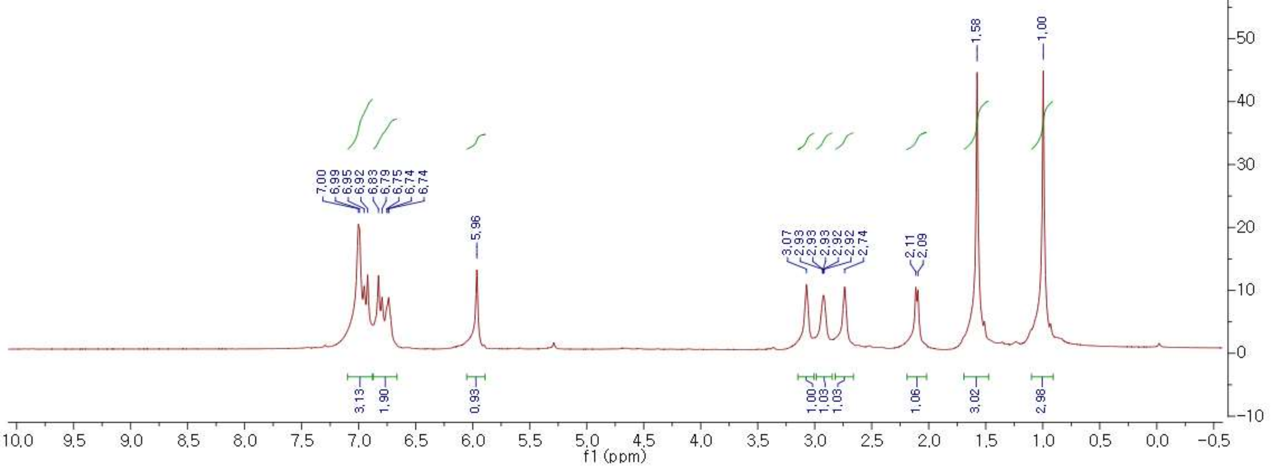

6g (LMT-409)

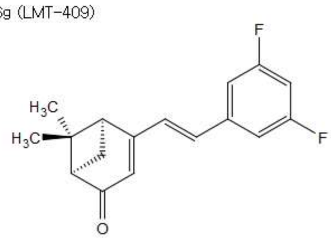

^{13}C NMR (126 MHz, cdcl$_3$) δ 203.80, 164.24, 163.14, 162.27, 139.34, 132.36, 129.85, 124.04, 110.00, 109.79, 104.13, 58.18, 52.93, 43.59, 39.98, 26.69, 22.11.

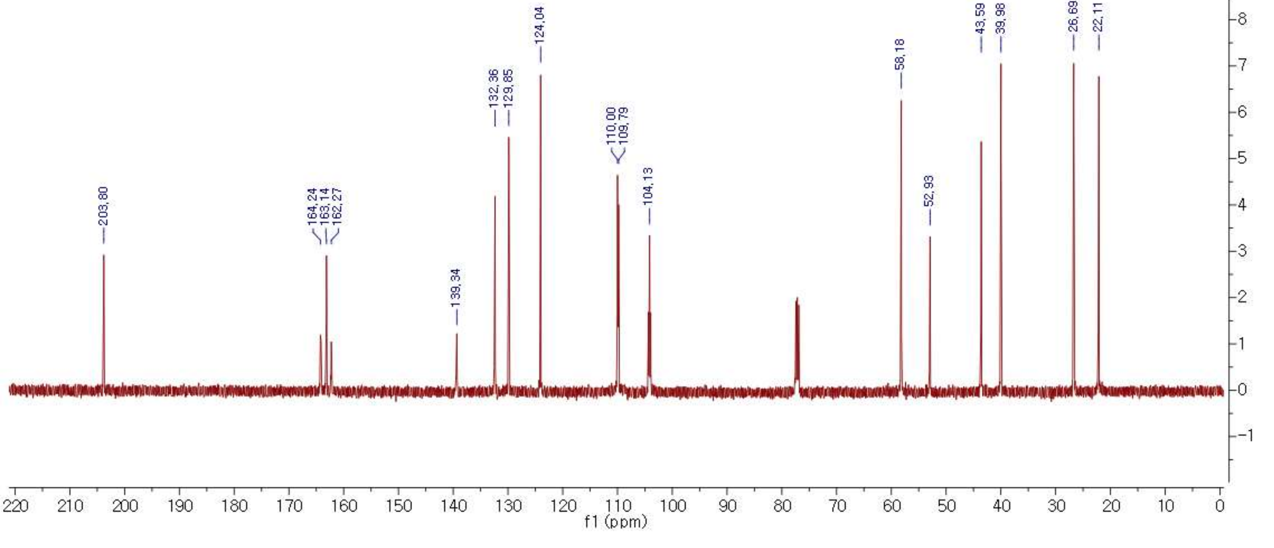

6h (LMT-411)

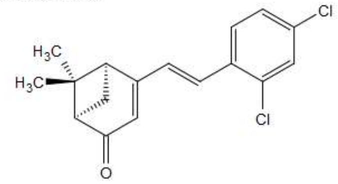

^{1}H NMR (500 MHz, cdcl$_3$) δ 7.59 (d, J = 8.2 Hz, 1H), 7.38 (s, 1H), 7.31 – 7.14 (m, 2H), 6.90 (d, J = 16.1 Hz, 1H), 5.95 (s, 1H), 3.19 – 3.04 (m, 1H), 3.01 – 2.84 (m, 1H), 2.80 – 2.64 (m, 1H), 2.11 (d, J = 9.2 Hz, 1H), 1.58 (s, 3H), 1.01 (s, 3H).

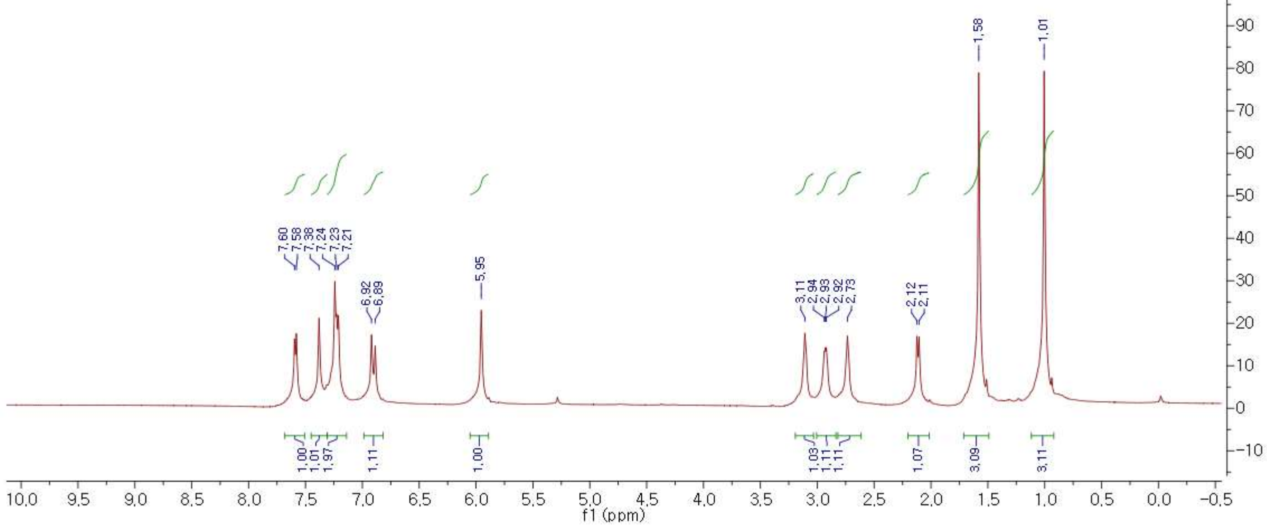

6h (LMT-411)

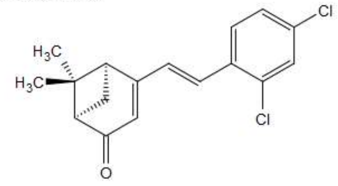

^{13}C NMR (126 MHz, cdcl$_3$) δ 203.93, 163.61, 134.99, 134.65, 132.63, 130.13, 129.80, 129.28, 127.62, 127.56, 123.92, 58.21, 52.98, 43.67, 40.08, 26.79, 22.17.

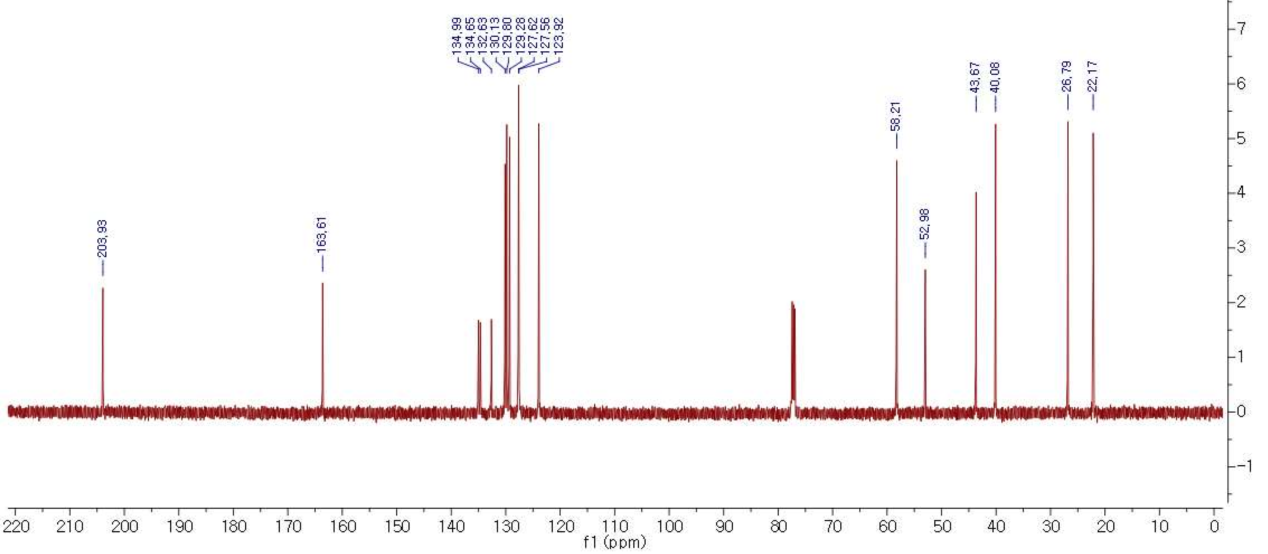

6i (LMT-410)

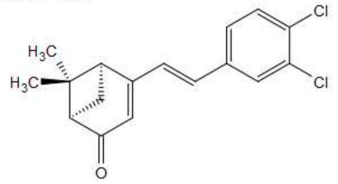

^{1}H NMR (500 MHz, cdcl$_3$) δ 7.56 (s, 1H), 7.41 (d, J = 8.2 Hz, 1H), 7.31 (d, J = 7.5 Hz, 1H), 6.85 (dd, J = 64.7, 16.1 Hz, 2H), 5.94 (s, 1H), 3.12 – 3.01 (m, 1H), 2.97 – 2.84 (m, 1H), 2.78 – 2.66 (m, 1H), 2.10 (d, J = 9.4 Hz, 1H), 1.57 (s, 3H), 0.99 (s, 3H).

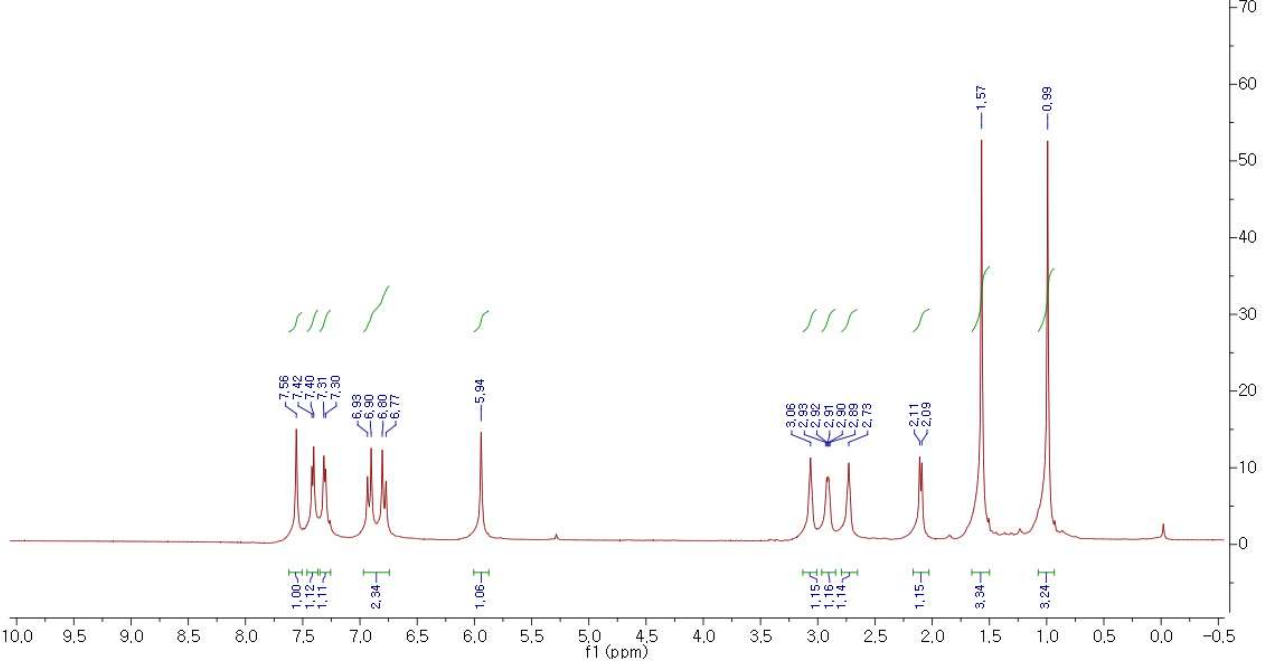

6i (LMT-410)

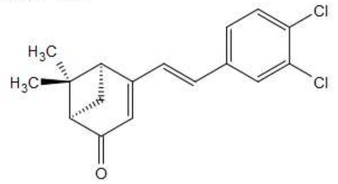

^{13}C NMR (126 MHz, cdcl$_3$) δ 203.98, 163.43, 136.15, 133.15, 132.81, 132.23, 130.85, 129.18, 128.97, 126.36, 123.74, 58.25, 53.00, 43.66, 40.08, 26.81, 22.22.

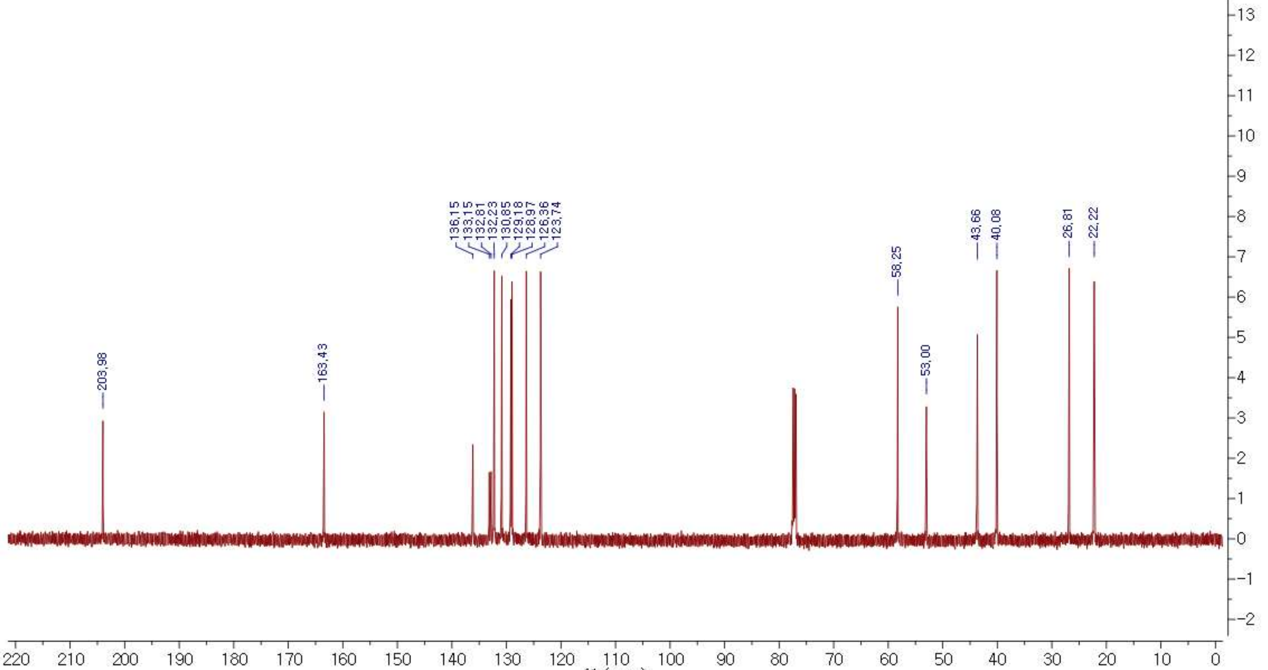

6j (LMT-422)

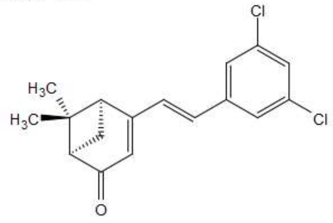

^{1}H NMR (500 MHz, cdcl$_3$) δ 7.33 (d, J = 1.8 Hz, 2H), 7.24 (t, J = 1.8 Hz, 1H), 6.84 (dd, J = 86.8, 16.1 Hz, 2H), 5.96 (s, 1H), 3.05 (td, J = 5.8, 1.1 Hz, 1H), 2.92 (dt, J = 9.5, 5.6 Hz, 1H), 2.73 (td, J = 5.7, 1.7 Hz, 1H), 2.10 (d, J = 9.5 Hz, 1H), 1.57 (s, 3H), 0.99 (s, 3H).

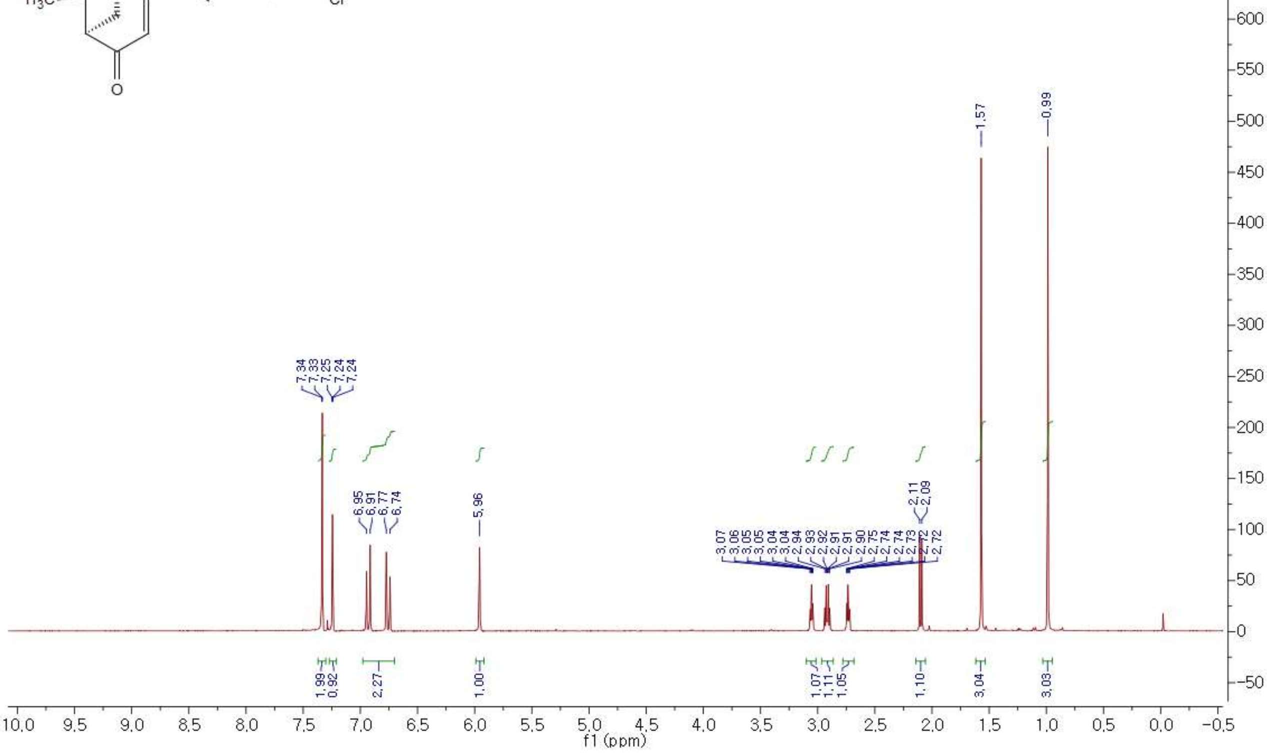

6j (LMT-422)

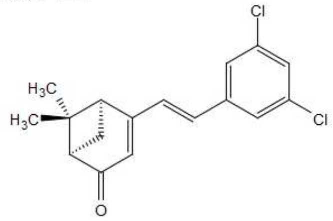

^{13}C NMR (126 MHz, cdcl$_3$) δ 203.72, 163.05, 138.97, 135.38, 131.80, 129.95, 128.52, 125.47, 124.08, 58.16, 52.91, 43.55, 39.96, 26.71, 22.13.

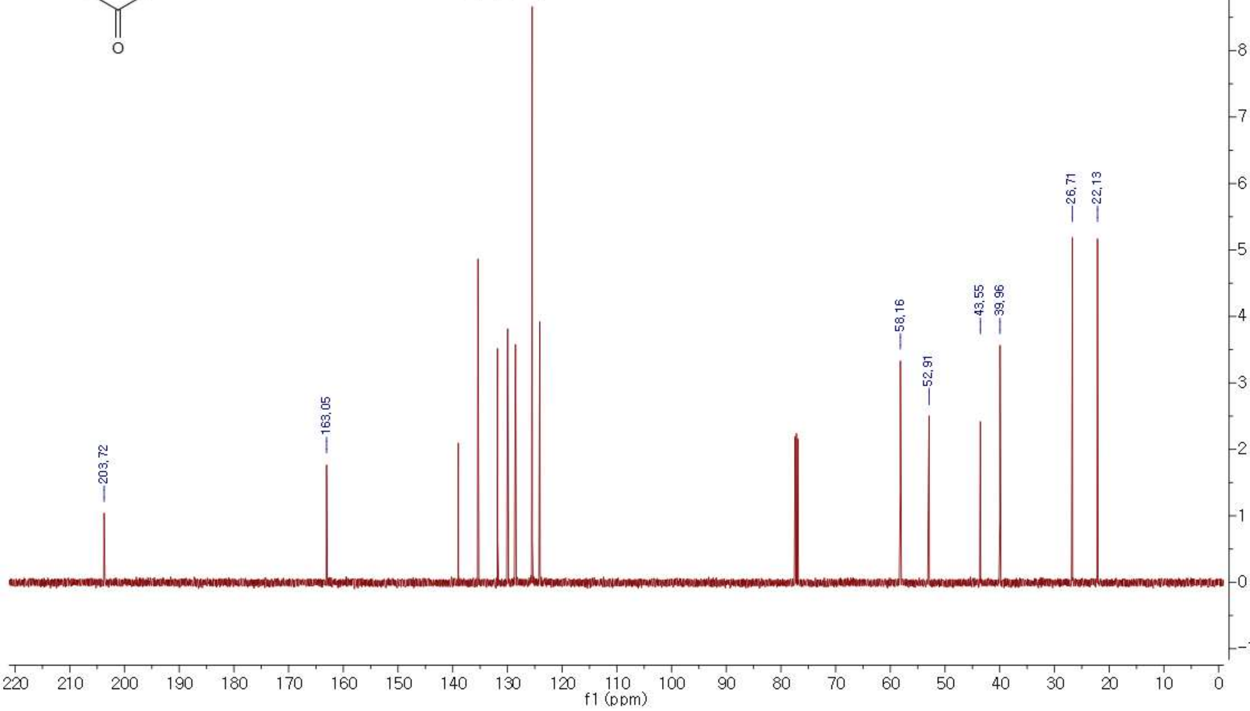

6k (LMT-474)

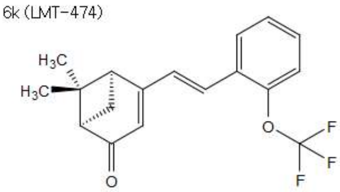

^{1}H NMR (500 MHz, cdcl$_3$) δ 7.70 (dd, J = 7.4, 2.1 Hz, 1H), 7.30 (pd, J = 7.3, 1.7 Hz, 2H), 7.26 – 7.21 (m, 1H), 7.07 (dd, J = 85.2, 16.3 Hz, 2H), 5.96 (s, 1H), 3.09 (t, J = 5.8 Hz, 1H), 2.90 (dt, J = 9.5, 5.6 Hz, 1H), 2.72 (td, J = 5.7, 1.5 Hz, 1H), 2.08 (d, J = 9.4 Hz, 1H), 1.56 (s, 3H), 0.99 (s, 3H).

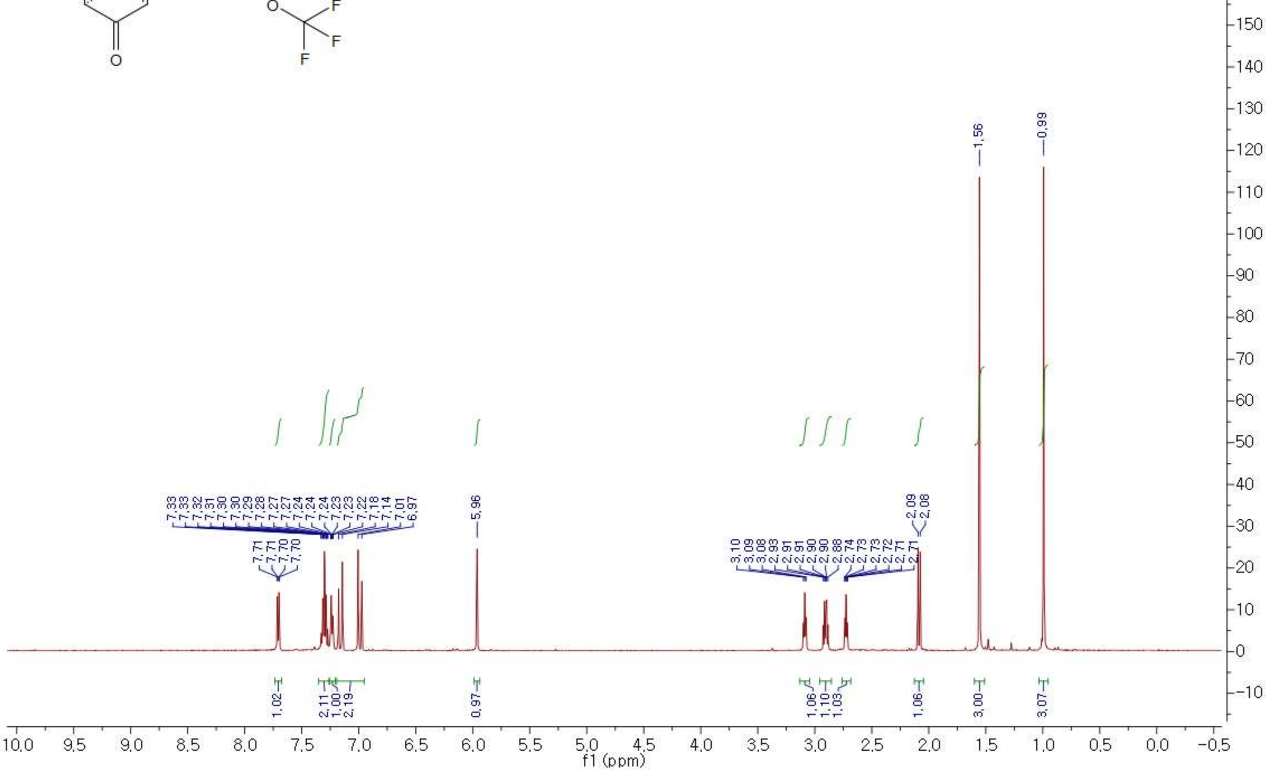

6k (LMT-474)

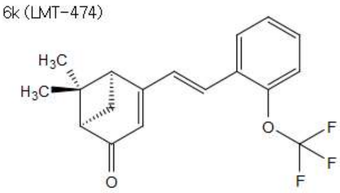

^{13}C NMR (126 MHz, cdcl$_3$) δ 203.21, 163.42, 146.76, 129.82, 129.77, 129.21, 127.00, 126.88, 126.78, 123.39, 121.14, 121.13, 58.00, 52.54, 43.53, 39.58, 26.38, 21.73.

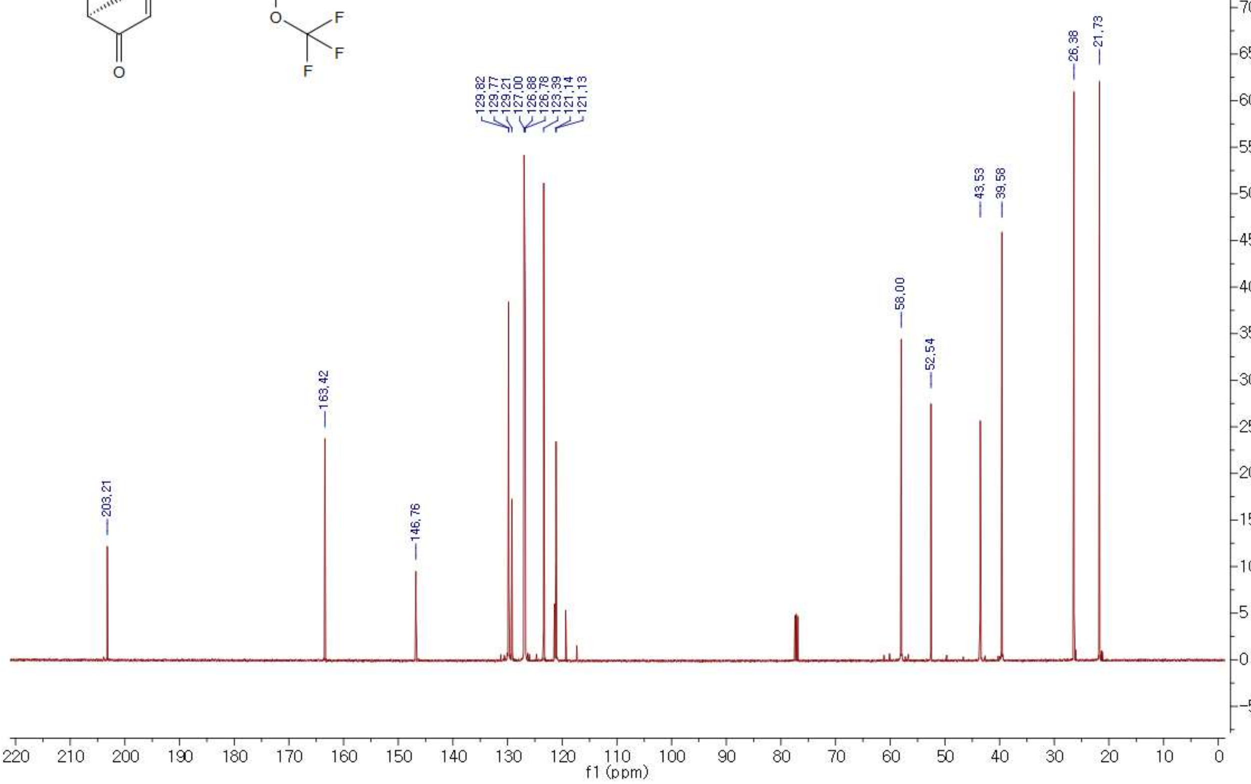

6l (LMT-473)

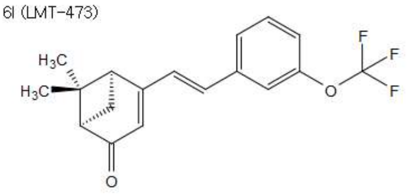

^{1}H NMR (500 MHz, cdcl$_3$) δ 7.42 (dt, J = 7.7, 1.1 Hz, 1H), 7.37 (t, J = 7.9 Hz, 1H), 7.32 (s, 1H), 7.17 – 7.11 (m, 1H), 6.92 (dd, J = 37.5, 16.2 Hz, 2H), 5.95 (s, 1H), 3.09 (td, J = 5.8, 1.3 Hz, 1H), 2.91 (dt, J = 9.4, 5.6 Hz, 1H), 2.73 (td, J = 5.7, 1.7 Hz, 1H), 2.10 (d, J = 9.4 Hz, 1H), 1.57 (s, 3H), 1.00 (s, 3H).

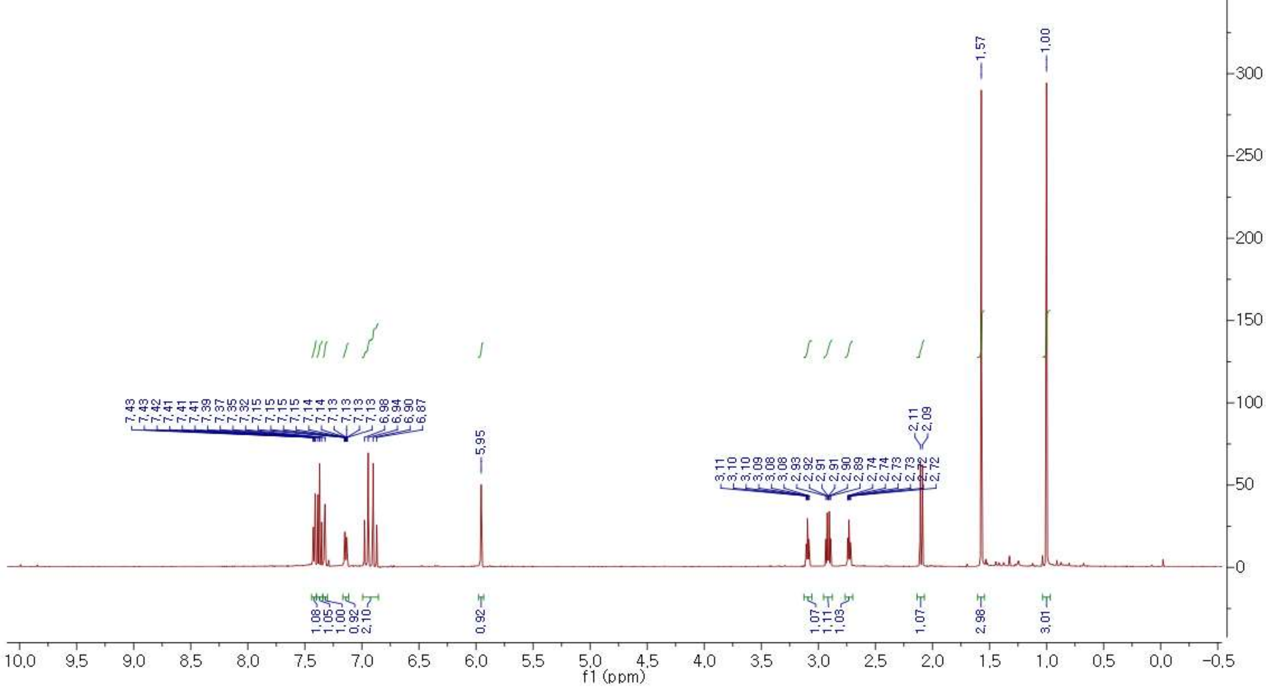

6l (LMT-473)

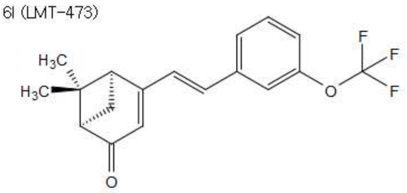

^{13}C NMR (126 MHz, cdcl$_3$) δ 203.81, 163.56, 149.75, 138.23, 133.19, 130.23, 129.04, 125.63, 123.53, 121.17, 119.52, 58.27, 52.87, 43.78, 39.98, 26.69, 22.11.

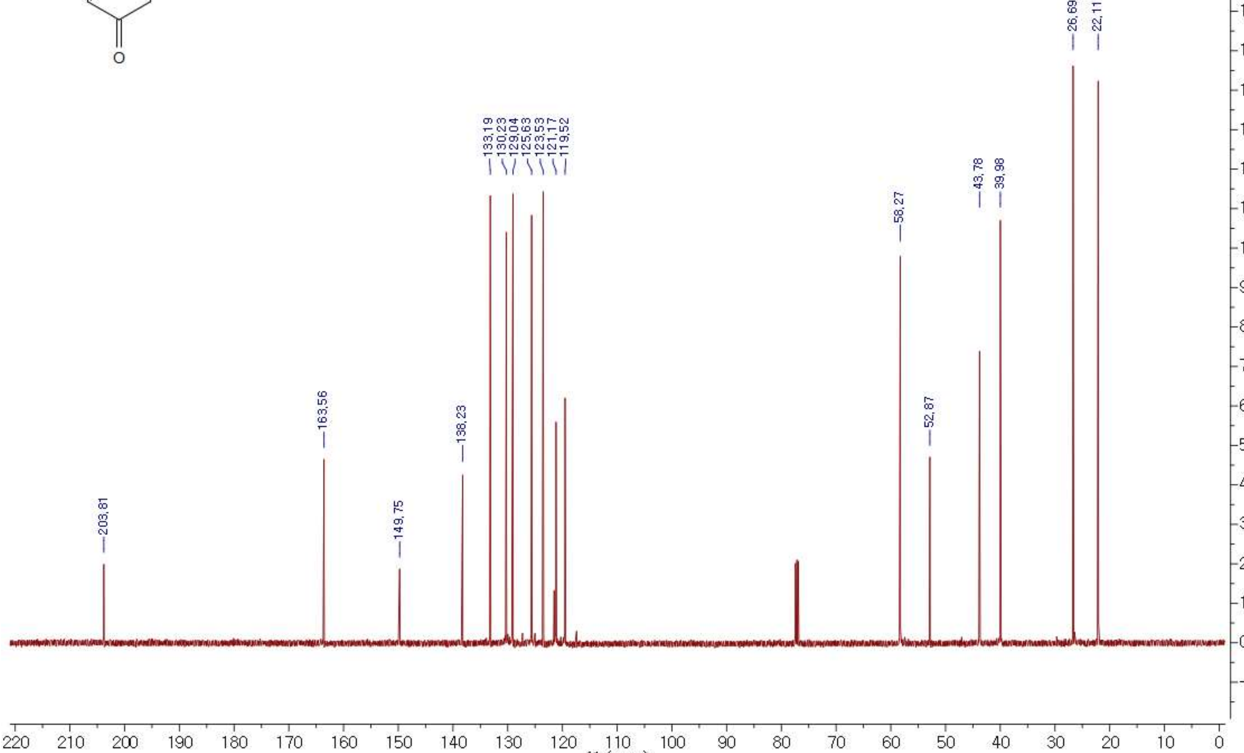

6m (LMT-472)

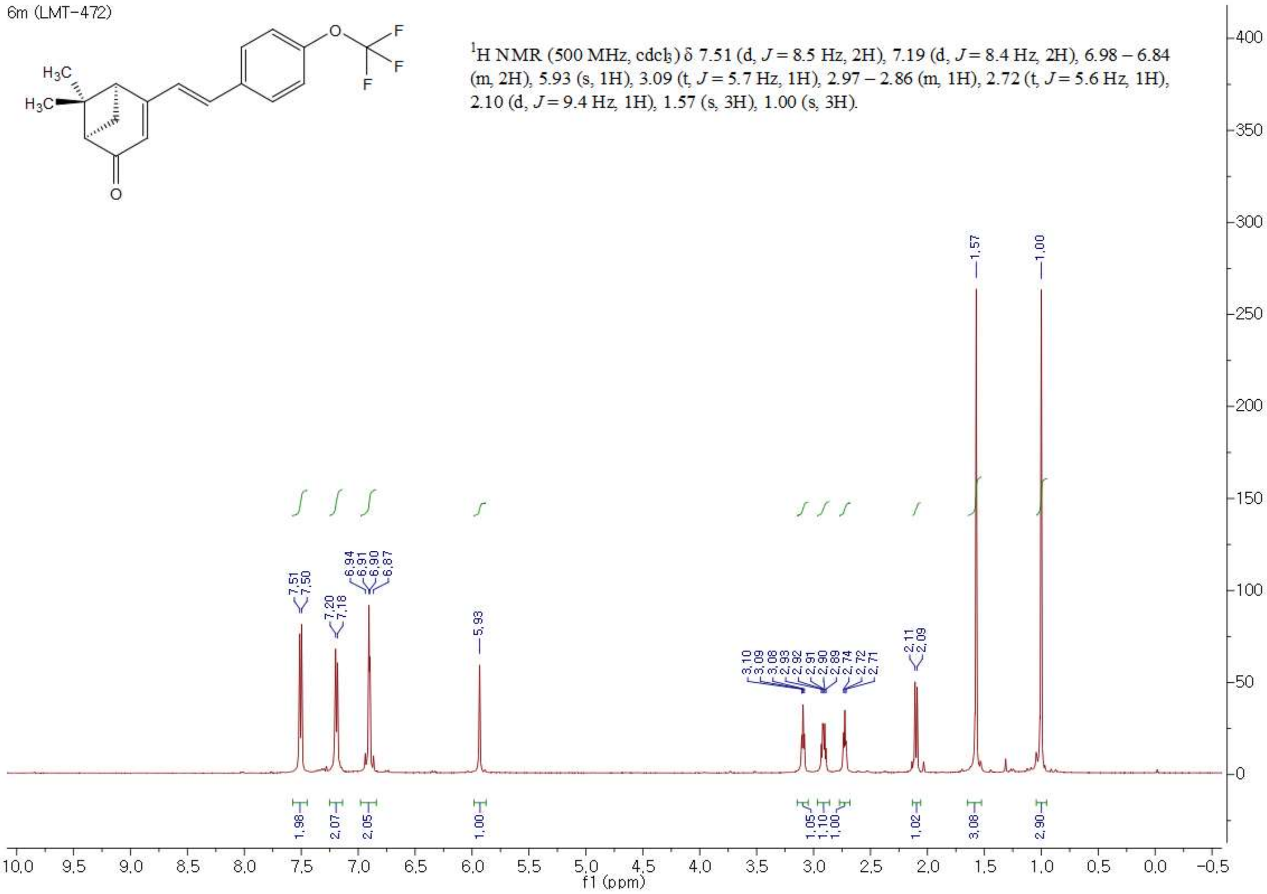

^{1}H NMR (500 MHz, cdcl$_3$) δ 7.51 (d, J = 8.5 Hz, 2H), 7.19 (d, J = 8.4 Hz, 2H), 6.98 – 6.84 (m, 2H), 5.93 (s, 1H), 3.09 (t, J = 5.7 Hz, 1H), 2.97 – 2.86 (m, 1H), 2.72 (t, J = 5.6 Hz, 1H), 2.10 (d, J = 9.4 Hz, 1H), 1.57 (s, 3H), 1.00 (s, 3H).

6m (LMT-472)

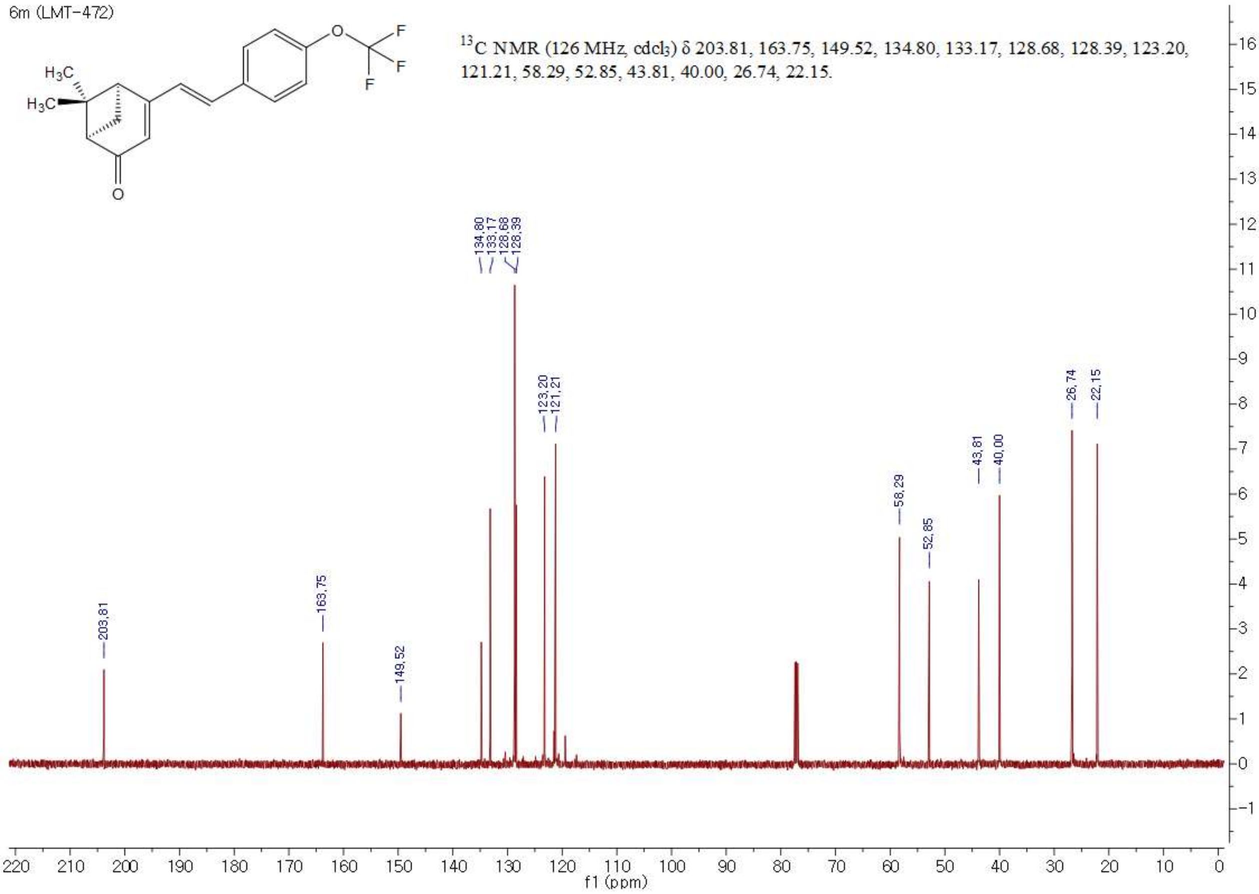

^{13}C NMR (126 MHz, cdcl$_3$) δ 203.81, 163.75, 149.52, 134.80, 133.17, 128.68, 128.39, 123.20, 121.21, 58.29, 52.85, 43.81, 40.00, 26.74, 22.15.

6n (LMT-404)

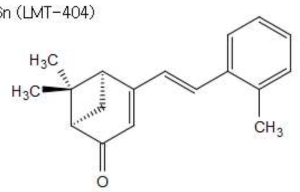

^{1}H NMR (500 MHz, cdcl$_3$) δ 7.60 – 7.51 (m, 1H), 7.25 – 7.10 (m, 4H), 6.85 (d, J = 16.0 Hz, 1H), 5.91 (s, 1H), 3.10 (t, J = 5.7 Hz, 1H), 2.91 (dt, J = 9.4, 5.6 Hz, 1H), 2.75 – 2.67 (m, 1H), 2.38 (s, 3H), 2.12 (d, J = 9.4 Hz, 1H), 1.57 (s, 3H), 1.01 (s, 3H).

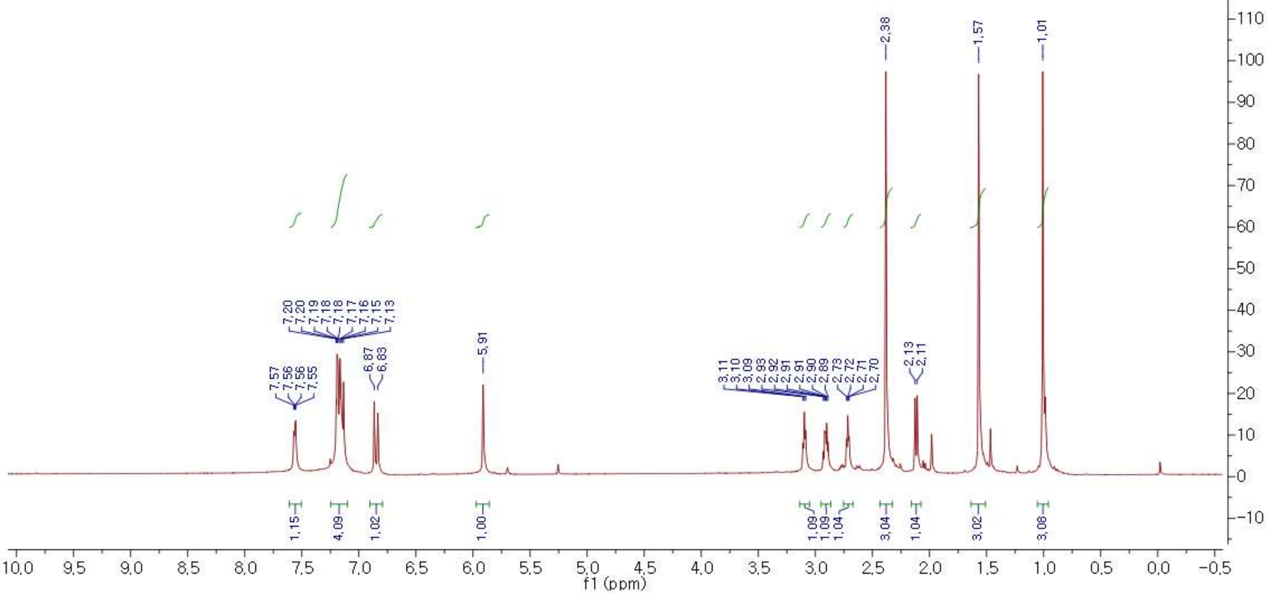

6n (LMT-404)

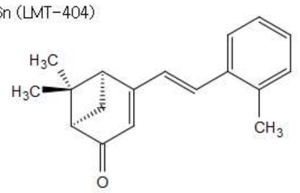

^{13}C NMR (126 MHz, cdcl$_3$) δ 204.15, 164.53, 136.61, 134.85, 132.42, 130.70, 128.97, 128.50, 126.40, 125.73, 122.55, 58.17, 52.92, 43.82, 40.07, 26.82, 22.15, 19.85.

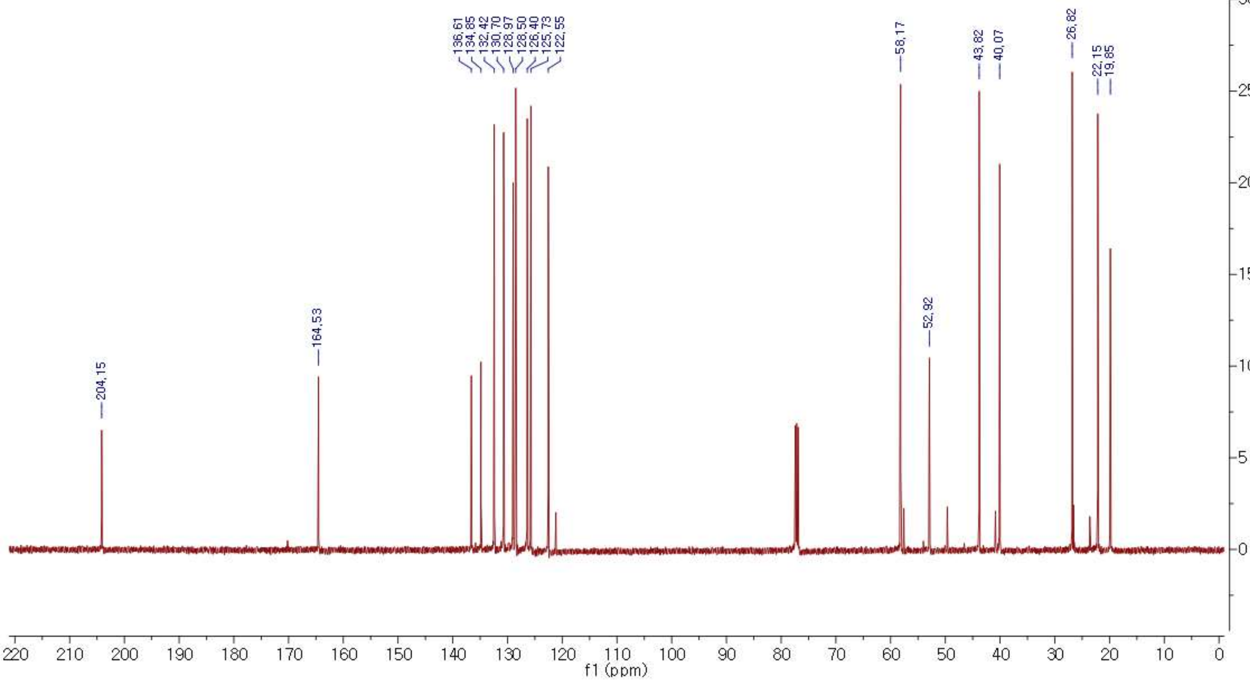

6o (LMT-403)

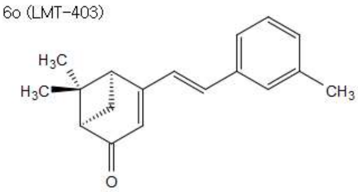

^{1}H NMR (500 MHz, cdcl$_3$) δ 7.37 – 7.19 (m, 3H), 7.12 (d, *J* = 7.3 Hz, 1H), 6.91 (q, *J* = 16.2 Hz, 2H), 5.91 (s, 1H), 3.11 (t, *J* = 5.8 Hz, 1H), 2.90 (dt, *J* = 9.4, 5.6 Hz, 1H), 2.72 (td, *J* = 5.8, 1.7 Hz, 1H), 2.35 (s, 3H), 2.10 (d, *J* = 9.4 Hz, 1H), 1.56 (s, 3H), 1.00 (s, 3H).

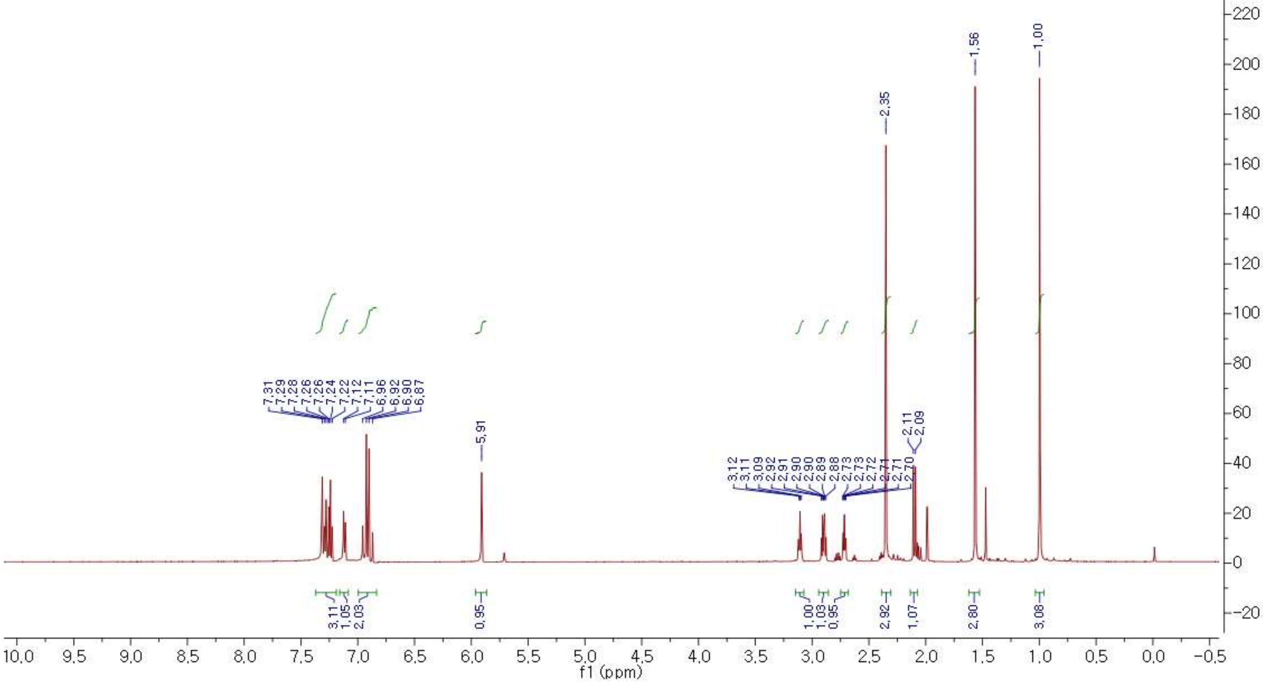

6o (LMT-403)

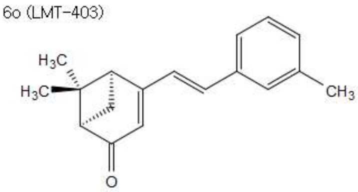

^{13}C NMR (126 MHz, cdcl$_3$) δ 204.20, 164.41, 138.46, 135.87, 135.22, 130.04, 128.77, 128.00, 127.14, 124.66, 122.47, 58.18, 52.87, 43.64, 40.02, 26.78, 22.16, 21.40.

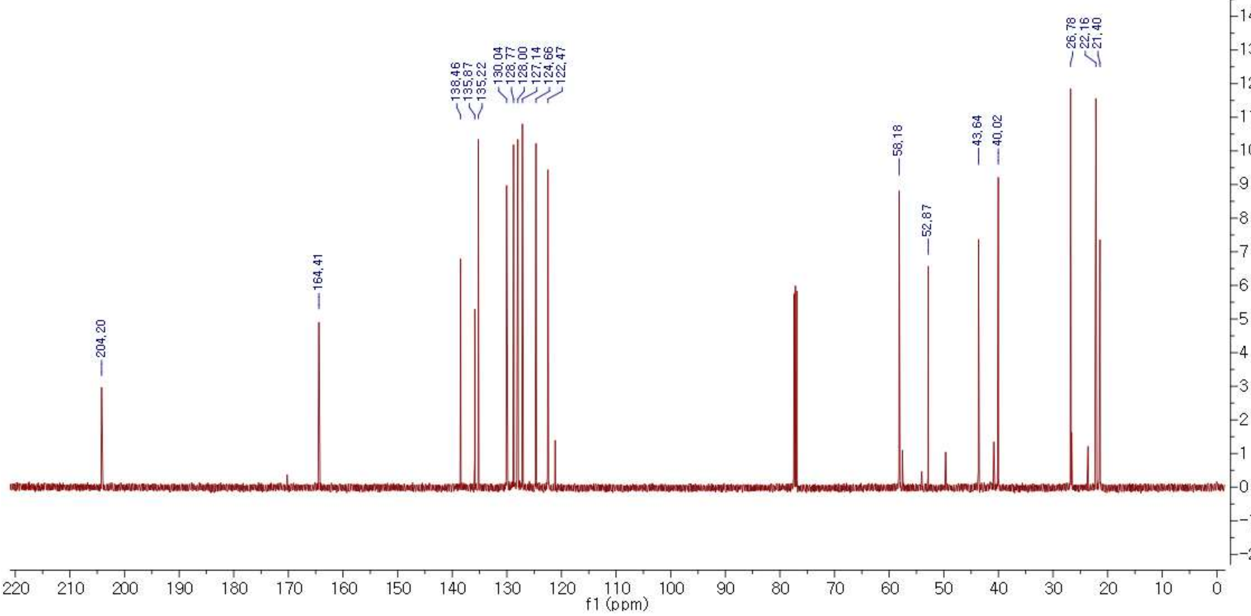

6p (LMT-402)

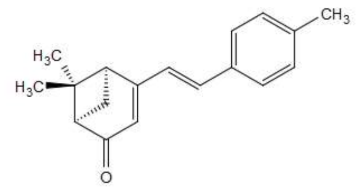

^{1}H NMR (500 MHz, cdcl$_3$) δ 7.38 (d, J = 8.1 Hz, 2H), 7.16 (d, J = 7.8 Hz, 2H), 6.89 (s, 2H), 5.89 (s, 1H), 3.10 (t, J = 5.8 Hz, 1H), 2.89 (dt, J = 9.4, 5.6 Hz, 1H), 2.71 (td, J = 5.8, 1.7 Hz, 1H), 2.34 (s, 3H), 2.09 (d, J = 9.4 Hz, 1H), 1.56 (s, 3H), 1.00 (s, 3H).

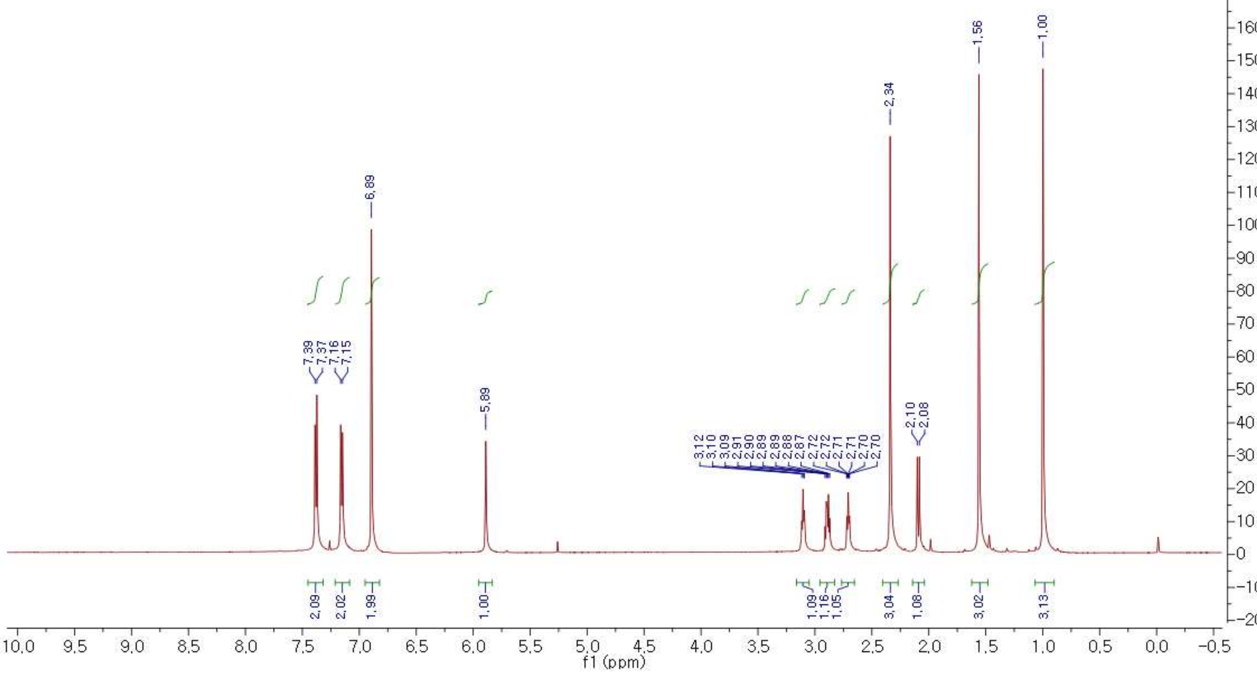

6p (LMT-402)

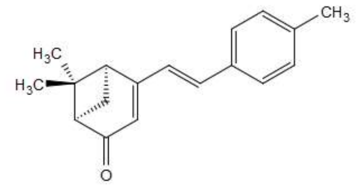

^{13}C NMR (126 MHz, cdcl$_3$) δ 204.19, 164.52, 139.40, 135.07, 133.19, 129.62, 127.34, 126.35, 122.16, 58.16, 52.81, 43.63, 40.02, 26.77, 22.16, 21.43.

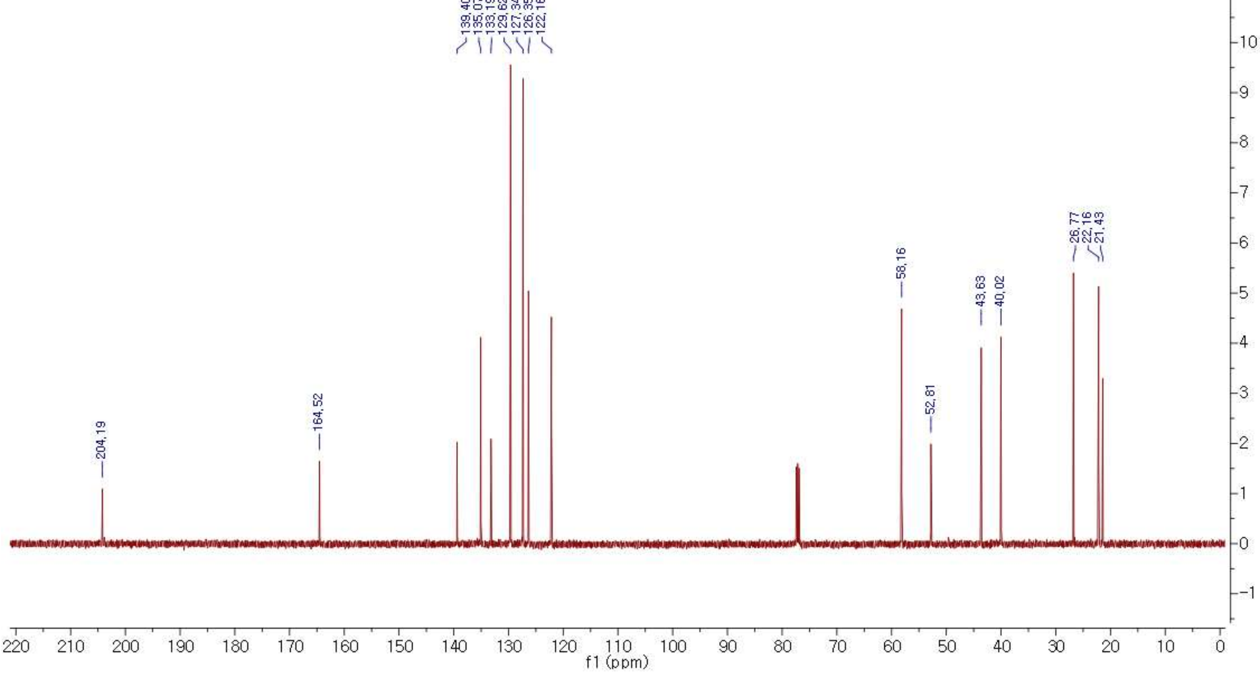

6q (LMT-408)

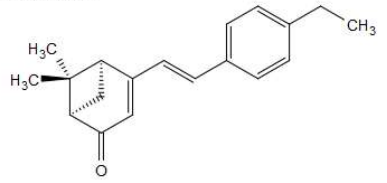

^{1}H NMR (500 MHz, cdcl$_3$) δ 7.39 (d, J = 8.1 Hz, 2H), 7.16 (d, J = 8.0 Hz, 2H), 6.89 (s, 2H), 5.88 (s, 1H), 3.15 – 3.04 (m, 1H), 2.87 (dt, J = 9.4, 5.6 Hz, 1H), 2.74 – 2.66 (m, 1H), 2.64 – 2.54 (m, 2H), 2.07 (d, J = 9.3 Hz, 1H), 1.54 (s, 3H), 1.20 (t, J = 7.6 Hz, 3H), 0.98 (s, 3H).

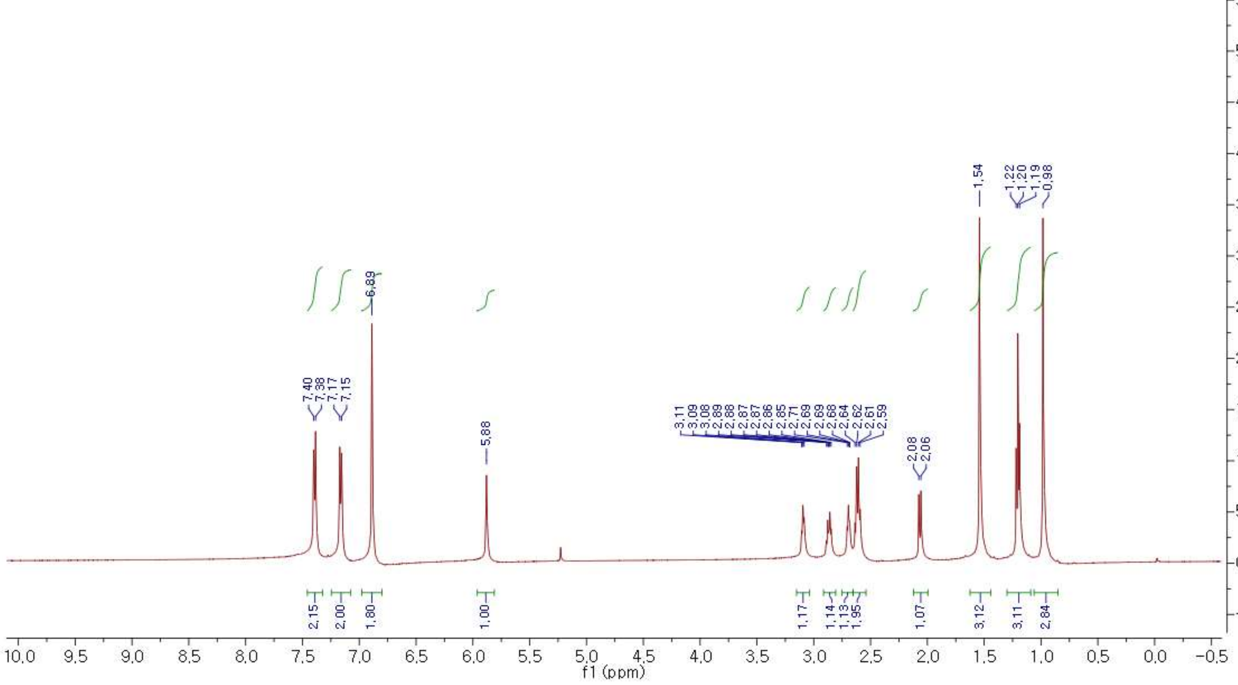

6q (LMT-408)

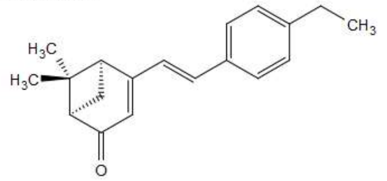

^{13}C NMR (126 MHz, cdcl$_3$) δ 203.84, 164.32, 145.53, 134.93, 133.28, 128.25, 127.29, 126.21, 121.97, 57.98, 52.58, 43.43, 39.80, 28.57, 26.59, 21.98, 15.29.

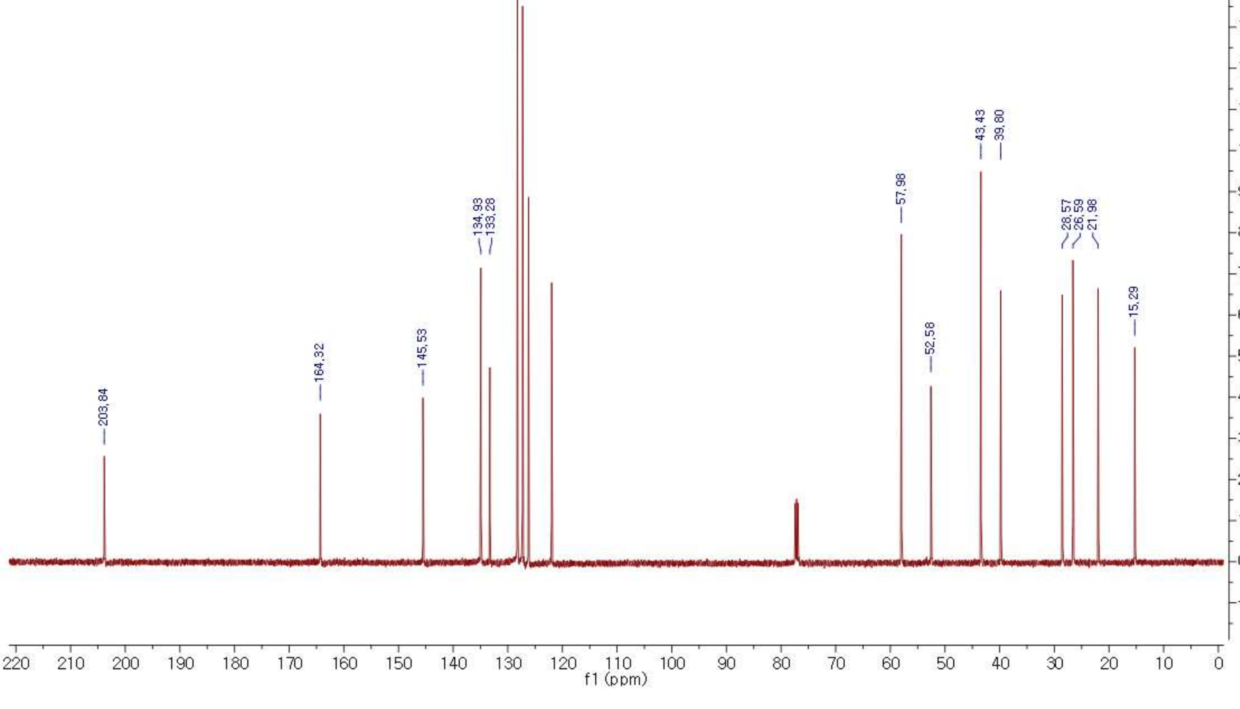

7a (LMT-489)

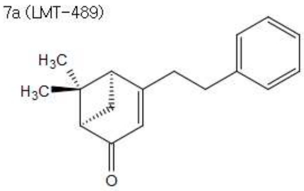

^{1}H NMR (500 MHz, cdcl$_3$) δ 7.31 – 7.26 (m, 2H), 7.22 – 7.16 (m, 3H), 5.75 (p, J = 1.5 Hz, 1H), 2.83 – 2.77 (m, 3H), 2.67 – 2.63 (m, 1H), 2.62 – 2.56 (m, 2H), 2.51 – 2.47 (m, 1H), 2.05 (d, J = 9.2 Hz, 1H), 1.50 (s, 3H), 1.00 (s, 3H).

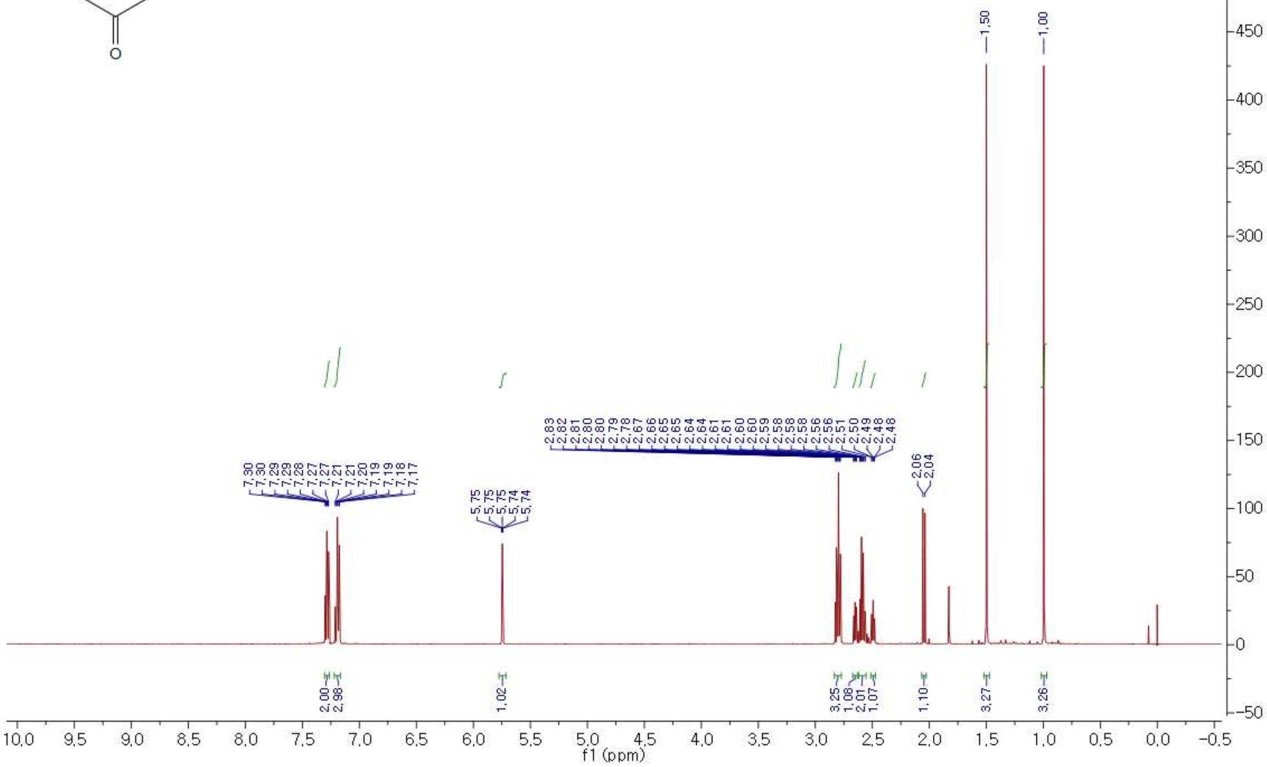

7a (LMT-489)

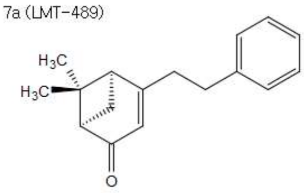

^{13}C NMR (126 MHz, cdcl$_3$) δ 203.97, 172.41, 140.72, 128.60, 128.29, 126.34, 120.52, 57.97, 54.05, 48.89, 41.08, 38.67, 32.78, 26.69, 22.42.

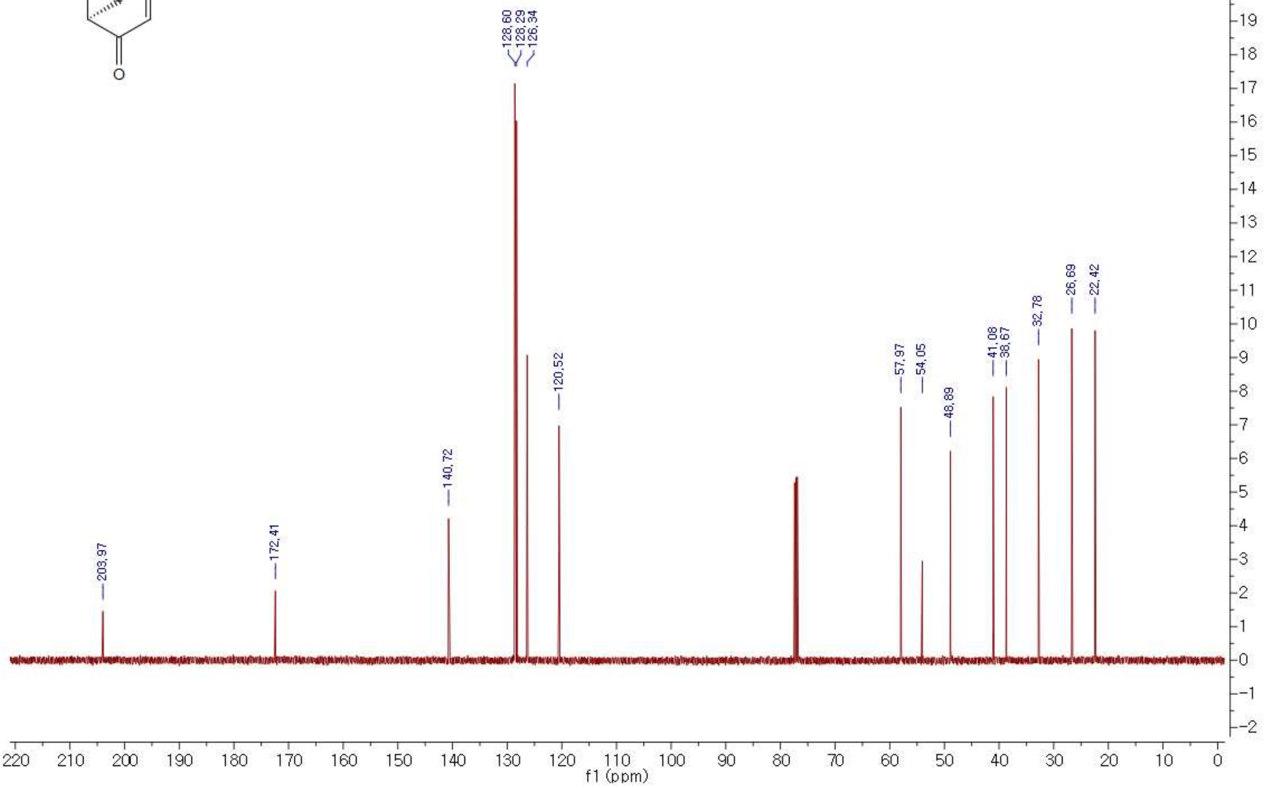

7b (LMT-490)

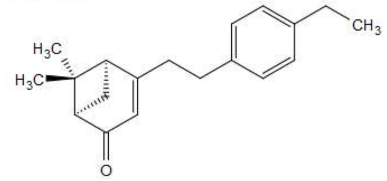

^{1}H NMR (500 MHz, cdcl$_3$) δ 7.11 (q, J = 8.1 Hz, 4H), 5.75 (s, 1H), 2.79 (ddd, J = 25.7, 13.3, 6.7 Hz, 3H), 2.68 – 2.53 (m, 5H), 2.50 (t, J = 5.7 Hz, 1H), 2.05 (d, J = 9.1 Hz, 1H), 1.50 (s, 3H), 1.23 (t, J = 7.6 Hz, 3H), 1.00 (s, 3H).

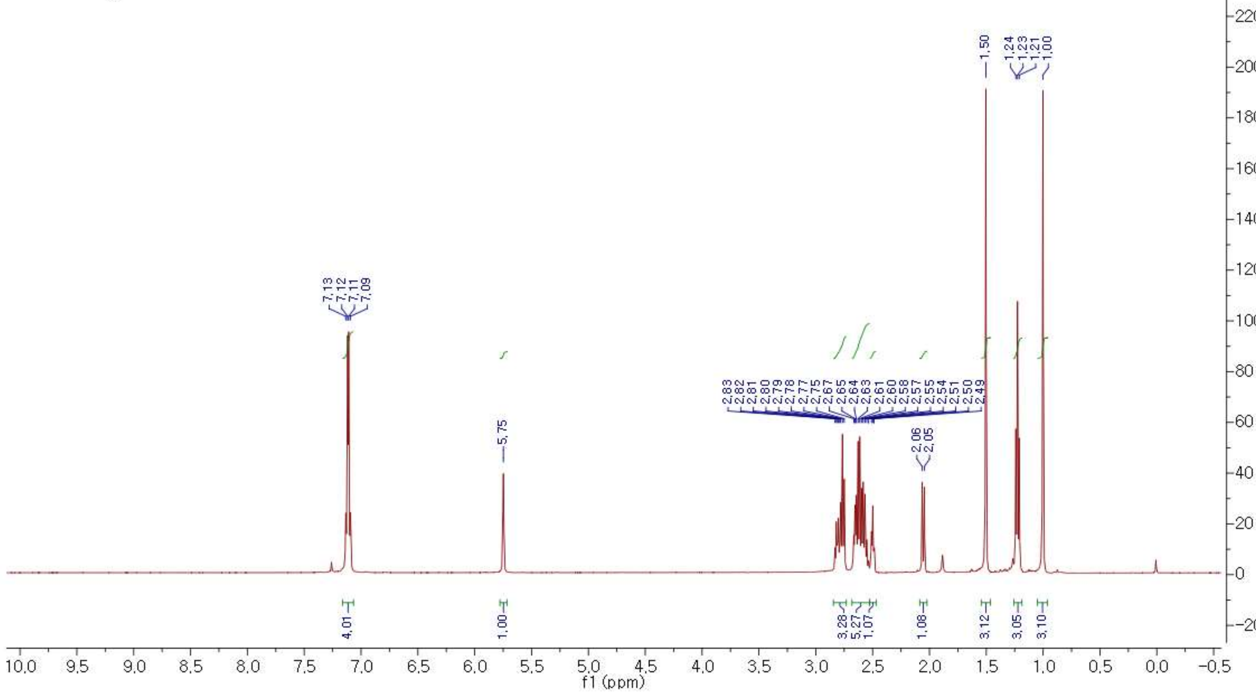

7b (LMT-490)

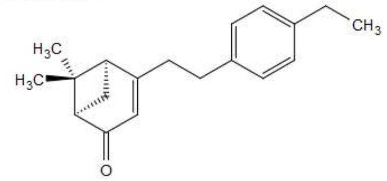

^{13}C NMR (126 MHz, cdcl$_3$) δ 203.99, 172.57, 142.24, 137.87, 128.20, 128.05, 120.45, 57.96, 54.02, 48.88, 41.07, 38.78, 32.35, 28.49, 26.68, 22.41, 15.67.

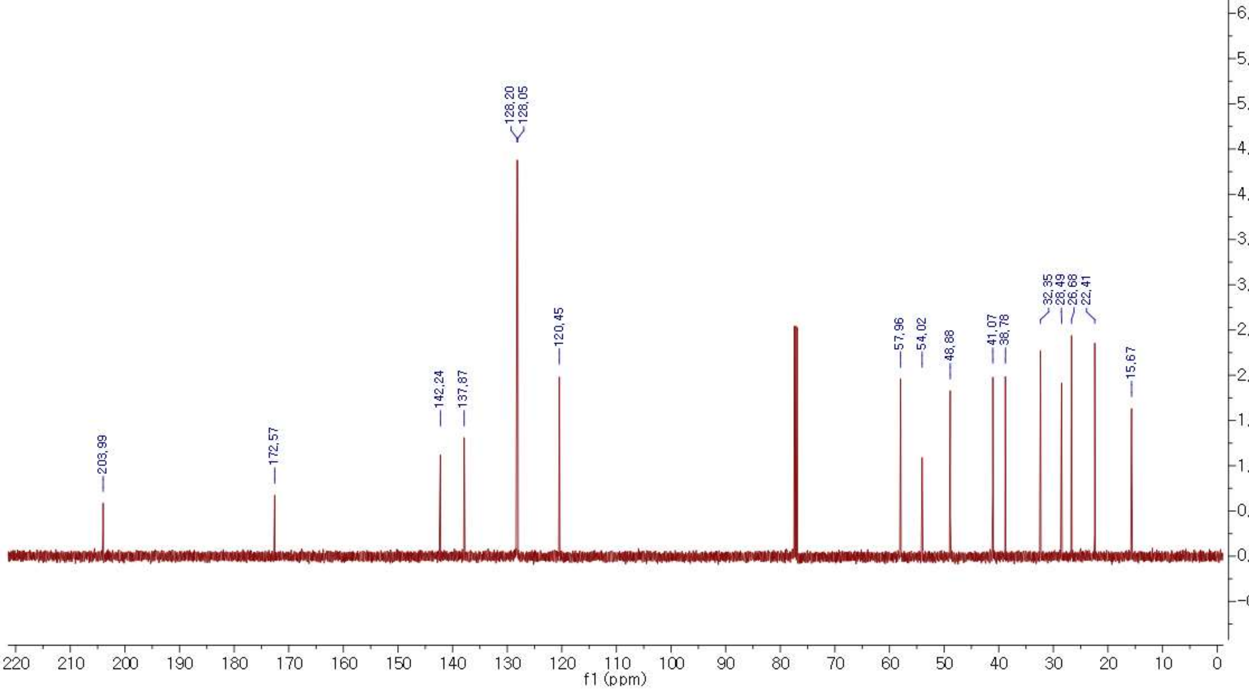

Reference

1. Islam, S.; Paul, S.; Roy, A.S.; Banerjee, S.; Ghosh, K.; Dey, R.C.; Santra, S.C. Catalytic activity of an iron (III) Schiff base complex bound in a polymer resin. *Transition Met. Chem.* **2013**, *38*, 675-682.
